# A pilot randomized controlled trial of group-based indoor gardening and art activities demonstrates therapeutic benefits to healthy women

**DOI:** 10.1371/journal.pone.0269248

**Published:** 2022-07-06

**Authors:** Raymond Odeh, Elizabeth R. M. Diehl, Sara Jo Nixon, C. Craig Tisher, Dylan Klempner, Jill K. Sonke, Thomas A. Colquhoun, Qian Li, Maria Espinosa, Dianela Perdomo, Kaylee Rosario, Hannah Terzi, Charles L. Guy

**Affiliations:** 1 Department of Environmental Horticulture, University of Florida, Gainesville, Florida, United States of America; 2 Wilmot Botanical Gardens, University of Florida, Gainesville, Florida, United States of America; 3 Department of Psychiatry, University of Florida, Gainesville, Florida, United States of America; 4 Center for Arts in Medicine, University of Florida, Gainesville, Florida, United States of America; 5 Health Outcomes & Biomedical Informatics, University of Florida, Gainesville, Florida, United States of America; Prince Sattam Bin Abdulaziz University, College of Applied Medical Sciences, SAUDI ARABIA

## Abstract

**Background:**

There is mounting anecdotal and empirical evidence that gardening and art-making afford therapeutic benefits.

**Objectives:**

This randomly controlled pilot study tested the hypothesis that participation in group-based indoor gardening or art-making activities for one hour twice a week for four weeks would provide quantifiably different therapeutic benefits to a population of healthy women ages 26–49.

**Methods:**

A population of 42 volunteers was randomly assigned to parallel gardening or art-making treatment groups. A total of 36 participants initiated the treatment protocol and 32 (Gardening n = 15 and Art n = 17) received the interventions and completed all assessments. Treatments included eight one-hour group-based gardening or art intervention sessions. Self-report psychometric assessments were conducted for anxiety, depression symptomatology, mood disturbance, stress, satisfaction with discretionary social activities, and quality of life measures. Cardiac physiological data were also collected. Outcomes were measured at baseline, during, and post-intervention.

**Results:**

Engaging in both gardening and art-making activities resulted in apparent therapeutic improvements for self-reported total mood disturbance, depression symptomatology, and perceived stress with different effect sizes following eight one-hour treatment sessions. Gardening also resulted in improvements for indications of trait anxiety. Based on time-course evidence, dosage responses were observed for total mood disturbance, perceived stress, and depression symptomatology for both gardening and art-making. However, gardening or art-making did not have an apparent influence on heart rate or blood pressure or result in marked improvement for satisfaction with discretionary leisure activities.

**Conclusion:**

The data did not support the hypothesis of differential therapeutic benefits of gardening and art-making for healthy women. When taken together, group-based gardening or art-making can provide quantitatively measurable improvements in healthy women’s psychosocial health status that imply potentially important public health benefits.

**Trial registration:**

ClinicalTrials.gov NCT03266120.

## Introduction

Plants as autotrophic organisms provide the foundations of life for humans and most other heterotrophic life forms on Earth. Moreover, plants have been an essential part of the human condition over the last two million years, and their collective role in our survival, evolution, and cognitive development is unparalleled [1]. The early stages of plant cultivation in the Upper Paleolithic Period and domestication of plants and farming in the Neolithic Period [2] gave rise to the emergence of cities and civilizations. Thus, the cultivation of plants, gardens, and gardening has been an enduring integral factor in our adaptive ability and well-being as a species. A case has been made that through learning and evolutionary processes, interactions with natural environments and living organisms, and our dependence upon nature for various ecological services, we are programmed to be innately attracted to plants and nature [3]. However, connections with nature in the modern world have become fewer over time as global urbanization has resulted in increasing barriers to the accessibility of plants and natural environments. Visiting and experiencing gardens and gardening are two types of people-plant interactions that can provide access to nature for urban populations.

The observation that gardening or engaging in horticultural activities could be therapeutic in the United States dates to the early 19th century [4]. Modern therapeutic horticulture (TH) in its most common form is primarily a combination of gardening and general horticultural activities designed based on program goals and facilitated by a professional with training in the use of horticulture as a therapeutic modality [5]. Horticultural therapy (HT) extends TH with a planned clinical modality, individualized treatment plans, and documented therapeutic goals and outcomes. Horticultural therapy and TH are now widely recognized and practiced in developed countries worldwide [6]. Nevertheless, virtually all systematic reviews and meta-analyses of the published scientific literature on the therapeutic benefits of gardening and HT have revealed a surprising paucity of high-quality experimental research [7–16]. Clearly, there is a demonstrated need for more research that better defines quantitative treatment effects and the actual health benefits that gardening and horticultural activities may provide. Until there have been large-scale high quality randomized controlled trials of HT and/or gardening their actual clinical significance as treatment modalities will remain unknown.

Art-making, similar to gardening, is thought to be an innate behavior of humans [17, 18], and both art-making and art therapy (AT) have been shown to provide therapeutic benefits across a range of clinical settings [19–34]. Art therapy is centered on the idea that art-making is often a nonverbal form of communication of one’s thoughts and feelings involving a creative process that fosters healing, personal growth, self-understanding, quality of life improvements, and transient respite from life’s ongoing challenges [35]. The central conceptual framework in the development of art as therapy was formulated during the 1940s to the 1970s [18]. Historically psychiatric hospitals and, more recently, hospitals, in general, have been instrumental in the development of the field of AT and leading to the modern medical specialty of arts in medicine. Based on systematic reviews and a meta-analysis of the art therapy literature related to non-psychotic mental health disorders and breast cancer populations, comparison of studies was limited to those related to mental health disorders due to the lack of comparable data and heterogeneity of clinical trials [24, 26, 28, 29]. In 10 of 15 randomized controlled trials reviewed by Uttley and colleagues [28], art therapy was associated with significant positive health outcomes relative to control conditions. Boehm and colleagues [26] concluded art therapy was an effective treatment for anxiety reduction in breast cancer patients based on results from four randomized controlled trials. Overall, the literature on art therapy encompasses varied diseases and disorders, demographics, research methodologies, and outcome measures, leading to gaps in our understanding of art therapy’s efficacy. Multicentered, mixed-methods randomized control trials with long-term follow-up repeated measures are needed to strengthen both art therapy and horticultural therapy published experimental research.

Virtually all studies aimed at defining the therapeutic benefits of gardening, HT, AT and art-making have been focused on populations experiencing health or physical challenges of one type or another. Therefore, little evidence of the therapeutic benefits on healthy populations is available in the literature. This study was conceived to measure the relative psychological, physiological, and social effects associated with the engagement of healthy women in group-based gardening or art-making activities. The following hypotheses were tested: 1. A group-based gardening intervention will impart different therapeutic outcomes related to self-reported mood states, perceived stress, depression symptomatology, anxiety, and overall health status for healthy women than a group-based art-making intervention. 2. Self-reported satisfaction with participation in discretionary social activities will improve for both group-based gardening and art interventions. 3. Engaging in group-based gardening and art interventions will lower heart rate and systolic and diastolic blood pressure for healthy women. Statistical analyses of treatment influenced-group change pre-to-post and between the two interventions permitted the testing of the three hypotheses.

## Materials and methods

### Sample population and study participants

Eligibility criteria for this healthy women-only study included: pre-menopausal women between the ages of 26 and 49 years, without any chronic conditions that negatively affect daily life, with a body mass index of <32, and without allergies to pollen, plants, or plant-based foods, a non-smoker, non-gardener, non-artist, and not abusing alcohol, prescription medications or using recreational drugs. The single-sex study population was selected to reduce experimental variability and increase the likelihood of revealing significant treatment effects [36]. Limiting health-related risk factors such as obesity, smoking, alcohol consumption, and drug use was intended to reduce experimental variability and embody a more homogeneous wellness study population allowing for greater internal validity of the experiment. It was, however, recognized that over the course of the experimental treatment, participants’ menstrual cycle might contribute to variability in some experimental results.

### Recruitment and intervention assignment

Study protocols were reviewed and approved (IRB201701647) by the Institutional Review Board (IRB-01) at the University of Florida (UF) Gainesville Health Science Center, and the study was registered with ClinicalTrials.gov (NCT03266120) before the start of participant recruitment. All study subjects voluntarily were given and signed the IRB approved written informed consent document in accordance with Declaration of Helsinki and Federal Policy for the Protection of Human Subject regulations (“Common Rule”). This study was carried out in strict accordance with all approved study protocols with no deviations.

The recruitment campaign began approximately two months before the planned start of the art and gardening interventions in October, 2017. Based on power calculations from a pilot gardening study in 2015 [37], sample size estimations for psychometric assessments for the gardening intervention with a healthy population of women ages 26–49 ranged from 15 to 18 participants. There were no preliminary data available to make power calculations for the art intervention for this study population.

A printed flyer distributed throughout the University of Florida (UF) campus and Gainesville, Florida, was the primary recruitment effort. Additional recruitment efforts included community outreach services provided by HealthStreet at the University of Florida (https://healthstreet.program.ufl.edu/) and posting study details on ResearchMatch.org (https://www.researchmatch.org). Other approved recruitment procedures were deemed unnecessary given the number of inquiries received primarily from flyer-generated interest.

### Informed consent and screening

The recruitment campaign generated 101 inquiries. The study coordinator followed an IRB-approved script when contacting interested community members by telephone. The initial phone conversation and pre-screen included a brief description of the study, an inquiry of the community member’s interest and availability for participation, and a preliminary assessment of eligibility requirements. Individuals meeting essential eligibility criteria were invited to schedule an in-person interview with the study coordinator. Forty-four individuals attended the in-person interview. Following consenting, interviewees were asked to fill out two questionnaires, one for eligibility screening and a second for collecting demographic information. Of the forty-four individuals who consented and were screened, two were found to be ineligible for the study based on the inclusion criteria. Two were conditionally eligible depending on which group they may be randomly assigned.

### Randomization

Eligible participants were randomly assigned to one of two groups. The allocation process of participants to the treatment groups was concealed to both the participants and investigators. The study coordinator performed a blinded allocation process using a drawing of lots randomization design. After the random assignment of participants to the two groups, groups 1 and 2. The groups were randomly assigned to an intervention by the blinded drawing of a lot (GG or AG). Lot GG was drawn and group 1 was assigned to the gardening treatment, and group 2 assigned to the art treatment. Two conditionally-eligible participants were assigned to a group in which they were pre-determined to be ineligible, (one in each of the two groups) and were excluded from the study. Therefore, twenty participants each were assigned to the art and gardening interventions, respectively.

Based on each study participant’s availability on Mondays and Wednesdays or Tuesdays and Thursdays for the twice-weekly sessions from information gathered in the eligibility questionnaire, participants were assigned to attend intervention sessions on days that best matched their schedules and availability. An equal number of participants assigned to intervention sessions on Monday and Wednesday and Tuesday and Thursday sessions resulted.

The forty assigned participants were each notified by email from the study coordinator detailing their assigned intervention and inviting them to attend a study orientation session, visit 2, either on Monday or Tuesday, depending on which days they were designated to participate in the study. Participants assigned to the gardening intervention were asked to arrive at the Therapeutic Horticulture Greenhouse Complex at Wilmot Botanical Gardens. Participants assigned to the art intervention were directed to the Conference Center at Wilmot Botanical Gardens. These two buildings are located about 30 meters from each other. Based on the recruitment materials and the nature of the interventions, study participants were not blinded to their respective intervention groups. Study hypotheses were masked to the participants. Study participants assigned to each intervention had no contact or knowledge of participants in the other intervention group.

The art intervention activities were delivered by two Artists in Residence from the UF Health Shands Arts in Medicine Program. The gardening activities intervention was delivered by the study coordinator, a master’s level horticulturist with training in therapeutic horticulture. Physiological monitoring and psychometric assessments for the art and gardening interventions were supervised by the PI and study coordinator, respectively, with direct assistance to participants provided by trained undergraduate student study staff members.

### Orientation and interventions

Participants interacted with study staff on eleven visits. A numbering system was used to label each visitation, with Visit 1 being consenting and eligibility screening. Visit 2 was the orientation to the study and baseline self-report and cardiac data collection. Visits 3 through 10 were the eight group-based art or gardening intervention sessions, and Visit 11 was the follow-up session when the final self-report and cardiac physiological data were collected. Visit 2 began on October 9, 2017, and Visit 11 ended on November 9, 2017 (see **Table 1** for a list of art and gardening sessions with associated visitation numbers). A more detailed description of the intervention activities can be found in the supplementary information (see S1 and S2 Documents). After the interventions were initiated, there were no changes in any methodology or procedure. The one-hour orientation session, visit 2, included a facilities tour, parking instructions, explanation of experimental procedures, and introductions of study participants to the study staff and the other participants in the same intervention group. Baseline psychometric assessments and heart rate (HR) and blood pressure (BP) measurements were collected. The self-report psychometric assessments were always administered in the same order in the assessment packet.

**Table 1:** List of art and gardening intervention sessions and participant visitation numbers.

Study Visit Number^a^	Treatment Session Number	Art Activity	Gardening Activity
3	1	Papermaking	Comparing, Planting Herb Seeds
4	2	Image Transfer	Propagation of Herb & Sensory Plants
5	3	Visual Storytelling	Transplanting Succulent Plants
6	4	Linocut Printmaking: Part 1	Propagation of Ornamental Plants
7	5	Linocut Printmaking: Part 2	Simulated Harvest: Tasting Herbs
8	6	Paper Batik	Seeding Fast Germinating Vegetables
9	7	Mixed Media Collage	Transplanting Herbs & Lettuce
10	8	Sensation Drawing	Simulated Harvest: Tasting Microgreens

^a^Participant visit 1 was initial screening and consenting, and Visits 2 and 11 were pre-intervention and post-intervention evaluations respectively. Activities for the two interventions were selected by the Study Coordinator, PI and Artists in Residence to provide similar levels of learning, use of hands-on fine motor skills, minimal but similar levels of physical activity, individual creativity, and shared group interactions.

After brief introductions and explanation of study protocols, blood pressure monitors were distributed to each subject for initial training and self data collection with assistance by study staff (see "Cardiac Measurements" for data collection procedures). Towards the end of the orientation session, each participant was given the pre-assembled packet of six self-report psychometric assessments. Following assessment packet completion and the second collection of HR and BP measurements. Participants assigned to the art intervention were also given a free copy of *The Artist’s Handbook* by Ray Smith, 3rd edition [38] that provided general background information on the art media and tools used in the art activities. Participants in the gardening intervention were given a free copy of the *Florida Gardener’s Handbook* by MacCubbin and colleagues [39] that provided general background information on the plants and materials used in the gardening activities. The purpose of supplying the handbooks was to provide a complete gardening or art-making and educational experience to complement the sessions’ hands-on activities.

Participants randomized to the art intervention group were asked not to visit art galleries, art museums, or arts and crafts events and not to engage in any art activities outside of the study’s art sessions. Art intervention participants were also asked not to visit art-related websites on the Internet and to confine reading about art and art activities to *The Artist’s Handbook* they received. Participants randomized to the gardening group were asked not to visit parks or botanical gardens and not to engage in gardening activities or interactions with plants outside of the study gardening sessions. Gardening participants were also asked not to visit gardening websites on the Internet and to confine reading about plants, gardens, and gardening to the *Florida Gardener’s Handbook* they received.

The eight twice-weekly art or gardening intervention sessions were administered during Visits 3 through 10. The timeline and sequence of components in each treatment session were maintained throughout the eight sessions, as shown in **Table 2**. During the art and gardening sessions, the principal investigator traveled between the two concurrent sessions to observe and confirm experimental comparability. Following the conclusion of each art and gardening session, the leaders and study staff recorded de-identified notes of informal observations made during the treatment session. The post-session review considered general group interest, engagement, and participation in the activities and recalled unsolicited feedback from unidentified participants such as "I had fun," "I liked planting seeds," or "I liked last week’s art activity better than today’s," as well as whether participants were interactive with other participants in cooperative group activities.

**Table 2:** Timeline sequence for the eight experimental art and gardening intervention sessions.

Timeline	Session Component
0 min	Participants arrival and sign-in
0–10 min	Participants individually review current and previous activities (i.e. art projects or plant growth), session leaders greet participants and assemble group, heart rate and blood pressure measurements recorded
10–20 min	Educational module, session leaders give introduction to activity, demonstration of activity as warranted (with reference to booklet, instruction sheet, and/or any resource material handouts)
20–50 min	Art or gardening activity, questions and dialog with session leader(s), communication and interactions with other participants in the session
50–60 min	Clean-up, complete self-report Profile of Mood States and Perceived Stress Scale assessments (on even-numbered sessions) and Beck Depression Inventory-II (sessions 4 and 8), and heart rate and blood pressure measurement recorded for all sessions, participant sign out and departure
60–85 min	Session leaders and study staff conduct session review, evaluation, and record observations (study staff only)

### Art-making intervention

The art sessions took place in the Wilmot Botanical Gardens conference center, either on Mondays and Wednesdays or Tuesdays and Thursdays at 5:30 pm DST (visits 2–9) or 5:30 pm EST (visits 10 and 11) at the same time of day as the gardening sessions (**Table 1**). The 1032 sq. ft. conference center with tables and chairs is an air-conditioned indoor space that comfortably met the study participants’ needs.

Each art activity was designed and administered by two professional artists in residence from the University of Florida Arts in Medicine program. All the individual art sessions’ activities were planned to involve approximately a similar level of physical, cognitive, and social engagement as the gardening intervention activities to minimize differential competing interacting effects in these aspect areas. Participants received a guide booklet containing details of the eight sessions that provided instructions and information for each art activity (see Art Intervention Handbook/Manual S1 Document). The Art Intervention booklet provided a detailed description of the educational, social, and physical purposes, goals, and benefits of the eight art sessions.

### Gardening intervention

The gardening sessions took place inside the 2700 sq. ft. therapeutic horticulture greenhouse at Wilmot Botanical Gardens on Mondays and Wednesdays or Tuesdays and Thursdays (**Table 1**). The gardening sessions were conducted inside the greenhouse to increase control and uniformity of environmental conditions compared to conducting outdoor sessions (see S1 Fig). Ambient conditions for temperature and humidity in the greenhouse were recorded at the beginning and end of each gardening session to provide information about the environmental conditions throughout the intervention. Outside weather conditions during the gardening sessions were also recorded. Temperature and humidity conditions were used to calculate the heat index [40] during the sessions (http://www.wpc.ncep.noaa.gov/html/heatindex.shtml). An evaporative cooling system, automated, adjustable shade curtains, and a double roof vent system were used to keep the greenhouse in a comfortable temperature range for participants.

The experimental intervention was configured to introduce and reinforce a limited number of different gardening and horticultural themes that included: propagation presented in two forms including planting seeds (2 sessions) and vegetative propagation by cuttings/divisions (2 sessions); transplanting (2 sessions); and simulated harvest (2 sessions). The emphasis and reinforcement of the selected gardening themes were achieved using different types of plants with different uses and characteristics in the various sessions. The activities for all individual sessions were designed to involve a similar level of physical, cognitive and social engagement to filter out differential, competing, or interacting effects in these aspect areas. Participants received a guide booklet containing details of the eight sessions that provided instructions and information for each of the gardening activities (see Gardening Intervention Handbook/Manual S2 Document). The booklet provided a detailed description of the educational, social and physical purposes, and goals and benefits associated with the eight gardening sessions. A registered, masters-level horticultural therapist (HTM) reviewed each gardening activity and the overall experimental treatment intervention during experimental design development. A horticulture master’s student and study coordinator led each gardening activity session with the help of trained study staff.

### Self-reported health assessment evaluations

The Profile of Mood States 2-A (POMS) short form [41], Perceived Stress Scale (PSS) [42], Beck Depression Inventory II (BDI-II) [43], State-Trait Anxiety Inventory for Adults (STAI) [44], PROMIS Satisfaction with Participation in Discretionary Social Activities (SPDSA) [45], and SF-36v2 (SF-36) [46] instruments were administered before the beginning and following the completion of the art and gardening treatment regimens during Visits 2 and 11. The approximate time to complete all six self-report questionnaires was 35 minutes. Also, the POMS short form and PSS instruments were administered every week at the end of the 2nd, 4th, 6th, and 8th art and gardening sessions during Visits 4, 6, 8, and 10 to provide a time-course of mood and stress status, respectively, during the experiment. The BDI-II was administered at the end of the 4th and 8th art and gardening sessions during Visits 6 and 10 to provide information on the depression symptomatology status during the course of the treatment interventions.

When participants finished their self-report assessments, the study staff checked the assessments to ensure all questions were answered. When the BDI-II or STAI were administered, the study staff scored and reviewed questions on the BDI-II and STAI before the participants departed the session to ensure answers did not indicate suicidal ideation, severe depression, or anxiety symptomatology. More specifically, if the raw score of the BDI-II was above 30 or responses of 1, 2, or 3 on question 9 were identified for any subject, a trained counselor would be called immediately. Similarly, if the responses to questions 25, 29, 31, or 38 on the STAI indicated elevated levels of anxiety symptoms, then a trained counselor would be called for professional guidance concerning steps to take to ensure the well-being of the study subject. During data collection, no incidental findings for the BDI-II or STAI for any participant were observed.

### Blood pressure and heart rate measurements

Minimally invasive Omron BP652N wrist cuff blood pressure and heart rate monitors were used given the device’s small structure and measurement placement [47]. The instruction manual for the Omron Wrist Blood Pressure Monitor Model BP652N (HEM-6300-Z) reported blood pressure (BP) accuracy within 2 percent of the readings and heart rate (HR) accuracy within 5 percent of the reading. In an instrument validation study conducted by Takahashi and colleagues [48], Omron RS3 (HEM-6130-E) wrist BP monitors, the European equivalent to the Omron BP652N, met the 2007 validation standards of the European Society of Hypertension, meaning that differences between BP result from this Omron device and the standard mercury sphygmomanometer were within 3 mm Hg. These findings also fulfill the criteria set forth by the American Association for the Advancement of Medical Instrumentation, where the mean difference between the BP device and the standard mercury sphygmomanometer must be less than 5 mm of Hg.

HR and BP readings were acquired in triplicate for all participants at the beginning and end of visits 2 through 11, i.e., the orientation session, eight art and gardening intervention sessions, and the follow-up post-intervention session. The study staff reviewed HR and BP readings before participants left to ensure no readings were above 180 mm Hg for systolic BP, 110 mm Hg for diastolic BP, or 100 beats per minute (BPM) for HR. No incidental findings were observed for HR or BP.

### Participant completion of study

**Fig 1:**
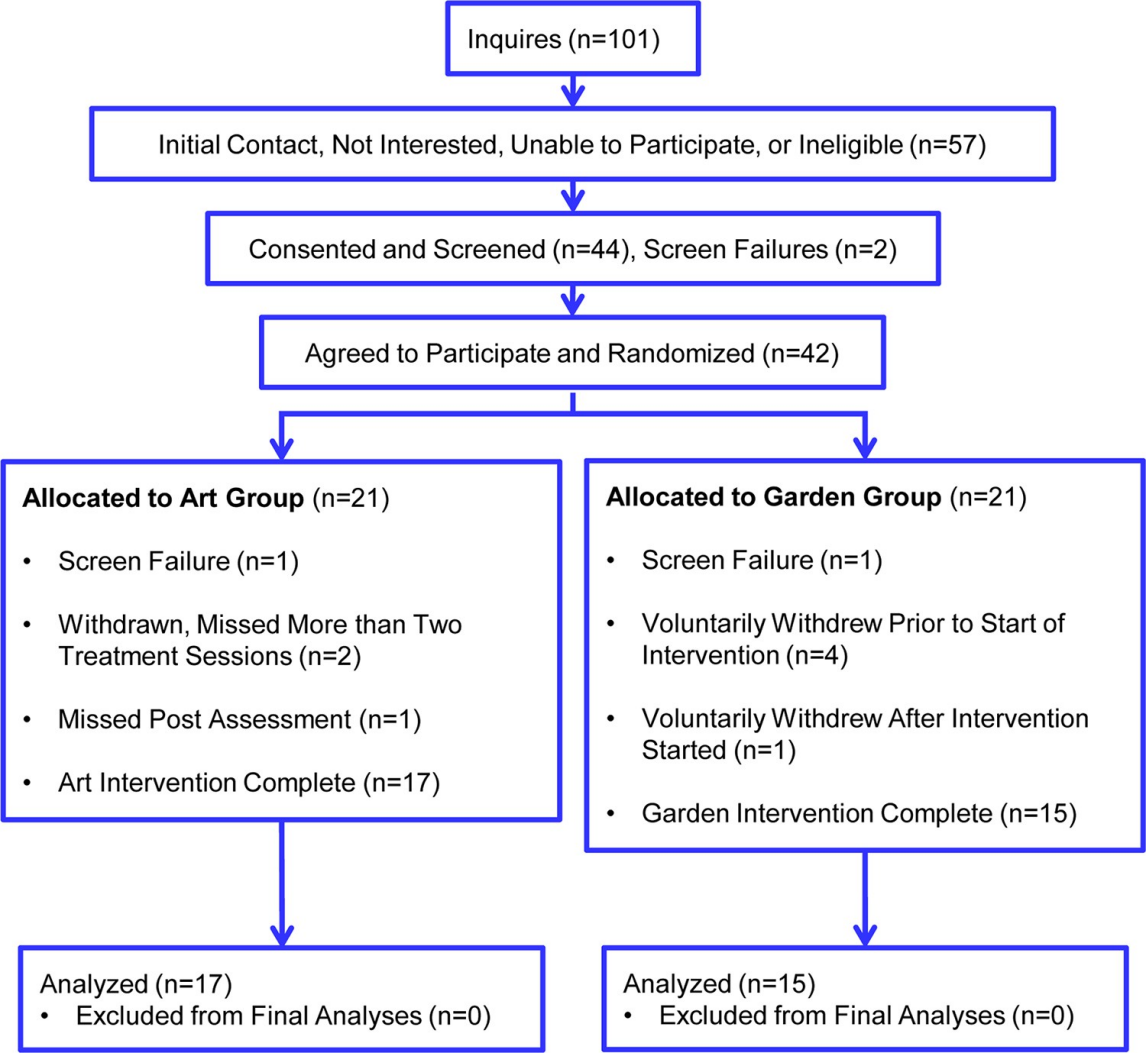
CONSORT (Consolidated Standards of Reporting Trials) recruitment, consenting, enrollment, screening and intervention completion diagram for the gardening and art randomized controlled trial.

**Fig 1** shows the participant flow diagram. A total of 42 volunteers were randomized to the two treatment groups, with two becoming screening failures based on random group assignment. Following study participant notification of their group assignment and start date, four participants voluntarily withdrew from the study. They indicated that they were no longer able to participate. All four participants had been assigned to the Monday and Wednesday gardening intervention sessions. One additional participant withdrew from participation after completion of the second gardening session due to family commitments. Based on dosage considerations, the experimental design was pre-determined for participants to complete at least 75% of the respective treatments for inclusion in pre-post data analyses. Upon missing a third art or gardening intervention session, study participants were withdrawn from further participation in the study by the principal investigators. Two participants were withdrawn from the art intervention group for missing three art sessions. One additional subject in the art intervention did not attend the post-intervention session, nor complete final assessments. Missing data were not imputed or included in any data analyses. Outcomes were analyzed following the Per-Protocol approach.

### Data analytics/Statistical analysis

Undergraduate student study staff members entered participant coded data from the six paper-administered psychometric assessments into an Excel database. Data entry was performed in pairs with one individual reading items from each assessment, while a second individual entered the data into a master spreadsheet using Excel. This same data entry procedure was used to enter POMS data into the Multi-Health Systems Online Assessment Center, which calculated the Total Mood Disturbance (TMD) and seven subscale scores based on item responses. In the same way, data was entered for the SF-36v2 in a template spreadsheet and submitted into the Optum® PRO CoRE software for processing the mental and physical component and subscale calculations for the SF-36. PSS (items 4, 5, 7, 8) and STAI (STAI-State items: 1, 2, 5, 8, 10, 11, 15, 16, 19, 20; STAI-Trait items: 21, 23, 26, 27, 30, 33, 34, 36, 39) required reverse scoring for final calculations.

Standard univariate and multivariate tests were conducted to evaluate statistical separations between art and gardening group scores and baseline scores to post-intervention follow-up scores. Effect size estimations for each of the six self-report assessments were determined using Cohen’s *d* statistic [49], reflecting magnitudes of change within the art and gardening groups.

Before statistical testing, psychometric score data were evaluated for normality, homogeneity of variance, and sphericity by histogram inspection, Levene’s Test, and Mauchly’s Test, respectively. A two-way (intervention group X time) repeated measures analysis of variance (RM-ANOVA) with main effects decomposition was used to statistically compare differences in baseline scores, changes from baseline to post-intervention scores, and differences in post-intervention scores between experimental groups for the six self-report psychometric assessments. Mean differences were considered significant at *p* < .05 after adjustments for multiple comparisons with the Bonferroni correction. The IBM SPSS Statistics package version 25 was used to conduct univariate statistics.

Generalized mixed linear models controlling for age were conducted to analyze the systolic and diastolic BP and heart rate data. SAS version 9.4 was used to run these multivariate tests and calculate fit models. Additional mixed models were evaluated to compare the differences within the eight intervention sessions and ten total sessions (including the pre-intervention and post-intervention sessions) for the art and gardening interventions separately to determine whether BP and HR intra-session changes were significantly different from 0 (no change) at *p* < .05.

## Results

**Fig 2:**
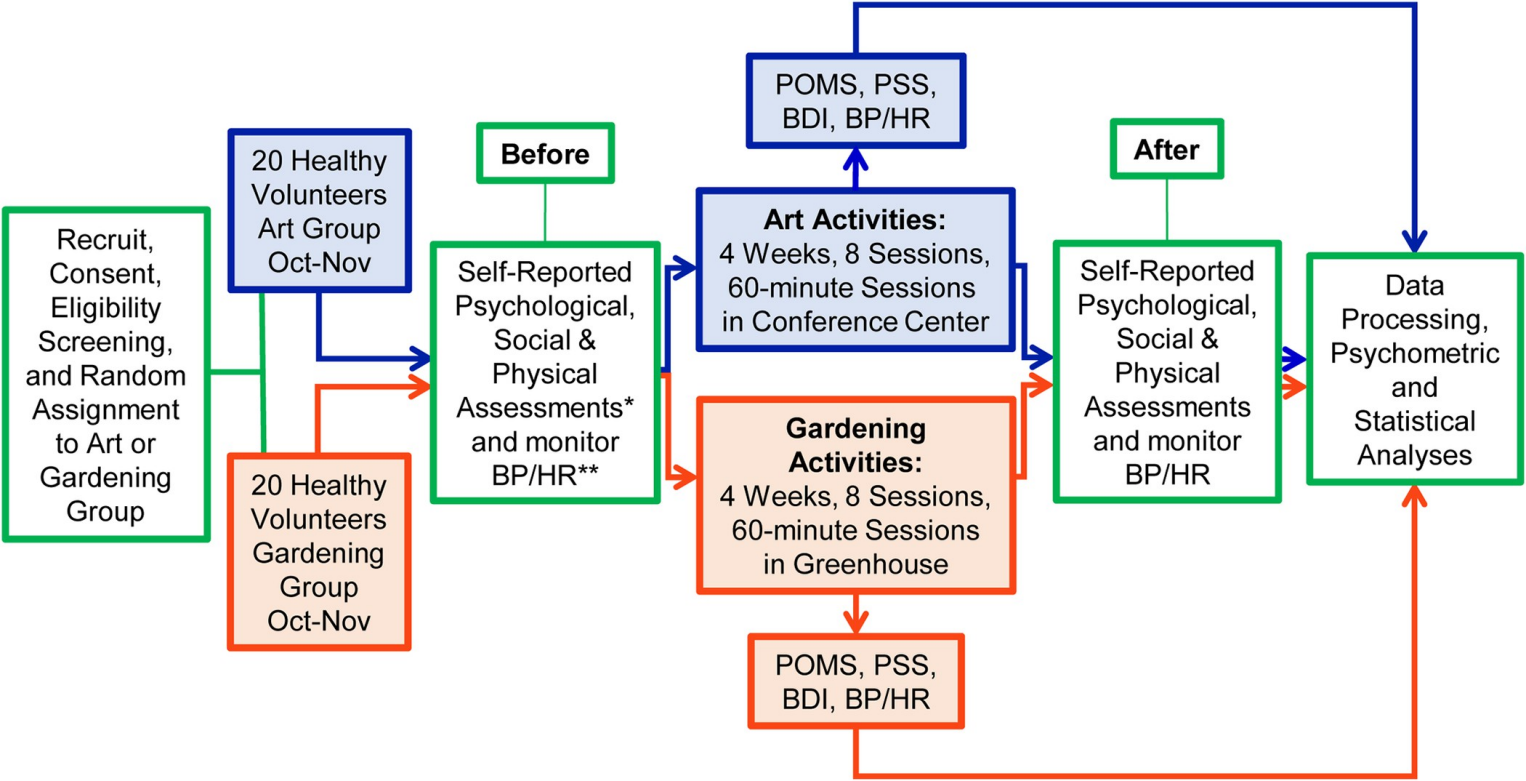
Study experimental design. Self-report psychometric assessments* included the Profile of Mood States 2nd Ed. Adult Form (POMS); Perceived Stress Scale (PSS); Beck Depression Inventory 2nd Ed. (BDI-II); State-Trait Anxiety Inventory Adult Form (STAI); Satisfaction with Participation in Discretionary Social Activities (SPDSA) PROMIS Short Form v1.0; and SF-36v2 (SF-36) Health Survey 3rd Ed. Blood Pressure** (BP) and Heart Rate** (HR) were collected using Omron 7 Series Wrist Blood Pressure Monitors (BP652N).

This study evaluated the psychological, social, and physiological effects in a two-arm parallel group-based art-making intervention as an active, concurrent control for a group-based indoor gardening intervention within a randomized and controlled true experimental design (**Fig 2**). Engaging in a group-based gardening treatment is a complex intervention [50]. Given the complexity of the gardening intervention, a non-concurrent no-treatment intervention was deemed not to be an equivalent and suitable control. Therefore, a group-based art-making intervention was selected to serve as a comparable non-gardening equivalent that possessed six of the seven key characteristics of the gardening intervention. The art-making activities were devoid of anything that was plant related.

### Treatment group participant demographics

The demographic, lifestyle, and health characteristics for the art and gardening treatment groups are shown in **Table 3**. Random assignment of study participants resulted in two fundamentally equivalent art and gardening intervention groups to begin the experiment for age, socioeconomic details, and other demographic and health factors. Participant average age was approximately 32–33 years; was well-educated, and had an average household income above (art group) and below (gardening group) the median income for Alachua County, Florida. Upon entering the study, participants cared, on average, for approximately two plants, consumed low amounts of alcoholic beverages, did not smoke and had Body Mass Index (BMI) scores [51], heart rate, and blood pressures that were in the normal range. Participants self-rated their current physical and mental health as 7.8/7.6 and 8.3/8.1 for the art/gardening groups, respectively, on a scale of 1 to 10, with 10 being perfect health. The gardening group had fewer children at home than the art group. The groups’ racial makeup was 2.5% American Indian/Alaskan Native, 5% Black, 17.5% other race, 25% Asian, and 50% White. Of these racial classifications, 27.5% identified as having a Hispanic or Latino ethnic identity. This study’s racial and ethnic profile is similar to the Florida population profile except for having a smaller proportion of Blacks and a greater proportion of Asians (US Census 2017; https://factfinder.census.gov/faces/tableservices/jsf/pages/productview.xhtml?src=bkmk).

**Table 3:** Baseline characteristics for the randomly assigned art and gardening groups.

Category	Intervention	N	Mean ± SD
Age	Art	20	32.8 ± 5.6
	Gardening	16	32.1 ± 5.1
Years of Education^a^	Art	20	18.4 ± 3.5
	Gardening	16	18.2 ± 1.9
Annual Family Income ($1,000 USD)	Art	19^b^	55.5 ± 41.0
	Gardening	15^c^	41.4 ± 25.5
Marital Status (Married/Single)	Art	8/12^c^	NA^d^
	Gardening	7/9^c^	NA^d^
Number of Children at Home	Art	20	0.6 ± 1.1
	Gardening	16	0.1 ± 0.3
Number of Plants Cared for at Home	Art	20	1.6 ± 2.7
	Gardening	16	2.6 ± 2.2
Number Alcoholic Drinks in Average Week	Art	20	1.8 ± 1.6
	Gardening	16	0.9 ± 1.4
Number of Cigarettes Smoked in Average Week	Art	20	0.0 ± 0.0
	Gardening	16	0.0 ± 0.0
Frequency of Exercise in Average Week	Art	19	3.1 ± 1.7
	Gardening	16	2.7 ± 1.3
Body Mass Index (BMI)	Art	20	23.4 ± 3.2
	Gardening	16	23.4 ± 3.2
Heart Rate (Beats per Minute)	Art	20	73.2 ± 10.4
	Gardening	16	72.5 ± 9.0
Systolic Blood Pressure (mm Hg)	Art	20	114.2 ± 13.6
	Gardening	16	111.0 ± 12.5
Diastolic Blood Pressure (mm Hg)	Art	20	70.6 ± 10.1
	Gardening	16	69.2 ± 8.5
Self-Rated Physical Health (1–10 Scale)	Art	20	7.8 ± 1.1
	Gardening	16	7.6 ± 1.4
Self-Rated Mental Health (1–10 Scale)	Art	20	8.3 ± 1.2
	Gardening	16	8.2 ± 1.3

^a^Grades 1 through 12 account for 12 years of education.

^b^N = 19 and 15; missing data for one subject in each group.

^c^N = 20 and 16 for each group; indicates number married/number single.

^d^NA, not applicable.

### ANOVA (significance/hypothesis testing and treatment effects)

Assumptions associated with parametric testing were assessed for this data set, including normality and homogeneity of variance. **Table 4** provides descriptive statistics related to within and between intervention groups differences pre- to post-intervention for the POMS, PSS, BDI-II, STAI, SF-36, and SPDSA psychometric instruments based on two-way group by time repeated measures ANOVA (RM-ANOVA) pairwise comparisons. The *F*-Test table clearly shows no significant differential Group effects and no Time*Group effects. This statistical outcome addresses hypothesis 1 of the study of no overall statistical difference between the art and gardening treatments. However, there were By Time differences for four of the assessments suggesting there were treatment dosage response outcomes for both groups.

**Table 4:** *F*-Test table from a two-way repeated measures ANOVA with Bonferroni correction for comparisons within each psychometric instrument assessment.

Assessment Instrument	*F* Statistic	*p*-value
**POMS TMD**		
Time	**14.07**	**.001**
Group	0.01	.933
Time*Group	0.01	.921
**PSS**		
Time	**25.27**	**< .001**
Group	0.16	.692
Time*Group	0.02	.899
**BDI-II**		
Time	**20.65**	**< .001**
Group	0.70	.411
Time*Group	0.55	.463
**STAI-State**		
Time	2.04	.164
Group	0.02	.895
Time*Group	2.14	.154
**STAI-Trait**		
Time	2.94	.097
Group	0.69	.412
Time*Group	1.85	.184
**SF-36 Physical**		
Time	1.15	.293
Group	1.76	.195
Time*Group	0.15	.706
**SF-36 Mental**		
Time	1.90	.178
Group	0.05	.832
Time*Group	0.13	.721
**SPDSA**		
Time	**4.51**	**.042**
Group	0.04	.851
Time*Group	0.00	1.000

Note: Time reflects pre-intervention and post-intervention means. Group reflects assignment to art or gardening treatment group. Bold font indicates two-tailed *p* < .05.

Overall, psychometric assessments at baseline demonstrate how comparable the two treatment groups were, with the largest statistical difference being the STAI scores for Trait anxiety (**Table 5**), with the gardening group score being higher than the art group by 3.6 units. The pre-intervention scores for POMS TMD, PSS, BDI-II, STAI-State, and STAI-Trait for the art group were all within normative ranges (see S2 Table) as would be expected for a healthy population. The POMS TMD, PSS, BDI-II, and STAI were also within normative ranges for the gardening group, but STAI-Trait was just above the normative range.

**Table 5:** Art and gardening groups self-report psychometric assessment statistics. Values computed from time by intervention group repeated measures pair-wise *t*-tests.

Assessment Instrument^a^	Intervention	N^b^	Pre-Intervention Mean ± SD^c^	Post-Intervention Mean ± SD^d^	Pre- to Post- Intervention *p*-value^e^
POMS TMD (T-scores)	Art	17	**53.5 ± 9.6**	**47.0 ± 10.0**	**.009**
	Gardening	15	**53.1 ± 9.0**	**46.9 ± 7.4**	**.018**
PSS	Art	17	**15.8 ± 7.2**	**10.0 ± 6.8**	**.001**
	Gardening	15	**14.9 ± 4.1**	**9.4 ± 5.6**	**.002**
BDI-II	Art	17	**9.0 ± 6.3**	**5.1 ± 6.6**	**.009**
	Gardening	15	**8.2 ± 6.8**	**2.8 ± 3.6**	**.001**
STAI-State	Art	17	32.1 ± 9.3	32.2 ± 7.8	.981
	Gardening	15	*34*.*3 ± 11*.*1*	*29*.*3 ± 6*.*6*	.*057*
STAI-Trait	Art	17	36.4 ± 9.8	35.9 ± 9.1	.797
	Gardening	15	**41.1 ± 12.4**	**37.0 ± 10.2**	**.044**
SF-36 Physical Health (Norm-based scores)	Art	16	57.7 ± 4.0	58.1 ± 3.1	.624
	Gardening	15	55.3 ± 5.8	56.2 ± 5.9	.320
SF-36 Mental Health (Norm-based scores)	Art	16	45.5 ± 11.3	47.3 ± 8.9	.470
	Gardening	15	45.5 ± 10.6	48.6 ± 6.5	.236
SPDSA (T-scores)	Art	17	49.8 ± 7.6	52.3 ± 9.1	.131
	Gardening	15	49.7 ± 6.4	51.6 ± 4.4	.156

^a^POMS TMD = Profile of Mood States 2^nd^ Ed. Adult Form Total Mood Disturbance; PSS = Perceived Stress Scale; BDI-II = Beck Depression Inventory 2^nd^ Ed.; STAI-State = State-Trait Anxiety Inventory State Subscale; STAI-Trait = State-Trait Anxiety Inventory Trait Subscale; SF-36 Physical and Mental Health = Components of SF-36v2 Health Survey 3^rd^ Ed.; SPDSA = Satisfaction with Participation in Discretionary Social Activities PROMIS Short Form v1.0.

^b^Number of participants completing all study assessments.

^c^All pre-intervention mean pairwise comparisons between art and gardening intervention groups were not significantly different for a two-tailed test at *p* < .05.

^d^All post-intervention mean pairwise comparisons between art and gardening intervention groups were not significantly different for a two-tailed test at *p* < .05. ^e^Bold font indicates pre- to post-intervention within group means with uncorrected two-tailed test at *p* < .05. Bonferroni correction for multiple comparisons within each psychometric assessment (m = 4) for an overall *p*-value = .05 would result in only POMS art, PSS art and gardening, and BDI-II art and gardening meeting a corrected *p*-value < .0125.

Group means for SF-36 Physical Health were above the normative range for both art and gardening groups, while the scores for SF-36 Mental Health for both groups were just slightly below the normative range. Perhaps the higher Physical Health and the lower Mental Health scores on the SF-36 assessment reflect that the treatment groups were composed of healthy women only since the norm-based scores were derived from a general population. Normative scores for healthy adults are not available for the SPDSA assessment instrument. When coupled with the screening inclusion and exclusion criteria, the self-report assessments show that the study population consisted of physically and mentally healthy women.

Statistically significant reductions in the POMS TMD scores, PSS, and BDI-II were observed in pre- to post-treatment comparisons for both the art and gardening groups (**Table 5**). A reduction on the STAI-Trait subscale was observed for the gardening group, while no such statistical separation was found for the art group pre- to post-intervention. Similarly, the STAI-State Anxiety subscale score decreased for the gardening group yielding a *p*-value of *p* = .057 when adjusted for multiple comparisons that was just above the *p* = .05 for accepting differences between group means. However, the art group showed no statistically significant changes for the STAI-State Anxiety subscale. Changes in pre- to post-intervention scores for both groups for the physical or mental components of SF-36 and the SPDSA were not statistically significant. Although there were changes in pre- to post-intervention scores for both groups, no pairwise comparisons between the gardening and art group scores were statistically separated at post-intervention assessment.

### Treatment effect size

**Table 6** shows the relative percent change in the pre- and post-scores and the respective calculated effect size values with 95% confidence intervals. Cohen’s *d* statistic [49] was used to calculate the treatment effect size for the art and gardening groups for mean score changes for the six psychometric assessments. A range of treatment effect sizes was observed with both the art and gardening intervention groups based on the magnitude of mean scores from pre- to post-intervention for all psychometric assessments. Generally, larger treatment effect sizes were observed, based on Cohen’s *d* statistic, for the gardening group compared to the art group for the POMS TMD, PSS, BDI-II, STAI-State, and -Trait Anxiety subscales, SF-36 Mental and Physical Health subscales. For the SPDSA assessment, Cohen’s *d* statistic for the art group was larger than that for the gardening group. The effect size categories as given by Cohen can be found in the table footnote [49].

**Table 6:** Treatment effect size calculations using Cohen’s *d*_RM_ for repeated measures.

Assessment Instrument	Intervention	Percent Change Pre- to Post-Intervention^a^	Cohen’s *d*_RM_ Effect Size	Cohen’s *d*_RM_ Effect Size 95% CI
POMS TMD (T-scores)	Art	-12	**-0.60**^**b**^^,^ ^**c**^^,^ ^**d**^	**-1.30 –-0.07** ^*^
	Gardening	-12	**-0.81**	**-1.48 –-0.01** ^*^
PSS	Art	-37	**-0.85**	**-1.52 –-0.12** ^*^
	Gardening	-37	**-0.97**	**-1.94 –-0.39** ^*^
BDI-II	Art	-43	**-0.74**	**-1.46 –-0.06** ^*^
	Gardening	-66	**-0.90**	**-1.46–0.02**
STAI-State	Art	0.3	-0.01	-0.66–0.68
	Gardening	*-15*	*-0*.*45*	-1.09–0.36
STAI-Trait	Art	-1	-0.08	-0.75–0.59
	Gardening	-10	**-0.47**	-1.16–0.29
SF-36 Physical (Norm-based scores)	Art	1	0.13	-0.55–0.79
	Gardening	2	0.24	-0.48–0.92
SF-36 Mental (Norm-based scores)	Art	4	0.19	-0.51–0.84
	Gardening	7	0.32	-0.45–0.99
SPDSA (T-Scores)	Art	5	0.58	-0.08–1.35
	Gardening	4	0.29	-0.49–1.00

^a^Percent change calculated using Pre and Post values in Table 5 $(\frac{\mathrm{Post}-\mathrm{Pre}}{\mathrm{Pre}})\;x\;100$.

^b^Effect Size calculated using pooled SDs from pre- and post-intervention mean scores.

^c^Effect Size values are bold if uncorrected *p*-values from Table 5 pre- post-comparisons were *p* < .05.

^d^Cohen’s Effect Size *d* categories: small >0.20, <0.50; medium >0.50, <0.80; large >0.80 [49].

^*^Effect size 95% Confidence Interval does not cross 0.00.

The POMS subscales pre- to post-intervention comparisons are shown in **Tables 7**–**9**. The *F*-Test table clearly shows a Time, but no Group or Time*Group interaction effect within each of the POMS subscale assessments. The repeated measures ANOVA indicates treatment by time effects for Anger, Confusion, Depression, Fatigue, and Tension, but no differences for Vigor and Friendliness.

**Table 7:** POMS subscales *F*-Test table from a two-way repeated measures ANOVA with Bonferroni correction for comparisons within each subscale assessment.

POMS Subscale	*F* Statistic	*p*-value
**Anger**		
Time	**7.495**	**.010**
Group	3.258	.081
Time*Group	0.582	.451
**Confusion**		
Time	**18.782**	**< .001**
Group	0.065	.800
Time*Group	1.482	.233
**Depression**		
Time	**5.690**	**.024**
Group	0.028	.867
Time*Group	0.000	.999
**Fatigue**		
Time	**13.057**	**.001**
Group	0.352	.557
Time*Group	0.446	.509
**Tension**		
Time	**14.568**	**.001**
Group	0.283	.598
Time*Group	0.885	.354
**Vigor**		
Time	0.413	.525
Group	0.003	.959
Time*Group	1.265	.270
**Friendliness**		
Time	0.159	.693
Group	0.261	.613
Time*Group	0.000	.994

Note: Time reflects pre-intervention and post-intervention means. Group reflects assignment to art or gardening treatment group. Bold font indicates two-tailed *p* < .05.

**Table 8:** POMS subscale statistics for the art and gardening interventions.

POMS Subscale	Intervention	N	Pre-Intervention Mean ± SD^a^	Post-Intervention Mean ± SD^b^	Pre- to Post- Intervention *p*-value^c^
Anger	Art	17	49.5 ± 5.1	47.2 ± 9.2	.160
	Gardening	15	**46.9 ± 5.6**	**42.8 ± 4.6**	**.023**
Confusion	Art	17	**51.5 ± 10.4**	**45.2 ± 7.2**	**< .001**
	Gardening	15	**49.4 ± 7.7**	**45.9** ± **7.2**	**.041**
Depression	Art	17	50.4 ± 7.8	46.9 ± 7.2	.092
	Gardening	15	50.1 ± 7.8	46.6 ± 5.0	.112
Fatigue	Art	17	**47.4 ± 7.3**	**42.4 ± 8.9**	**.040**
	Gardening	15	**49.8 ± 7.6**	**42.5 ± 6.5**	**.006**
Tension	Art	17	**50.2 ± 10.0**	**45.8 ± 9.8**	**.044**
	Gardening	15	**53.1 ± 10.3**	**45.9 ± 5.8**	**.003**
Vigor	Art	17	49.0 ± 10.1	52.2 ± 10.7	.207
	Gardening	15	50.9 ± 8.8	50.0 ± 9.7	.743
Friendliness	Art	17	48.1 ± 9.5	48.8 ± 10.1	.777
	Gardening	15	49.6 ± 8.9	50.3 ± 10.2	.782

^a^All pre-intervention mean pairwise comparisons between art and gardening intervention groups were not significantly different for a two-tailed *p* < .05.

^b^All post-intervention mean pairwise comparisons between art and gardening intervention groups were not significantly different for a two-tailed *p* < .05.

^c^Bold font indicates pre- to post-intervention within group means with uncorrected *p* < .05. Bonferroni correction for multiple comparisons within each subscale assessment (m = 4) for an overall *p*-value = .05 would result in art for Confusion, and for gardening Fatigue and Tension meeting a corrected *p*-value < .0125.

**Table 9:** Treatment effect size calculations for POMS subscales using Cohen’s *d*_RM_ for repeated measures.

POMS Subscale	Intervention	Percent Change Pre- to Post-Intervention^a^	Cohen’s *d*_RM_ Effect Size	Cohen’s *d*_RM_ Effect Size, 95% CI
Anger	Art	-5	-0.35^b^^,^ ^c^^,^ ^d^	-1.18–0.17
	Gardening	**-9**	**-0.73**	**-1.40–0.07**
Confusion	Art	**-12**	**-0.90**	-1.47 –-0.08^*^
	Gardening	**-7**	**-0.71**	-**1.43–0.04**
Depression	Art	-7	-0.42	-1.08–0.27
	Gardening	-7	-0.45	-1.00–0.34
Fatigue	Art	**-11**	**-0.48**	-1.22–0.15
	Gardening	**-15**	**-0.85**	**-1.54 –-0.05** ^*^
Tension	Art	**-9**	**-0.46**	-1.14–0.22
	Gardening	**-14**	**-1.14**	**-1.68 –-0.17** ^*^
Vigor	Art	7	0.29	-0.38–0.97
	Gardening	-2	-0.10	-0.82–0.61
Friendliness	Art	1	0.06	-0.61–0.74
	Gardening	1	0.08	-0.63–0.80

^a^Percent change calculated using Pre and Post values in Table 8 $(\frac{\mathrm{Post}-\mathrm{Pre}}{\mathrm{Pre}})\;x\;100$.

^b^Effect Size calculated using pooled SDs from Pre- and Post-intervention mean scores.

^c^Effect Size values are bold if uncorrected *p*-value from Table 8 pre- post-comparisons were *p* < .05.

^d^Cohen’s Effect Size *d* categories: small >0.20, <0.50; medium >0.50, <0.80; large >0.80.

^*^Effect size 95% CI does not cross 0.00.

Both art and gardening groups showed improved mood shifts related to confusion, fatigue, and tension; however, only the gardening group also showed improvement in the anger subscale (**Table 8**). No statistically significant changes were found for either treatment group for the depression subscale or two positive mood subscales, vigor and friendliness. These results provide further overall support to the treatments’ therapeutic outcomes that reflect and contribute to total mood disturbance improvements.

Effect size calculations for POMS subscales show a range of treatment effects and suggest that art may have resulted in meaningful improvements for confusion. In contrast, gardening may have resulted in improvements in fatigue and tension (**Table 9**). There is also some statistical support for gardening improvement for anger and confusion.

### Dosage effects on art and gardening psychometric scores

**Fig 3:**
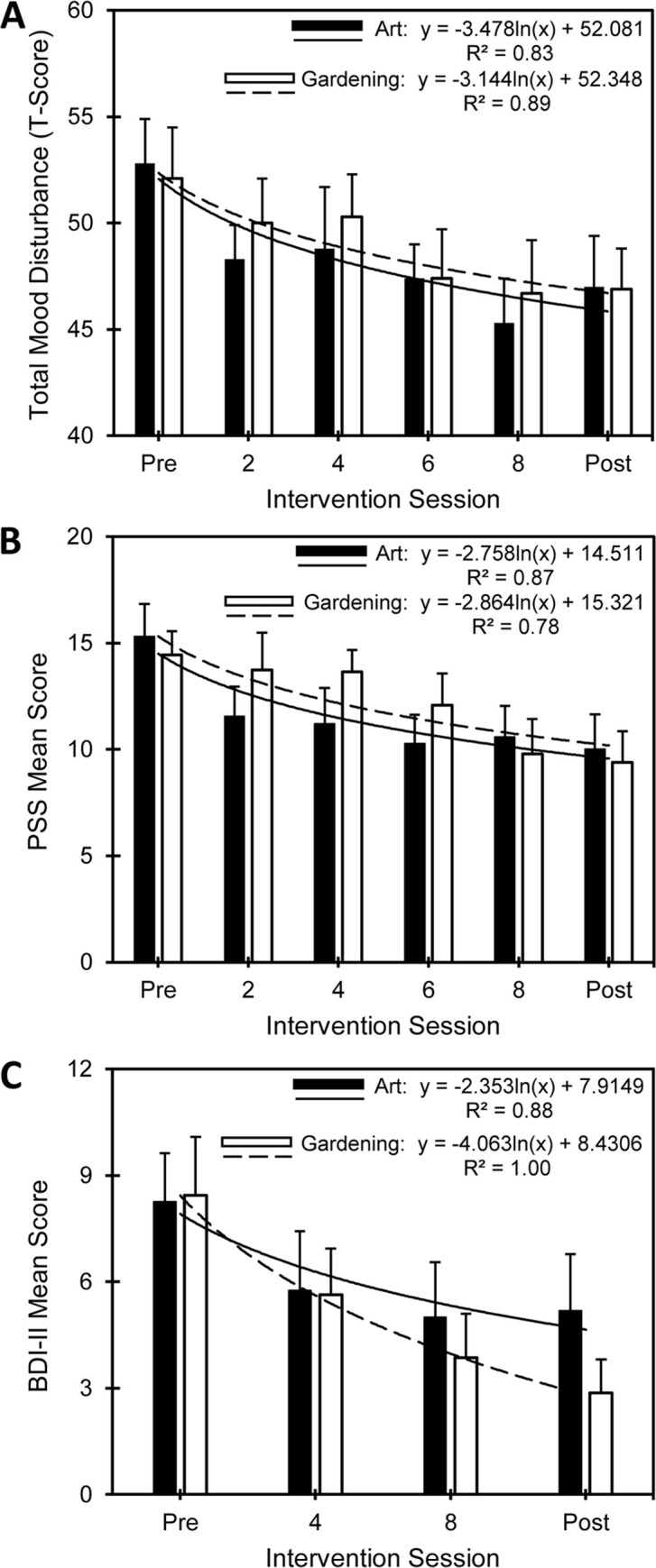
Time course dosage responses. (A) POMS Total Mood Disturbance. (B) PSS. (C) BDI-II. Logarithmic regression trends with respect to art or gardening interventions. Sample size varies between sessions and between groups due to missing data. Error bars represent standard error. Best-fit trend lines and equations were determined using Excel.

Three assessments, the POMS, PSS, and BDI-II, were completed by participants during the interventions at pre-selected, defined intervals. The POMS and PSS were given weekly, while the BDI-II was given every two weeks, as the assessments have a one-week and two-week recall interval, respectively. **Fig 3** shows the changes in scores for the POMS, PSS and BDI-II assessments and regression analyses with the occasion of measurement as a predictor for group score.

**Fig 3A** shows the association between the weekly measures of the POMS for the art and gardening intervention groups. For the art intervention group, 83% of the reduction in the POMS TMD scores can be modeled by a logarithmic function for the progression of the intervention. For the gardening intervention group, 89% of the changes in POMS TMD scores can be modeled by a similar log function for the occasion of measurement and progression of the intervention. **Fig 3B** details the association between the weekly measurements for the PSS for the art and gardening intervention groups. A logarithmic function can model the art intervention group’s 87% changes in PSS scores for the occasion of measurement and progression of the intervention. For the gardening intervention group, 78% of the reductions in PSS scores can also be described by a logarithmic function for the occasion of measurement and progression of the intervention. **Fig 3C** details the association between the repeated measurements of the BDI-II for the art and gardening intervention groups. For the art intervention group, 88% of the decreases in BDI-II scores can also be modeled by a logarithmic function for the progression of the intervention based on responses on the BDI-II. For the gardening intervention group, 99.9% of the decreases in BDI-II scores can be described by a logarithmic function for the occasion of measurement and progression of the intervention.

The gardening group mean score of 2.8 for the BDI-II approaches the floor of the scale, zero. This raises the question if the group mean did not approach the lower limit of the assessment’s scale, might a more substantial treatment outcome been achieved? Endorsements on none of the other instruments demonstrated this pattern. When taken together, the results in **Fig 3** provide clear evidence for dosage effects on scores for self-report psychometric assessment outcomes for POMS TMD, PSS, and BDI-II for both art and gardening treatments. It is intriguing to speculate whether the treatment outcomes could have been more significant had treatment dosages been greater (i.e., more treatment sessions).

### Heart rate and blood pressure

Group baseline average resting heart rates at the pre-intervention orientation for art were 73 and 70 BPM at the beginning and end of the session. At the same time, the values for gardening were 73 and 71 BPM, respectively. For the eight treatment sessions, the average beginning and end HR readings for the art group were 75 and 73 BPM, and for the gardening group, 76 and 76 BPM, respectively. Throughout the treatments, the average beginning and end-systolic BPs for the art group were 119 and 120 mm Hg, respectively, while the averages for the gardening group were 111 and 110 mm Hg. There was a smaller disparity between the diastolic BPs between the art and gardening treatment groups. The beginning and end means for art were 72 and 74 mm Hg, respectively, and for gardening, 69 and 68 mm Hg. The group means for systolic and diastolic blood pressure suggests the absence of hypertension and falls into the normal range. The reason for the disparity in systolic BPs between the art and gardening groups is unknown. However, two possibilities may be: different operators taking the readings or perhaps higher anxiety about differences in artistic skills within the group activities.

To more clearly understand the two interventions’ contributions on blood pressure (BP) and heart rate (HR), age was removed from the variance in separate mixed-linear models for diastolic blood pressure, systolic blood pressure, and heart rate. The models incorporated the pre- to post-session triplicate measurement averages of blood pressure and heart rate. The eight difference or change scores, one for each intervention session, were used in these models to compare the art and gardening intervention group changes in blood pressure and heart rate over time. The models treated time as a continuous predictor of each cardiac parameter.

There were no statistically significant differences between the group interventions for change in HR after modeling the eight activity sessions (*p* = .435) or the ten total sessions (*p* = .240). Similarly, the art and gardening groups exhibited no statistically significant differences in systolic BP when comparing the intra-session change in systolic BP for the eight activity sessions (*p* = .096) or when comparing the ten total sessions (*p* = .716). Interestingly, when comparing the change for the eight activity sessions, the art group experienced an increase in diastolic BP estimated to be 3 mm Hg greater than the gardening group’s change (*p* = .013). These diastolic BP findings were consistent when comparing the change for the ten full sessions with the art group experiencing an increase in diastolic BP that was 2 mm Hg greater than the gardening group’s change (*p* = .020). Additional models were run to assess if within-group diastolic BP changes were statistically different from that of no change.

Additional statistical models were constructed to analyze each group’s change throughout the eight intervention sessions and the ten total sessions to identify whether intra-session changes were significantly different from 0 (zero), i.e., no change. The multivariate models for the gardening group, both for the eight intervention sessions and ten total sessions, provided evidence to support that no changes occurred concerning HR and systolic and diastolic BP throughout the intervention. However, this was not the case for the art group. For HR, although the art group did not have a statistically significant change within the course of the eight intervention sessions (*p* = .066), there was a 2 mm Hg decrease in BP when assessing the change throughout the ten total sessions (*p* = .006) that includes pre-and post-intervention measurements. In contrast, the art group had significant increases in diastolic BP. Changes were significant when modeling change within the eight intervention sessions (2 mm Hg increase, *p* = .001) and when the pre-and post-session were included (2 mm Hg increase, *p* = .002). While diastolic BP changed significantly throughout the art intervention, systolic BP measurements did not vary considerably throughout either the eight intervention sessions (*p* = .178) or the ten total sessions (*p* = .962).

### Adverse events or unintended harms

There were no observed or reported treatment-mediated harms, adverse events, or unintended effects during the course of this study.

## Discussion

The experimental design of this pilot study was based on an all-female, wellness population, in part, to reduce biological variation and determine whether therapeutic benefit from gardening and or art-making activities could be demonstrated in a healthy population. A study population of only healthy women was selected because there are known cultural, societal, and sex differences in the way males and females respond to interactions with plants. Women appear to have superior object location memory compared to men that is consistent with evolutionarily enhanced foraging for plant foods [52, 53], plant-related consumer behaviors [54, 55], and eating patterns [56]. Research on modern-day hunter-gatherer societies suggests evolutionary-based sex differences in labor where women daily forage and gather fruits, tubers, and other plant-based available food sources. At the same time, men hunt for game intermittently as needed [57]. In a study of images of natural environments versus urban settings, Ulrich [58] observed significant sex differences in the responses of women versus men concerning positive affect and attentiveness. Both responses declined more when women viewed urban scenes compared to men. More women than men agreed with statements that gardening is peaceful/tranquil, and plants make them feel calmer/more relaxed in a study of attitudes towards plants and gardening [59].

Additionally, several fMRI studies appear to provide evidence for sex differences in stimulus processing in the brain, particularly for visual stimuli responses [60–64]. Moreover, some of the best-documented benefits of working and interacting with plants align well with disorders known to be more prevalent in women, such as rumination, depression, anxiety, multiple sclerosis, chronic fatigue syndrome, general fatigue, sleep disturbances, certain eating disorders, and celiac disease [65]. Therefore, we reasoned that if important changes can be observed in a small population of healthy women, then gardening may have equal or even greater therapeutic utility for a range of other clinical populations.

Ideally, in a randomized controlled trial, the attributes of the control group intervention should closely match that of the treatment group. Moore and colleagues [66] have detailed the specific recommendations involving the planning, design and conduct, analysis, and reporting of experimental complex intervention studies. An indoors group-based gardening activity is a complex intervention involving multiple interacting components, including: 1, Concerted active participation to receive the treatment; 2, Belonging to a group of up to ten participants with a shared experience, which involves meeting and getting to know a group of peers during the gardening sessions; 3, Cognitive engagement to learn new information about plants and gardening; 4, Creative expression; 5, Light physical activity; 6, Ambient physical environment; and 7, Engaging in interactions with plants as living, growing entities. A no-treatment control was deemed insufficient to serve as a representative control group for a gardening treatment. An indoor group-based arts activity was chosen as an equally complex intervention possessing all of the gardening intervention attributes except for interactions with plants. Art in a therapeutic context is perhaps more widely recognized and sanctioned as a therapeutic intervention than gardening or HT when practiced within a healthcare environment, and thus may be seen for this study as a form of "treatment-as-usual" to serve as a direct comparator to the gardening treatment. The fundamental difference between the two interventions in this study centers on the fact that gardening involves activities with organisms (plants) that are alive, grow, and produce complex structures with characteristic visual, tactile, olfactory, and in some cases, gustatory traits that are genetically encoded and desirable to humans. As living organisms, plants require attention, care, nurturing, and protection to flourish, thereby fostering emotional connections and attachments on the part of the person. The art sessions in this study were designed to be devoid of living plants or images of plants.

There have been many studies of AT, art-making, HT, and gardening that demonstrate therapeutic benefits to various clinical populations. The present study was conceived and designed to ascertain whether engaging in art or gardening activities would provide quantitatively measurable therapeutic benefits to a population of healthy women. However, as a person becomes physically, mentally, and socially healthier, it becomes more challenging to achieve and measure consequential improvements in health status. Yet the present study provides compelling empirical support for both art and gardening as promoters of potentially meaningful mental health improvements for healthy populations of women between the ages of 26 and 49 years. Of those who identify as gardeners, many anecdotal accounts proclaim the therapeutic nature of gardening. While gardening is a favorite leisure-time activity, engaging in art is similarly popular with roughly 54% of the adults in the U.S. participating in some form of art-making, performance, or art-sharing activities, according to the 2017 Survey of Public Participation in the Arts conducted by the National Endowment for the Arts [67].

While there were positive therapeutic outcomes for both art and gardening, the study results do not support hypothesis 1 as the art and gardening participants responded approximately equally to the different treatments. Nevertheless, there was an almost universal improvement in mean scores (15 out of 16 pre-post comparisons) in the art-making and gardening group scores across the six self-report assessments. However, there were modest differences in some of the outcomes suggesting that with greater sample numbers gardening may outperform art-making as a therapeutic modality for a healthy population of women. Regarding hypothesis 2, scores on the SPDSA assessment improved for both treatment interventions, but did not achieve statistical significance between pre-and post-intervention scores. Therefore, while the study results do not support hypothesis 2, an expanded study population may achieve a more robust statistical outcome.

Some studies conducting plant-based or art-based therapeutic programs have used the same psychometric assessment instruments and found improvements that were comparable to this study. Wichrowski and colleagues [68] reported a near 92% reduction in POMS TMD score after just one gardening session with a post-operative cardiac-inpatient population. Based on a meta-analysis of ten studies related to exercise in nature, Barton and Pretty [69] reported moderate treatment effects size (*d* = 0.54) for improvements in POMS TMD, which is similar in magnitude to the present study (*d* = -0.81 for gardening and *d* = -0.60 for art, where M1 is pre-and M2 are post-intervention means). In studies related to interactions with plants, researchers have also reported improvements in the various subscales of the POMS [68, 70, 71], which support the present findings of improvements on the anger, confusion, fatigue, and tension subscales for the POMS.

Likewise, several studies have reported consistent improvements in mood-related factors based on POMS scores after an art program for breast cancer survivors [20], a general population [25], and for outpatients with a substance abuse disorder [27]. These earlier art studies support the findings in the present study. Our group means for PSS scores decreased by 37% in both the gardening and art intervention groups. These PSS results are comparable to reports from two gardening intervention studies that found improvements in PSS scores to a lesser degree with an 18% reduction in PSS scores for a stressed population [72] and an 8% reduction in PSS scores for a depressed population [73].

Similarly, BDI-II scores in the current study decreased by 66% after the gardening intervention and 43% after the art intervention. Additional confirmatory results from Gonzalez and colleagues [73] indicate that after just four weeks of gardening, BDI-II scores were significantly reduced by nearly 17% for a pooled sample of 46 clinically depressed individuals. Sahlin and colleagues [74] reported that a 16-week intervention of gardening, nature walks, and painting decreased BDI-II scores by nearly 33%.

Conversely, studies involving art interventions have shown mixed results for BDI-II score changes. One study found scores did not change following a few days of art sessions for individuals with PTSD [21], while a second study found nearly an eight-point reduction in BDI-II score (*d* = 0.15) following an eight-week art intervention with inmates [75]. After the gardening intervention, STAI-State Anxiety scores declined by 15%, but no change was observed following the art intervention. Similarly, STAI-Trait Anxiety scores decreased by 10% following the gardening intervention and 1% following the art intervention. Comparable reductions in state anxiety were found in two studies instituting a gardening program with a 5% reduction in STAI-State scores (*d* = 0.21) after 30 minutes of gardening [76] and a 12% reduction in STAI-State scores after twelve weeks of gardening [77]. Neither study reported statistically significant changes pre- to post-intervention. Additionally, Lee and colleagues [77] found a 10% reduction in STAI-Trait scores after a gardening program; however, these results were not statistically significant.

Several studies in the art literature used the STAI, and some reported statistically significant improvements following art interventions [25, 27], while others reported no statistically significant changes after an art intervention [21, 78, 79]. In the present study, SF-36 Physical Health and Mental Health component scores improved by 1% and 4%, respectively, following the art intervention and 2% and 7%, respectively, following gardening. No studies were found that implemented a gardening or art intervention and measured health outcomes with the SF-36; however, results from Monti and colleagues [19] indicated no changes for the SF-36 Physical Health Component, but statistically significant improvements in SF-36 Mental Health Component after an 8-week art and yoga intervention.

This study provides evidence for a dosage response progression towards improved scores throughout the experiment. While the consecutive assessment score reductions are not statistically different from pre-intervention, they are already down after just two one-hour sessions for both art and gardening. One possible explanation for the dosage effect trends for the POMS, PSS, and BDI-II is that group dynamics, i.e., group cohesion and social support, grow more influential over time. As study subjects become more comfortable with each other, they begin to accrue more significant improvements related to the psychological assessments. The growing familiarity and comfort with co-participants were reflected in the anecdotal comments and accounts recorded following the sessions. There is research that shows that increased group cohesion can influence psychological factors. Group cohesion may be affected not only by the activities experienced, but also by the session leader(s), as two artists led the art intervention and a third individual, a horticulturist, led the gardening intervention. The extent to which group cohesion influenced the results of this study was not measured. Therefore, a definitive statement on group cohesion cannot be made.

Interestingly, this explanation was not supported by the lack of changes in the Friendliness subscale of the POMS assessment (**Tables 8** and **9**) and the lack of statistically meaningful change in Satisfaction with Participation in Discretionary Social Activities scores (**Table 5**). Therefore, perhaps another factor mediated the gardening group’s improvements in the last two weeks of the intervention. Another explanation for the changes could be the level of comfort or self-efficacy with the horticultural activities, materials, and methods enjoyed by the gardening cohort. Because there were four themes in the gardening program, namely seeding, vegetative propagation, transplanting, and simulated harvest, perhaps the level of mastery with the activity themes and associated autonomy contributed to the latter improvements. It is also possible that specific activities in the last four gardening sessions were preferred over the four activities at the beginning of the gardening intervention. There is also the possibility that seeing the plants grow and develop over time and having the opportunity to harvest crops allowed for the development of an emotional attachment to the plants, thus resulting in additional psychological benefits.

This study’s third and final hypothesis-driven objective was to determine whether systolic and diastolic blood pressure and heart rate improved during each gardening and art treatment session and following the overall completed interventions. Based on the linear mixed models’ results, the gardening group had more significant reductions in diastolic blood pressure than the art group. In contrast, group differences were not found for either systolic BP or heart rate. More specifically, although positive trends in BP and HR were observed in the gardening group, no improvements were found to be statistically significant at *p* < .05 in the gardening group. The blood pressure findings contrast with other people-plant studies, which found that nature interactions or even viewing nature improved BP and HR [71, 80, 81]. While statistically significant increases in diastolic BP and decreases in HR were found in the present study for the art group, these findings are not supported by another study that found no HR or BP changes following an art intervention [79]. However, the BP results are supported by parallel decreases in perceived stress since blood pressure is a physiological indicator of stress and a predictor of psychological stress [82].

Managing healthcare costs is of paramount importance as Americans spent USD 3.65 trillion on health care in 2018, equaling USD 11,121 per person. Health-related expenditures are projected to rise to about USD 6 trillion by 2027. A unique feature of the SF-36 instrument is its model for calculating predicted medical spending for the four weeks following administering the assessment. This predictive model is based on the scales embedded within the SF-36 and demographic factors such as sex and age; however, the exact formula is not reported in the SF-36v2 3rd Edition Manual. Several studies have used the SF-36 to predict medical expenditure and disease costs burden [83].

In the present study, the predicted four-week medical spending for the gardening intervention decreased with statistical separations (*p* = .034) from pre-intervention (Mean = $143.81; SE = 10.68) to post-intervention predicted expenditures (Mean = $131.18; SE = 7.99). While the art intervention did not yield statistically significant changes in predicted medical spending (*p* = .266), it resulted in an estimated decrease from pre-intervention (M = $128.25; SE = 10.34) to post-intervention medical expenditures (M = $122.02; SE = 7.74). Together these estimates suggest the possibility of potentially significant health-related economic benefits to healthy women and society engaging in regular group-based gardening or art leisure activities in an era of perpetually rising healthcare costs.

## Limitations

The study population of only healthy women was not a random representative sampling of all women in Gainesville or Florida. Women who volunteered to participate in this study may have been a select group who had more free time, more interest in gardening or art, or a greater willingness to participate in the research than women in general living locally. Additionally, the sample did not include men, which also limits the generalizability of the reported results. The sample population was relatively small, which limited the potential to detect some group differences between the two interventions and between within-group pre-intervention and post-intervention outcomes. The small study population of only healthy women may have further limited treatment effect size magnitudes for several of the measured parameters. Therefore, the results cannot be directly generalized to a broader population of women or a more general population.

Participants were not blinded to which treatment group they were randomly assigned.

Although many variables were controlled throughout this experiment, the session leaders for the two interventions differed by necessity between the gardening and art sessions, which may have potentially influenced differences in therapeutic outcomes. Also unknown was what the study participants experienced outside the context of the twice-weekly intervention sessions. Therefore, uncontrolled factors may have influenced physical, psychological, or social elements of the health-related outcomes. Furthermore, this study did not measure the sessions’ relative cognitive or physical components; therefore, unknown differences related to learning or physical activity during the sessions may have influenced the results. The present study had no inactive control group to compare with the two active intervention groups; therefore, only relative effects can be estimated rather than absolute effects.

Since both treatments appear to have provided similar therapeutic benefits, the possibility of a placebo effect cannot be unequivocally ruled in or out.

## Future work

This study’s significant quantitative findings unequivocally justify the continued study of both art and gardening interventions as genuine therapeutic modalities to promote better health and well-being of women that are already relatively healthy. Other studies seeking to conduct experiments with an art or gardening or HT intervention may consider using multiple levels of control groups, incorporating experimental conditions of a concurrent, inactive control group that go about regular daily routines or wait-listed or active control groups of allied therapeutic modalities. Another consideration involves the relative therapeutic effects of passively being in an environment with plants and nature, such as walking in a garden. Future studies may test engagement levels by comparing, observing, and being in a natural area with gardening and horticultural tasks that involve different levels of attention and effort, accounting for the relative quantity (dosage) and quality (diversity of experience) of each intervention. This study implemented group sessions to include any group effects that may contribute to therapeutic outcomes. However, one-on-one sessions could be incorporated into an experimental design to compare differences between group-based art or gardening and engaging in art or gardening individually.

## Conclusions

Engaging in art and gardening activities resulted in apparent therapeutic benefits for healthy women between the age of 26–49 for indicators of depression symptomatology, perceived stress, and total mood disturbance in dosage-mediated responses. The art and gardening interventions were found to improve participants’ psychological profile elements after only four weeks of twice-weekly, one-hour sessions. However, no substantial group differences were revealed between the two interventions. Blood pressure and heart rate were found to be variable, suggesting that a larger sample size appears necessary to detect any apparent treatment differences from pre- to post-intervention for a healthy population of women. When taken together the present findings show that engaging in group-based art or gardening as a leisure activity can further fortify the excellent health status and improve the overall quality and satisfaction of life of healthy women. This study, when added to the growing body of empirical evidence for the therapeutic benefits of engaging in gardening and art-making, provides a compelling basis for the need and support of well-designed large-scale randomized controlled trials that can reveal the actual clinical significance of these two treatment modalities.

## Supporting information

S1 Checklist CONSORT checklist. (PDF)

S1 Fig Ambient, greenhouse and greenhouse heat index temperatures during gardening sessions. (TIF)

S1 Table Physical activity codes and METs for the art and gardening treatments. (DOCX)

S2 Table Normative values for self-report psychometric assessments. (DOCX)

S3 Table De-identified demographic information, psychometric self-report scores, and heart rate and blood pressure measurements datasets. (XLSX)

S1 Document Art sessions handout and instructions. (DOCX)

S2 Document Gardening sessions handout and instructions. (DOCX)

S1 File IRB approved trial. Gardening and Art Protocol Revision 3.
(DOCX)
