# Supplementary figures and images for: A pilot randomized controlled trial of group-based indoor gardening and art activities demonstrates therapeutic benefits to healthy women
Source: PLoS One. 2022 Jul 6;17(7):e0269248. doi: 10.1371/journal.pone.0269248 (PMC9258874; doi:10.1371/journal.pone.0269248)

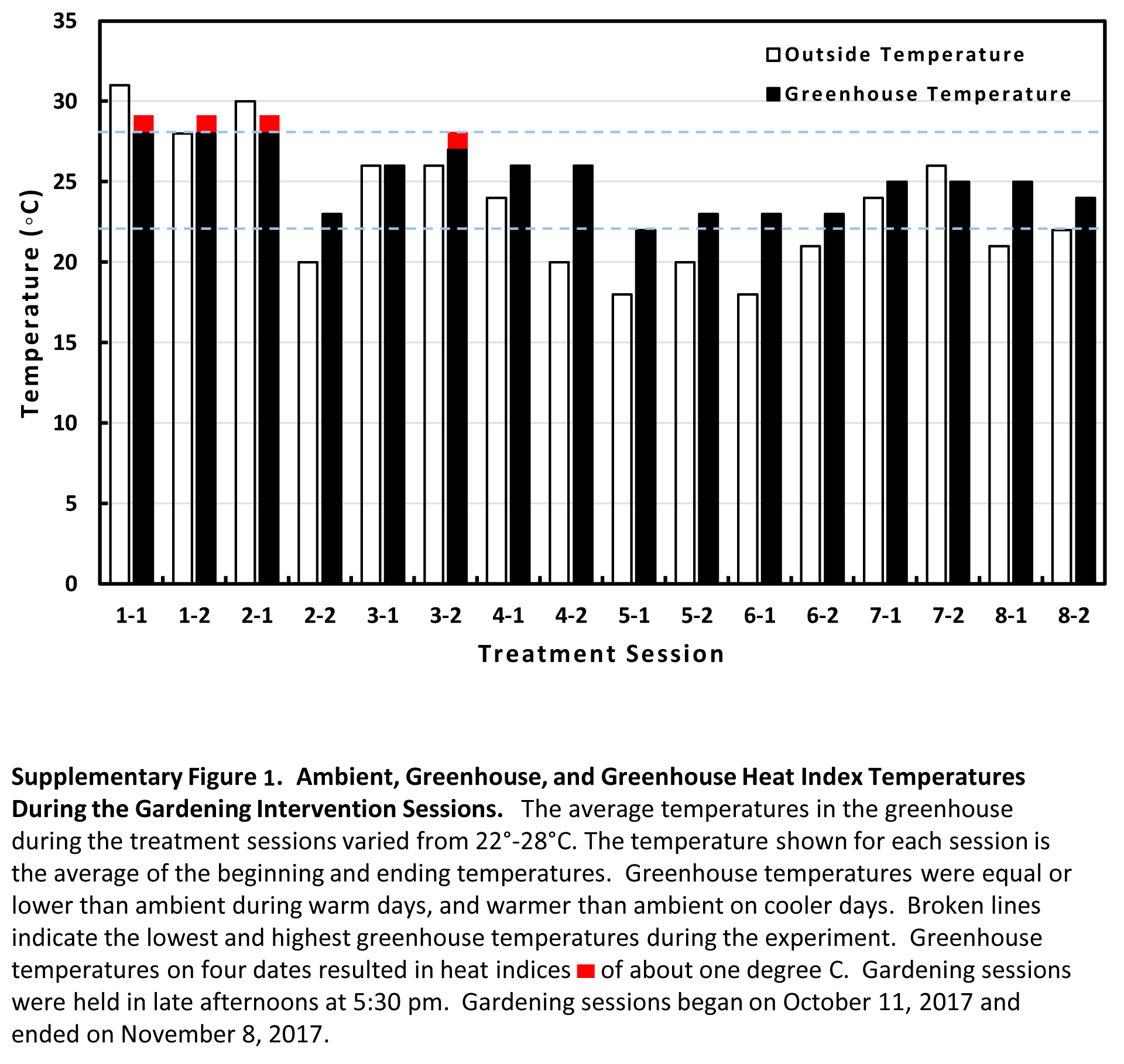

Supplement: S1 Fig — (TIF) [file pone.0269248.s002.tif]
