# Supplementary material for: A pilot randomized controlled trial of group-based indoor gardening and art activities demonstrates therapeutic benefits to healthy women
Source: PLoS One. 2022 Jul 6;17(7):e0269248. doi: 10.1371/journal.pone.0269248 (PMC9258874; doi:10.1371/journal.pone.0269248)
Supplement: S1 Table — (DOCX) [file pone.0269248.s003.docx]

**Supplementary Table S1**

**Physical Activity Codes and METs**

| **Physical Activity MET Codes Representative of the Art and Gardening Activities** | | | |
| --- | --- | --- | --- |
| **Shared Art and Gardening Codes and METs** | | | |
| **Code** | **MET** | **Major Heading** | **Specific Activities** |
| 09030 | 1.3 | Miscellaneous | sitting, reading, book, newspaper, etc. |
| 09060 | 1.3 | Miscellaneous | Sitting, studying, general, including reading and/or writing, light effort |
| 21000 | 1.5 | Volunteer activities | Sitting, meeting, general, and/or with talking involved |
| 05041 | 1.8 | Home activities | Wash dishes, standing or in general (not broken into stand/walk components) |
| 09065 | 1.8 | Miscellaneous | Sitting, in class, general, including notetaking or class discussion |
| 21015 | 2.3 | Volunteer activities | Standing, light work (filing, talking, assembling) |
| 05042 | 2.5 | Home activities | Wash dishes, clearing dishes from table, walking, light effort |
| **Total Shared METs 12.5; Mean MET 1.8** | | | |
|  | | | |
| **Art Only Codes and METs** | | | |
| **Code** | **MET** | **Major Heading** | **Specific Activities** |
| 05080 | 1.3 | Home activities | Knitting, sewing, light effort, wrapping presents, sitting |
| 09020 | 1.8 | Miscellaneous | Drawing, writing, painting, standing |
| 09075 | 1.8 | Miscellaneous | Sitting, arts, and crafts, carving wood, weaving, spinning wool, light effort |
| 09085 | 2.5 | Miscellaneous | Standing, arts and crafts, sand painting, carving, weaving, light effort |
| **Art Only METs 7.4; Mean MET 1.8; Overall Art Mean MET 1.8** | | | |
|  | | | |
| **Gardening Only Codes and METs** | | | |
| **Code** | **MET** | **Major Heading** | **Specific Activities** |
| 08230 | 1.5 | Lawn and garden | Watering lawn or garden, standing or walking |
| 08135 | 2.0 | Lawn and garden | Planting, potting, transplanting seedlings or plants, light effort |
| 05148 | 2.5 | Home activities | Watering plants |
| 08250 | 3.3 | Lawn and garden | implied walking/standing - picking up yard, light, picking flowers or vegetables |
| **Gardening Only MET 9.3; Mean MET 2.3 Overall Gardening Mean MET 2.0** | | | |

The treatment activities of the art and gardening interventions were aligned with the specific activities listed in the Compendium of Physical Activities (Ainsworth et al 2000, 2011) that classifies specific physical activities by the rate of energy expenditure or metabolic equivalent (MET) intensity level. There are seven activity code/MET categories shared by both intervention treatments: 09030/1.3, 09060/1.3, 21000/1.5, 05041/1.8, 09065/1.8, 21015/2.3, and 05042/2.5. The art treatment also included four specific activity codes/MET: 05080/1.3, 09020/1.8, 09075/1.8, and 09085/2.5, as did the gardening treatment 08230/1.5, 08135/2.0, 05148/2.5 and 08250/3.3. Based on informal observations, there may have been slightly more standing and walking in the gardening than the art treatment. While it is not possible to precisely quantify actual time spent for each of the various coded activities, the treatments appear to fall towards the lower half of the light-intensity range (1.6-2.9 METs) (Ainsworth et al 2011).

Ainsworth BE, Haskell WL, Whitt MC, Irwin ML, Swartz AM, Strath SJ, O Brien WL, Bassett DR, Schmitz KH, Emplaincourt PO, Jacobs DR. Compendium of physical activities: An update of activity codes and MET intensities. Med Sci Sports Exercise. 2000; 32(9; SUPP/1): S498-504. <https://doi.org/10.1097/00005768-200009001-00009>. PMID: [10993420](https://pubmed.ncbi.nlm.nih.gov/10993420/)

Ainsworth BE, Haskell W, Herrmann S, Meckes N, Bassett DR, JR, Tudor-Locke C, Greer J, Vezina J, Whitt-Glover, MC, Leon AS. 2011 Compendium of Physical Activities: A second update of codes and MET values. 2011; 43(8): 1575-1581. <https://doi.org/10.1249/mss.0b013e31821ece12>. PMID: [21681120](https://pubmed.ncbi.nlm.nih.gov/21681120/)
