# Supplementary material for: A pilot randomized controlled trial of group-based indoor gardening and art activities demonstrates therapeutic benefits to healthy women
Source: PLoS One. 2022 Jul 6;17(7):e0269248. doi: 10.1371/journal.pone.0269248 (PMC9258874; doi:10.1371/journal.pone.0269248)
Supplement: S2 Table — (DOCX) [file pone.0269248.s004.docx]

**Supplementary Table S2.**

**Supplementary Table 2. Self-report psychometric instrument adult normative values.**

| Instrument | Normative Range | Source |
| --- | --- | --- |
| POMS 2 TMD | 40-59 | Heuchert and McNair, 2012 |
| PSS | 13.7*  16.1* | Cohen and Williamson, 1988  Cohen and Janicki-Deverts, 2012 |
| BDI-II | 9.8  14.6* | Whisman and Richardson, 2015  Beck et al., 1996 |
| STAI-State | 32.9  33.2  35.2 | Nyenhuis et al., 1999  Crawford et al., 2011  Spielberger, 1983 (STAI manual) |
| STAI-Trait | 35.6  36.4  34.8 | Nyenhuis et al., 1999  Crawford et al., 2011  Spielberger et al., 1983 (STAI manual) |
| SF-36 Physical Health | 47-53 | Maruish, 2011. (SF-36v2 Manual) |
| SF-36 Mental Health | 47-53 | Maruish, 2011. (SF-36v2 Manual) |
| SPDSA | 50** | Cella et al 2010 |

*Indicates only women were used to calculate the value. **T-score with an SD of 10.

Heuchert JP, McNair DM. POMS2, Profile of Mood States 2nd Edition™. Multi-Health Systems, North Tonawanda, NY. 2012.

Cohen S, Williamson G. Perceived Stress in a Probability Sample of the United States, S. Spacapan & S. Oskamp Eds. Thousand Oaks, CA: Sage Publications, Inc. 1988.

Cohen S, Janicki‐Deverts D. Who's stressed? Distributions of psychological stress in the United States in probability samples from 1983, 2006, and 2009. J Appl Soc Psychol. 2012; ;42(6):1320-34. <https://doi.org/10.1111/j.1559-1816.2012.00900.x>.

Whisman MA, Richardson ED. Normative data on the Beck Depression Inventory–second edition (BDI‐II) in college students. J Clin Psychol. 2015; 71(9): 898-907. <https://doi.org/10.1002/jclp.22188>. PMID: [25950150](https://pubmed.ncbi.nlm.nih.gov/25950150/)

Beck AT, Steer RA, Ball R, Ranieri WF. Comparison of Beck Depression Inventories-IA and-II in psychiatric outpatients. J Pers Assess. 1996; 67(3): 588-97. <https://doi.org/10.1207/s15327752jpa6703_13>. PMID: [8991972](https://pubmed.ncbi.nlm.nih.gov/8991972/)

Nyenhuis DL, Yamamoto C, Luchetta T, Terrien A, Parmentier A. Adult and geriatric normative data and validation of the profile of mood states. J Clin Psychol. 1999; 55(1): 79-86. <https://doi.org/10.1002/(SICI)1097-4679(199901)55:1%3C79::AID-JCLP8%3E3.0.CO;2-7>. PMID: [10100834](https://pubmed.ncbi.nlm.nih.gov/10100834/)

Crawford J, Cayley C, Lovibond PF, Wilson PH, Hartley C. Percentile norms and accompanying interval estimates from an Australian general adult population sample for self‐report mood scales (BAI, BDI, CRSD, CES‐D, DASS, DASS‐21, STAI‐X, STAI‐Y, SRDS, and SRAS). Aust Psychol. 2011; 46(1): 3-14. <https://doi.org/10.1111/j.1742-9544.2010.00003.x>.

Spielberger CD, Gorsuch R, Lushene R, Vagg P, Jacobs G. Manual for the State-Trait Anxiety Inventory. Consulting Psychologists Press, Palo Alto, CA. 1983.

Maruish ME. User's Manual for the SF-36v2 Health Survey. 3rd ed: Quality Metric Incorporated. 2011.

Cella D, Riley W, Stone A, Rothrock N, Reeve B, Yount S, et al. The patient-reported outcomes measurement information system (PROMIS) developed and tested its first wave of adult self-reported health outcome item banks: 2005–2008. J Clin Epidemiol. 2010; 63(11): 1179-94. <https://doi.org/10.1016/j.jclinepi.2010.04.011>. PMID: [20685078](https://pubmed.ncbi.nlm.nih.gov/20685078/)
