# Supplementary material for: A pilot randomized controlled trial of group-based indoor gardening and art activities demonstrates therapeutic benefits to healthy women
Source: PLoS One. 2022 Jul 6;17(7):e0269248. doi: 10.1371/journal.pone.0269248 (PMC9258874; doi:10.1371/journal.pone.0269248)
Supplement: S1 Document — (DOCX) [file pone.0269248.s006.docx]

**Supplementary Document 1.**

Gardening and Art Study

Art Session Handouts

Wilmot Gardens Conference Center

University of Florida

Study Staff: Kris Sullivan, BS; Dylan Klempner, BS & MFA; Jill Sonke, MA

Study Coordinator: Raymond Odeh, BS

Principal Investigator: Charles Guy, PhD

# Table of Contents

[Table of Contents 2](#_Toc488417337)

Welcome to the Study 3

Contact Information 3

[Art Sessions Information 4](#_Toc488417338)

[Timeline Sequence for Eight Art Sessions 4](#_Toc488417339)

[Art Activity Handouts and Instructions 5](#_Toc488417340)

[Art Session 1: PAPERMAKING: Creating Handmade Paper from Recycled Materials 5](#_Toc488417341)

[Art Session 2: IMAGE TRANSFER: Transferring images into artwork 7](#_Toc488417342)

[Art Session 3: VISUAL STORYTELLING: Sequential Arts 9](#_Toc488417343)

[Art Session 4: LINOCUT PRINTMAKING, PART 1: Carving the Matrix 12](#_Toc488417344)

[Art Session 5: LINOCUT PRINTMAKING, PART 2: Printing the Matrix 14](#_Toc488417345)

[Art Session 6: PAPER BATIK: Creating patterns with resist 16](#_Toc488417346)

[Art Session 7: MIXED MEDIA COLLAGE: Writing/Visual Art Activity 18](#_Toc488417347)

[Art Session 8: SENSATION DRAWING: Sensorial Perceptions 20](#_Toc488417348)

[Glossary of Key Terms 22](#_Toc488417349)

**Cover illustration by Raymond Odeh**

# Welcome to the Study

First, allow our research team to welcome you to the Gardening and Arts Study. We value your time and participation in this research, and if at any time you have questions or concerns regarding your participation in the study, feel free to contact Study Coordinator Raymond Odeh or Principal Investigator Charles Guy. Our contact information is below.

Now, let us provide you with some guidelines regarding your participation in this study:

- Please refrain from engaging in gardening or art activities including visiting botanical gardens and art museums outside of the study sessions
- Please refrain from using the Internet to find and access information about gardening or arts activities during the time following the orientation and until after the final wrap-up session
- Please do not walk through the grounds at Wilmot Gardens outside of study sessions
- You may use the (*The Artist’s Handbook*) during the study as a resource and reference to find an answer to a question you may have, but avoid doing extensive reading during the time from the orientation and until after the wrap-up session
- Please do not take any photographs or make any recordings that may reveal the identity of any study subject as this will result in a protocol deviation that must be reported to the IRB
- Please inform the Study Coordinator, Raymond Odeh or the Principal Investigator, Charles Guy, if you experience any major life event that changes your daily life during the course of the study
- If you miss more than 2 of the 8 arts sessions, you may be withdrawn from the study, it is not possible to offer a make-up session under the approved procedures of the study

This manual is intended to help you navigate each session and provides you with additional resources if you choose to continue engaging in gardening or arts activities after your active participation in this study is completed. Complementing this manual, we have provided you with a reference book, *The Artist’s Handbook* to serve as a supplement to the information provided in this manual.

# Contact Information

Study Coordinator| Raymond Odeh (352)273-4525 [rodeh@ufl.edu](mailto:rodeh@ufl.edu)

*Mr. Odeh, graduate student, working on a Master of Science in Horticulture with a degree from the University of Florida specialized in Landscape and Nursery Horticulture can answer questions regarding session details and research protocol.*

Principal Investigator| Charles Guy (352) 273-4528 [clguy@ufl.edu](mailto:clguy@ufl.edu)

*Dr. Guy, Assistant Chair and Professor of Plant Physiology and Biochemistry in the Department of Environmental Horticulture with research interests in people-plant interactions and the biochemical and molecular responses of plants to unfavorable temperature conditions can answer any questions about this study.*

# Art Sessions Information

Mondays/Wednesdays or Tuesdays/Thursdays at 5:30 PM to 6:30 PM

Conference Center at Wilmot Gardens

Corner of Gale Lemerand Drive and Mowry Road, just North of Archer Road

Please park in available parking in one of the three locations circled below:

**
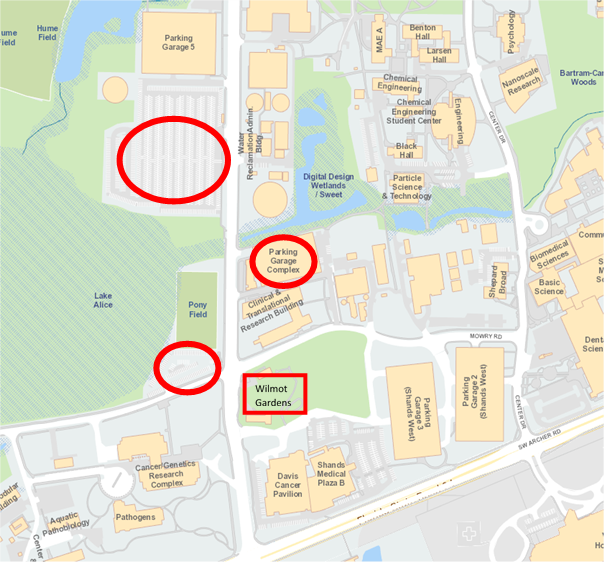
**

Mowry Road

Gale Lemerand Drive

**N**

Modified from online UF campus map <https://campusmap.ufl.edu/#/>. “© OpenStreetMap contributors”, <https://www.openstreetmap.org/copyright> (CC BY-SA 2.0)

# Timeline Sequence for Eight Art Sessions

| Timeline | Session Component |
| --- | --- |
| 0 min | Arrival and Sign-in/Pick up Name Tag |
| 0-10 min | Individuals Review Previous Activities (i.e. plant growth)/Instructors Greet Individuals and Assemble Group/Heart Rate and Blood Pressure Measurements Recorded |
| 10-20 min | Educational Module/Introduction to Activity/Demonstration of Activity as Warranted (booklet, instruction sheet, resource material handouts) |
| 20-50 min | Art Activity, Questions |
| 50-70 min | Clean-up/Heart Rate and Blood Pressure Measurement Recorded/  POMS and PSS Assessments (Even-numbered Sessions)/  BDI (Sessions 4 and 8)/  Departure |

# Art Activity Handouts and Instructions

## **Art Session 1:** Activity, Goals, Materials, Supplies and Instructions for Exercise

| **Session 1** | **PAPERMAKING:** Creating Handmade Paper from Recycled Materials |
| --- | --- |

**Hand Papermaking**

Paper fibers are cellulose and are made from plant materials. To make new paper from old paper, you will simply reverse the papermaking process. Dry paper is put into water to weaken the hydrogen bonds, electrostatic interactions and induced dipoles between cellulose fibrils. When agitated in the blender, the fibers let go of one another and become single fibers in water called pulp. When the pulp is scooped into hand mold and water is removed allowing it to dry, the bonds and interactions reform and finishes a complete cycle.

This exercise will introduce basic papermaking techniques and materials.

**Educational Purpose:** This exercise will explore basic handmade papermaking techniques and highlight one of the oldest recycling practices. Paper dates back to 105 AD in China and was made by hand until industrial manufacturing was introduced in 1803. Hand papermaking continues to be a specialized craft today, and is widely used as a medium for artistic expression. Participants will learn how to create a paper pulp from recycled papers and cotton linter using a household blender. They will experiment with color options and provided additives before pulling sheets of handmade paper using a dip hand mold. Papers will be transferred to couch sheets and left to dry until a subsequent workshop. Participants will also be shown a basic pour method utilizing a tin can mold to increase accessibility of papermaking at home without specialized materials.

**Social Interactions:** Participants will interact with session leaders and other participants. In this first session, participants will be asked to share their first name only with the group. Mechanism for interactions will include questions and comments between and among leaders and participants. Participants will be sharing pulp vats and additives with one another.

**Goals/Benefits:** Facilitate participant-art medium interactions, increase knowledge; stimulate cognitive abilities and skills; increase concentration; enhance motor skills (hand-eye coordination); meet new people; experience individual and group interactions; and enjoy activity.

**Materials and Supplies:**

- Water
- Scrap Papers
- Plastic storage tub or vat
- Drain pan
- Kitchen blender (recommended to dedicate an inexpensive blender to papermaking)
- Mold & Deckle
- sponge
- press bar
- Couch sheet or other absorbent material

**Considerations for Making Paper with Recycled Materials:**

- Drawing, printmaking, and watercolor papers are the best scrap papers to use because they are generally made from cloth rag and not chemically treated tree fiber; however, any and all paper can be recycled.
- You will not want to use your kitchen blender to make pulp. It is best to purchase an inexpensive blender and dedicate it to papermaking.

**Instructions for Making Recycled Paper with a Dip Hand Mold:**

1. Cut or rip up your paper into about 1 inch squares. Ideally, you should soak your paper for a few hours or overnight for easy blending.

2. Throw in a good handful or two of the cut up scrap papers. Fill up a kitchen blender with water and blend until it is a pulp. Fill up the tub 1/3 to 1/2 way with your blended pulp. Add more water to the bin. The more pulp in your slurry, the thicker your paper will be.

3. Stir your vat of pulp. Hold the mold screen side up, and place the deckle evenly on top. Holding them together at a 45-degree angle, dip the mold and deckle to the bottom of the vat and scoop up, holding the mold and deckle horizontally. As you lift it out of the slurry, give it a quick shake back and forth, and left to right to align the fibers and make a uniform sheet. Stop shaking before the sheet is fully drained. Let the water drain to a drip.

4. Set the deckle aside and carefully put the cover screen over the new sheet resting it on the screen support. Press a sponge firmly down on the cover screen. Wring the sponge and press again. Continue until you have removed most of the water.

5. Lift a corner of the cover screen carefully and peel it off slowly. Pick up the papermaking screen with the new sheet on it and turn it over onto your couch sheet.

6. Press sponge firmly all over the screen’s surface, removing additional water. Slowly lift one corner and peel off the screen from the damp sheet.

7. Place a second dry couch sheet on top of your sheet. With a sponge, press gently at first, then press firmly with as much pressure as possible. Use a press bar to press your paper even more. Remove second couch sheet.

8. Let paper dry full and remove couch sheet.

**Resources:**

- “Trash to Treasure Papermaking” by Arnold Grummer, Storey Publishing, North Adams, MA. (2011)
- Arnold Grummer’s YouTube Channel: [www.youtube.com/arnoldgrummer](http://www.youtube.com/arnoldgrummer)
- Hand Papermaking, Inc: <http://handpapermaking.org>

## Art Session 2: Activity, Goals, Materials, Supplies and Instructions for Exercise

| Session 2 | IMAGE TRANSFER: Transferring images into artwork |
| --- | --- |

**Image Transfer**

An image transfer is the act of transferring an image from one source to another. Artists frequently create with reference images, and there are numerous ways to incorporate existing imagery directly into one’s work. Utilizing a transfer technique can be an inspiration for art making, as well as having direct applications in beginning a project.

This exercise will introduce a basic image transfer technique and materials for mixed media and printmaking projects. High contrast photocopies will be transferred onto canvas and linoleum with a gel medium. Gel medium is essentially a colorless acrylic paint that can additionally be used as an adhesive or sealant. Photocopies are created with toner and utilize a heat process to set the toner. Instead of absorbing into the fibers of the paper, toner sets on top allowing it to release once coated in medium.

**Educational Purpose:** Participants will learn a basic image transfer process for use in art, mixed media and design. Individuals will be provided with a canvas panel substrate and use gel medium to transfer found images onto the panel. They will also select a found image to transfer onto easy cut linoleum for a printmaking workshop to follow. Found images will be a variety of printed images sourced from the Internet and books. The transfer method includes brushing gel medium onto the panel, placing the image face side down, and softly burnishing to adhere. The pieces will be left to dry for use in future printmaking and mixed media collage workshops. Paper will be removed when dry leaving images on the canvas panel and linoleum substrates.

**Social Interactions:** Participants will interact with session leaders and other participants. Mechanism for interactions will include questions and comments between leaders and participants.

**Goals/Benefits:** Facilitate participant-art medium interactions, increase knowledge; stimulate cognitive abilities and skills; increase concentration, restore directed attention; enhance motor skills (hand-eye coordination); experience group interactions; enjoy activity.

**Materials and Supplies:**

- Photocopies of found images
- Matte gel medium
- Paintbrush
- Canvas panel substrate
- Linoleum substrate
- Brayer
- Sponge
- Water

**Considerations for Image Transfer:**

- High contrast images work best for all image transfers, both color and black and white.
- Laser prints and photocopies are recommended as they utilize toner, which makes cleaner transfer.
- Smooth, porous surfaces receive transfers best.
- Direct gel medium transfers reverse the image, so be sure to mirror copy if it includes text.

**Instructions for Transferring Images:**

1. Select an image from provided photocopies.
2. Apply a generous coat of gel medium to the canvas panel with brush.
3. Place photocopy face down on the gel and smooth gently with brayer to adhere.
4. Allow the gel to dry.
5. Dampen the back of the photocopy with water. Use your fingers or a small sponge in a circular motion to remove the paper leaving the transferred image.

**Resources:**

- “Playing with Image Transfers” by Courtney Cerruti, Quarry Books, Beverly, MA. (2013)
- Courtney Cerruti’s image transfer class: [www.creativebug.com](http://www.creativebug.com)

## Art Session 3: Activity, Goals, Materials, Supplies and Instructions for Exercise

| Session 3 | VISUAL STORYTELLING: Sequential Arts |
| --- | --- |

**Visual Storytelling**

This exercise will explore techniques for making basic comics, also called Sequential Arts. Rome’s Trajan’s Column, which commemorates Emperor Trajan’s victory in the Dacian Wars and was dedicated in 110 AD, is considered one of the earliest example of modern comics. Visual images presented in a sequential narrative structure date back even earlier to Egyptian hieroglyphics and Greek friezes. In the middle ages, as Christianity grew throughout Europe, images relating Bible stories were often used for the illiterate. With the invention of the printing press, comics developed alongside the printed word. Cartoonists began depicting political and social life through storytelling and satire by the 17^th^ and 18^th^ centuries. Comics’ popularity continued to grow throughout the 20^th^ century. They were printed in newspapers and as stand-alone comic books. Today, the art form continues to grow and evolve into a variety of innovative forms including graphic novels and digital comics.

**Educational Purpose:** Participants will learn how to create a simple three-panel comic strip. They will experiment with generating ideas for narratives that can be developed through both words and images.

**Social Interactions:** Participants will interact with session leaders and other participants. Mechanism for interactions will include questions and comments between leaders and participants. Participants will be sharing writing and visual art supplies with one another.

**Goals/Benefits:** Facilitate participant-art medium interactions, increase knowledge; stimulate cognitive abilities and skills; increase concentration; restore directed attention; develop ability to form basic narrative structures; experiment with basic drawing techniques; enhance motor skills (hand-eye coordination); experience group interactions; enjoy activity.

**Materials and Supplies:**

- Pencils
- Paper

**Consideration for Visual Storytelling:**

- In brainstorming, allow yourself to be guided by your own intuition without thinking about whether you are right or wrong.

**Instructions for Visual Storytelling:**

This activity was adapted from the online course *Storytelling Flow* with instructor Tom Hart, a cartoonist, best-selling author, and Director of The Sequential Artists Workshop.

1. According to cartoonist, Tom Hart, the first step to starting a comic is to generate ideas by writing lists. Write between 1-3 items for each category below. One is fine, three may be better. Always write the first thing that comes to your mind. Write whatever comes in 30-60 seconds.

- Start with something distracting you right now
- Write your favorite Urban Phenomenon, like chain-link fences, or banks, or tall buildings or whatever
- Write your favorite rural phenomenon, like morning birds, or big skies full of clouds, or whatever you may wish to choose.
- Invent a society. This is one of the crazier things I am asking, but just try it. In 1 or 2 minutes, invent some part of a society. Maybe it’s society of 12-year-old hunter-gatherers who are under threat from robots, or a city in which people sell and trade their foot lice. Just come up with something. As with everything, write the first thing that comes to mind.
- What you would be if everything were awesome for you? Would you be a fabulous unicorn, a glamorous rock star, or merely someone who flies and is adored by all? Just write it.

If any of the above frustrate you or freak you out, draw instead from this list:

- Your favorite machinery
- A big win
- A big fail
- A time you were lost in a crowd
- A beautiful thing you saw

2. Draw anything in that list of ideas. Doodle characters, doodle actions, draw anything that comes to mind, whether it’s from the list or not.

3. Take your favorite doodle, whichever one speaks to you the most, and draw it again on a new sheet of paper somewhere, making sure that there is something active going on. It cannot just be a character, but needs to be a character doing something. This is important, just make sure that there is something happening, even if it’s staring, or holding something up, or it could be more extreme: fighting, loving, etc. This is our Narrative Image that we will use below.

4. Divide a piece of paper into 3 rectangular panels. Then divide each panel into 3 sections.

5. Draw your single image in the box in the center of each panel.

6. Under the left box of each panel, write one possible thing that might have happened right BEFORE this image.

7. Under the right box of each panel, write one possible thing that might have happened right AFTER this image.

8. Use the blank three-panel comic sheets, make three single comics using the character from #3 above. If you struggle to think of BEFORE and AFTER images, choose ideas from the list below:

- One that focuses on a surprised reaction
- One that focuses on a hiding something
- One that focuses on discovering something
- One that focuses on a decision
- One that focuses on a transfer of power
- One that focuses on a disappointment or loss

**Resources:**

- Sequential Artists Workshop online classes: <http://sequentialartistsworkshop.thinkific.com/collections>
- Sequential Artists Workshop: <http://www.tomhart.net/saw.html>
- Tom Hart’s personal website: <http://www.tomhart.net/>

## Art Session 4: Activity, Goals, Materials, Supplies and Instructions for Exercise

| **Session 4** | **LINOCUT PRINTMAKING, PART 1: Carving the Matrix** |
| --- | --- |

**Linocut Printmaking, Part 1**

Printmaking is the process of transferring an image from a matrix to a substrate, most often with the possibility for replication. There are many forms of printmaking and each has its own origin. It is hard to pinpoint exactly when printmaking started historically, as engravings go all the way back to prehistoric cave art. Before the invention of the printing press, early printmaking techniques were more likely considered a means of communication than an art form. In the 18th century, art prints began to be considered original works of art. Linoleum was first invented in the 1860s. It was first used as a medium for printing in the early 1900s, when Germans used it for making patterns on wallpaper.

Linoleum printing, a form of relief printing, is one of the easiest and most direct of all the printmaking methods. Linocuts can be simplistic and graphic, or as intricately detailed as you want. It is a subtractive process, meaning you cut away, or subtract, the areas you do not want to print. They can be printed onto almost any type of paper or fabric.

**Educational Purpose:** Participants will learn a basic form of relief printmaking. Linoleum printing is a subtractive method, where individuals cut away images transferred on a previous workshop. Individuals will use a specialized cutter with various blades, and explore cutting techniques to achieve detail and shading.

**Social Interactions:** Participants will interact with session leaders and other participants. Mechanism for interactions will include questions and comments between leaders and participants.

**Goals/Benefits:** Facilitate participant-art medium interactions, increase knowledge; stimulate cognitive abilities and skills; increase concentration, restore directed attention; enhance motor skills (hand-eye coordination); experience group interactions; enjoy activity.

**Materials and Supplies:**

- Prepared linoleum
- Speedball cutter with various blades
- Dry paintbrush

**Considerations for Linocut Printmaking:**

- Carving tools are very sharp, so always remember to cut slowly and away from your body.
- If you are unsure about how something will look printed, you can do a practice print and resume carving if you are unsatisfied.
- How one holds a carving tool is a matter of personal preference and will become more natural as one spends time carving. It is best to start holding the tool as you would a pencil to give you the most control.

**Instructions for Linocut Printmaking:**

1. Based on the image you have transferred onto the linoleum, decide whether you wish to print a positive or negative image. For positive images, you will cut away the background, and for negative images, you will cut away the image leaving the background to print.
2. Using a Speedball handle and the accompanying blades, start carving using small strokes away from your body. Rotate the linoleum or matrix to maintain a carving motion away from the body.
3. Clean off any stray pieces of linoleum with a dry paintbrush.

**Resources:**

- “Block Printing” by Sandy Allison and Robert Craig, Stackpole Books, Mechanicsburg, PA. (2011)

## Art Session 5: Activity, Goals, Materials, Supplies and Instructions for Exercise

| Session 5 | LINOCUT PRINTMAKING, PART 2: Printing the Matrix |
| --- | --- |

**Linocut Printmaking, Part 2**

Printmaking is the process of transferring an image from a matrix to a substrate, most often with the possibility for replication. There are many forms of printmaking and each has its own origin. It is hard to pinpoint exactly when printmaking started historically, as engravings go all the way back to prehistoric cave art. Before the invention of the printing press, early printmaking techniques were more likely considered a means of communication than an art form. In the 18th century, art prints began to be considered original works of art. Linoleum was first invented in the 1860s and was first used as a medium for printing in the early 1900s, when Germans used it for making patterns on wallpaper.

Linoleum printing, a form of relief printing, is one of the easiest and most direct of all the printmaking methods. Linocuts can be simplistic and graphic, or as intricately detailed as you want. It is a subtractive process, meaning you cut away, or subtract, the areas you do not want to print. They can be printed onto almost any type of paper or fabric.

**Educational Purpose:** Participants will create prints from their linoleum matrix. The process includes applying paint to a palette, and using a brayer to apply paint to the linocut. Brayers are hand rollers used to evenly distribute paint on the palette. Paper is then placed on top of the linocut and the image is burnish onto the paper by using a baren in a circular motion on the back of the paper. Barens are traditional, handheld printmaking tools used for burnishing. Participants will check their prints and potentially do more carving until desired effects are achieved. Several prints will be made of each image.

**Social Interactions:** Participants will interact with session leaders and other participants. Mechanism for interactions will include questions and comments between leaders and participants.

**Goals/Benefits:** Facilitate participant-art medium interactions, increase knowledge; stimulate cognitive abilities and skills; increase concentration, restore directed attention; enhance motor skills (hand-eye coordination); experience group interactions; enjoy activity.

**Materials and Supplies:**

- Prepared linoleum substrate
- Dry paintbrush
- Speedball cutter with various blades
- Piece of Plexiglas
- Water-based printmaking ink
- Brayer
- Several sheets of paper
- Baren

**Considerations for Linocut Printmaking:**

- If you are unsure about how something will look printed, you can do a practice print and resume carving if you are unsatisfied.
- Carving tools are very sharp, so always remember to cut slowly and away from your body.
- The first print you pull won’t likely be the best, so try a few prints before making any judgements about your work.

**Instructions for Linocut Printmaking:**

1. Brush off carved linoleum matrix removing any stray carvings.
2. Choose a water-based printmaking ink color and apply to Plexiglas. Use your brayer to roll out the ink into an evenly coated rectangle.
3. Use the charged brayer to roll ink onto the linoleum matrix. It will take several passes to get the linoleum fully covered and you may need to charge the brayer again during this initial inking.
4. With clean hands, pull your first print by lining up a piece of paper on top of the linoleum. Lightly burnish the back of the paper in a circular motion with the printmaker’s baren.
5. Carefully peel back the paper from the linoleum. Evaluate your image and decide if it needs more carving. If so, wash and dry the linoleum before carving.
6. Continue to pull prints by re-inking the linoleum for each new print.

**Resources:**

- “Block Printing” by Sandy Allison and Robert Craig, Stackpole Books, Mechanicsburg, PA. (2011)

## Art Session 6: Activity, Goals, Materials, Supplies and Instructions for Exercise

| **Session 6** | **PAPER BATIK: Creating patterns with resist** |
| --- | --- |

**Paper Batik**

Batik is a textile art form in which wax is applied to cloth before paint or dye is applied. Once the paint is dry, the wax is removed, leaving an imprint where the wax resisted the paint. Early examples of batik have been found in the Far East, Middle East, Central Asia, and India from over 2000 years ago. Traditionally where batiks are commonly made, regions have their own patterns.

This exercise will introduce the concept of batik utilizing simple materials. Creating batik-style art on paper is a fantastic way to explore composition with minimal investment.

**Educational Purpose:** Participants will learn principles of batik, a method of producing a colored design using a resist. Instead of using wax on fabric, participants with use a masking fluid to create a ghost design or resist on paper. Watercolor will then be applied to images by direct application with a brush.

**Social Interactions:** Participants will interact with session leaders and other participants. Mechanism for interactions will include questions and comments between leaders and participants.

**Goals/Benefits:** Facilitate participant-art medium interactions, increase knowledge; stimulate cognitive abilities and skills; increase concentration, restore directed attention; enhance motor skills (hand-eye coordination); experience group interactions; enjoy activity.

**Materials and Supplies:**

- Watercolor paper
- Pencil
- Masking fluid
- Color shaper
- Watercolor paint
- Paintbrush
- Soft rubber eraser

**Considerations for Paper Batik:**

- If you choose to draw the pattern in pencil in advance, you will want to draw very lightly as dark pencil lines will show through once the masking fluid is removed.
- Using darker tones of watercolor paint next to the masking fluid design will create a more dramatic pattern.
- Be sure to let the watercolor paint dry completely before removing the masking fluid.

**Instructions for Paper Batik:**

1. Create a repeating pattern on the provided paper lightly with pencil and then with masking fluid. Let the fluid dry for several minutes until it has completely set.
2. Create a watercolor wash and cover paper over dried resist.
3. Once watercolor paint is full dry, lightly remove masking fluid with soft eraser or finger.

**Resources:**

- “The Artist’s Handbook, 3^rd^ Edition” by Ray Smith, DK Publishing, New York, NY. (2003)

## Art Session 7: Activity, Goals, Materials, Supplies and Instructions for Exercise

| **Session 7** | **MIXED MEDIA COLLAGE:** Writing/Visual Art Activity |
| --- | --- |

**Mixed Media Collage**

This exercise will explore techniques for making collages. Though the term “collage” was coined by Pablo Picasso and Georges Braque in 1912, its origins date back to the invention of paper in China around 2000 BC. In 10^th^ Century Japan, poets used glued pieces of text. Gold leaf and gemstones were applied to religious images in medieval Europe. Evidence of hobbyists using collage for memorabilia began in the 19^th^ century. Contemporary collage in art occurred with the rise of modernism in the early 20^th^ century. Through the technique, these artists brought a collision of fragmented meaning by incorporating newspaper and other found materials into their work.

**Educational Purpose:** Participants will learn how to create simple collages, which they will use for expressive and/or reflective writing.

**Social Interactions:** Participants will interact with session leaders and other participants. Mechanism for interactions will include questions and comments between leaders and participants. Participants will be sharing writing and visual art supplies with one another.

**Goals/Benefits:** Facilitate participant-art medium interactions, increase knowledge; stimulate cognitive abilities and skills; increase concentration; restore directed attention; develop ability to form basic narrative structures; experiment with basic drawing techniques; enhance motor skills (hand-eye coordination); experience group interactions; enjoy activity.

**Materials and Supplies:**

- Pencil
- Pens
- Prepared canvas panel
- Scrap papers
- Scissors
- Acrylic gel medium
- Acrylic paints
- Paint brush
- Magazines
- Embellishments (glitter, stamps, found objects, etc.)
- Writing paper

**Consideration for Mixed Media Collage:**

- Layering images can create interesting depth.

**Instructions for Mixed Media Collage:**

1. Begin by revisiting your image transfer on the canvas panel and consider a theme. Start cutting out images from magazines and looking through scrap papers and embellishments. Do not think too much, about which images might work best. Instead, allow the search to be an organic process.

2. When you are satisfied with the number of images and additional materials you have selected, begin assembling and gluing them onto your prepared canvas panel with gel medium. Coat the surface of the panel with gel medium. Position your images and papers into the wet gel on the panel. Coat the item with another layer of gel medium from the center outward, using the brush to press down and smooth any bubbles. Continue with addition images and objects.

3. When you are prompted (after 40 minutes) to do so, set aside your collage materials and take up your pen and writing paper. Use the remaining class time to write. Consider thoughts or ideas that may have arisen because of the collage activity.

**Resources:**

- The International Museum of Collage, Assemblage and Construction:

<http://collagemuseum.com>

- Kolaj Magazine, which focuses on contemporary collage:

<http://kolajmagazine.com/content/>

- Collage Discovery Workshop by Claudine Hellmuth, North Light Books, Cincinnati, Ohio. (2003)

## Art Session 8: Activity, Goals, Materials, Supplies and Instructions for Exercise

| **Session 8** | **SENSATION DRAWING:** Sensorial Perceptions |
| --- | --- |

**Sensation Drawing**

**Educational Purpose:** This exercise will explore expressive drawing techniques. Participants will broaden their experiential knowledge of drawing materials by incorporating sensorial perceptions. They will experiment with line, pattern, and color to represent their felt experience.

**Social Interactions:** Participants will interact with session leaders and other participants. Mechanism for interactions will include questions and comments between leaders and participants. Participants will be sharing drawing materials with one another.

**Goals/Benefits:** Facilitate participant-art medium interactions, increase knowledge; stimulate cognitive abilities and skills; stimulate sensorial expression, increase concentration, restore directed attention; enhance motor skills (hand-eye coordination); experience group interactions; enjoy activity.

**Materials and Supplies:**

- Drawing paper
- Graphite Pencils
- Pens
- Colored Pencils
- Markers

**Considerations for Sensation Drawing:**

- Focus on non-visual sensations
- For this activity you can take two approaches;
  1. Pay close attention to sensations found in your environment
  2. Imagine what it’s like to experience various sensations

**Instructions for Sensation Drawing:**

This activity was adapted from the Stanford University’s online course, *Drawing Inspiration: Developing a Creative Practice* with the artist and instructor, Trevor Tubelle.

1. Get comfortable and recognize your non-visual senses.

2. Divide up a piece of drawing paper into four equal sections and label each section with one of your non-visual senses (hearing, taste, smell, and touch).

3. Focus on or imagine one of your senses at a time, e.g., close your eyes while imagining the taste of some spicy food or sound of calming music, the feel of a soft textured sweater, etc. Or, try to be focused and acutely conscious of your body’s reactions and associations. Focus on the sensation of the hard seat beneath you or the quiet sounds of people working and speaking softly.

4. Respond to the stimuli by drawing marks that represent or express each sensory experience in a literal or metaphorical way and fill the corresponding section on the page.

5. If you have time, do this for all your non-visual senses at least three times, which means that you would divide and fill three pages with marks.

As an example, if I was imagining drinking strong coffee I would go to the area on the page labeled “taste” and draw marks that visually show how that coffee tastes on my tongue as the hot liquid goes down my throat (I imagine lots of dark, energetically clustered and smooth flowing lines). It can be an odd, and sometimes challenging, experience to translate non-visual sensory information into visual marks, but in essence, we are doing this all the time when we are drawing or writing.

**Resources:**

- Trevor Tubelle’s Website: <http://tubelle.com/>
- Link to Stanford University’s online course Drawing Inspiration: Developing a Creative Practice: <http://online.stanford.edu/course/drawing-inspiration-developing-creative-practice>

# Glossary of Key Terms

Baren= Traditional printmaking tool used for burnishing.

Batik= Technique of wax-resist dyeing cloth.

Brayer= Hand tool used in printing, printmaking, and collage.

Collage= Artwork created from an assemblage of different parts or materials.

Deckle= Removable top part of a hand mold, used to determine sheet’s paper and size in papermaking.

Gel Medium= Binder that used in acrylic paints without any pigment added for color. Can be used as transfer medium, paint thickener, adhesive or sealant.

Hand mold= Tool for making paper by hand, consisting of a screen, screen support and deckle

Linocut= Design carved in relief on a block of linoleum.

Masking Fluid= Solution of latex in ammonia used to block out areas of a watercolor while painting.

Substrate= Substance or layer that underlies something.
