# Supplementary material for: A pilot randomized controlled trial of group-based indoor gardening and art activities demonstrates therapeutic benefits to healthy women
Source: PLoS One. 2022 Jul 6;17(7):e0269248. doi: 10.1371/journal.pone.0269248 (PMC9258874; doi:10.1371/journal.pone.0269248)
Supplement: S2 Document — (DOCX) [file pone.0269248.s007.docx]

**Supplementary Document 2.**

Gardening and Art Study

Gardening Session Manual

Wilmot Gardens Greenhouse

University of Florida

Study Coordinator Raymond Odeh, BS

Principal Investigator Charles Guy, PhD

# Table of Contents

[Table of Contents 2](#_Toc489445122)

[Welcome to the Study 3](#_Toc489445123)

[Contact Information 3](#_Toc489445124)

[Gardening Sessions Information 4](#_Toc489445125)

[Timeline Sequence for Eight Gardening Sessions 4](#_Toc489445126)

[Gardening Activity Handouts and Instructions 5](#_Toc489445127)

[Gardening Session 1: Propagation by Seed 5](#_Toc489445128)

[Gardening Session 2: Propagation by Cutting 8](#_Toc489445129)

[Gardening Session 3: Transplanting Succulents 12](#_Toc489445130)

[Gardening Session 4: Propagation by Cutting & Division 15](#_Toc489445131)

[Gardening Session 5: Simulated Harvest of Herbs 20](#_Toc489445132)

[Gardening Session 6: Propagation by Seed for Microgreens 23](#_Toc489445133)

[Gardening Session 7: Transplanting Herbs and Lettuce 26](#_Toc489445134)

[Gardening Session 8: Simulated Harvest of Florida Vegetables & Microgreens 29](#_Toc489445135)

[Appendix of Plant Latin Names Per Session 31](#_Toc489445136)

[Glossary of Key Terms 33](#_Toc489445137)

**Cover illustration by Raymond Odeh**

# Welcome to the Study

First, allow our research team to welcome you to the Gardening and Arts Study. We value your time and participation in this research, and if at any time you have questions or concerns regarding your participation in the study, feel free to contact Study Coordinator Raymond Odeh or Principal Investigator Charles Guy. Our contact information is below.

Now, let us provide you with some guidelines regarding your participation in this study:

- Please refrain from engaging in gardening or art activities including visiting botanical gardens and art museums outside of the study sessions
- Please refrain from using the Internet to find and access information about gardening or arts activities during the time following the orientation and until after the final wrap-up session
- Please do not walk through the grounds at Wilmot Gardens outside of study sessions
- You may use the (*Florida Gardener’s Handbook*) during the study as a resource and reference to find an answer to a question you may have, but avoid doing extensive reading during the time from the orientation and until after the wrap-up session
- Please do not take any photographs or make any recordings that may reveal the identity of any study subject as this will result in a protocol deviation that must be reported to the IRB
- Please inform the Study Coordinator, Raymond Odeh or the Principal Investigator, Charles Guy, if you experience any major life event that changes your daily life during the course of the study
- If you miss more than 2 of the 8 gardening sessions, you may be withdrawn from the study, it is not possible to offer a make-up session under the approved procedures of the study

This manual is intended to help you navigate each session and provides you with additional resources if you choose to continue engaging in gardening or arts activities after your active participation in the study is completed. Complementing this manual, we have provided you with a reference book, *Florida Gardener’s Handbook* to serve as a supplement to the information provided in this manual.

# Contact Information

Study Coordinator| Raymond Odeh (352)273-4525 [rodeh@ufl.edu](mailto:rodeh@ufl.edu)

*Mr. Odeh, graduate student, working on a Master of Science in Horticulture with a degree from the University of Florida specialized in Landscape and Nursery Horticulture can answer questions regarding session details and research protocol.*

Principal Investigator| Charles Guy (352) 273-4528 [clguy@ufl.edu](mailto:clguy@ufl.edu)

*Dr. Guy, Assistant Chair and Professor of Plant Physiology and Biochemistry in the Department of Environmental Horticulture with research interests in people-plant interactions and the biochemical and molecular responses of plants to unfavorable temperature conditions can answer any questions about this study.*

# Gardening Sessions Information

Mondays/Wednesdays or Tuesdays/Thursdays at 5:30 PM to 6:30 PM

The Greenhouse at Wilmot Gardens

Corner of Gale Lemerand Drive and Mowry Road, just North of Archer Road

Please park in available parking in one of the three locations circled below:

**
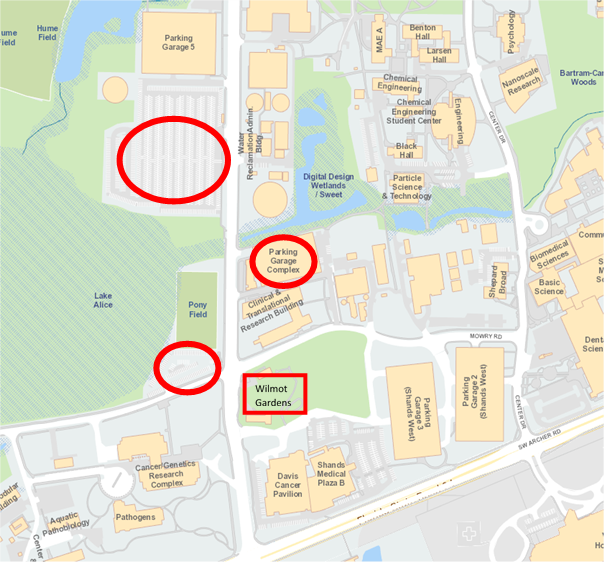
**

Mowry Road

Gale Lemerand Drive

**N**

Modified from online UF campus map <https://campusmap.ufl.edu/#/>. “© OpenStreetMap contributors”, <https://www.openstreetmap.org/copyright> (CC BY-SA 2.0)

# Timeline Sequence for Eight Gardening Sessions

| Timeline | Session Component |
| --- | --- |
| 0 min | Arrival and Sign-in/Pick up Name Tag |
| 0-10 min | Individuals Review Previous Activities (i.e. plant growth)/Instructors Greet Individuals and Assemble Group/Heart Rate and Blood Pressure Measurements Recorded |
| 10-20 min | Educational Module/Introduction to Activity/Demonstration of Activity as Warranted (booklet, instruction sheet, resource material handouts) |
| 20-50 min | Gardening Activity, Questions |
| 50-70 min | Clean-up/Heart Rate and Blood Pressure Measurement Recorded/  POMS and PSS Assessments (Even-numbered Sessions)/  BDI (Sessions 4 and 8)/  Departure |

# Gardening Activity Handouts and Instructions

## **Gardening Session 1:** Activity, Goals, Materials, Supplies and Instructions for Exercise

| **Session 1** | **PROPAGATION:** Planting Seeds of Herbs and Scented Plants e.g. basil, chives, dill, oregano, rosemary, and thyme (seedlings can be used later for Session 7) |
| --- | --- |

**Plant Propagation by Seed**

As everyone knows, seeds are an important way that plants reproduce themselves for the next generation. Seeds come in a wide variety of shapes and forms, in different colors and sizes and with different surface textures. Equally variable are the conditions that different types of seeds require to start the process of germination and continue with the growth and development of a seedling. Some seeds can germinate right away when placed in a favorable soil medium with adequate moisture and temperature, while others are blocked from immediate germination by an internal mechanism known as dormancy. The function of seed dormancy is an evolutionary informed strategy to favor germination when environmental conditions are likely to be most optimum for seedling survival and reproductive success.

This exercise will introduce you to the seeds of several herbs and scented plants that can be a starting point to make a home herb garden. You will be able to observe the germination process and the growth and development of the young seedlings, and see some of the variation that exists in the seed biology of different types of plants.

**Educational Purpose:** This exercise will highlight a group of plants widely used in culinary practices and prized for their sensory stimulating attributes. Instruction will explain the role and significance of seeds in plant propagation, sexual reproduction, and the development of a plant from a seed. Session activities will also highlight variation in plant seed morphology, size, shape and tactile characteristics, sowing methods, and rates of germination for different species. Participants will learn how to sow seeds and what materials are needed to begin growing herbs and scented plants for use at home.

**Social Interactions:** Participants will interact with session leaders and other participants. In this session, participants will be asked to share their first name with the group. The mechanism for interactions will include questions and comments between and among leaders and participants. Participants can be asked to share their favorite herbs, scented plants, and recipes with the group. Participants can be asked to pass seeds to other participants who would like to plant those varieties. Participants can demonstrate and/or observe planting techniques of other members of the group.

**Goals/Benefits:** Facilitate participant-plant interactions, increase knowledge; stimulate cognitive abilities and skills; restore directed attention; enhance motor skills (hand-eye coordination); meet new people; experience individual and group interactions; and enjoy activity.

**Materials and Supplies:**

- Soil Mix SunGro an all-purpose growing mix made with Canadian sphagnum peat moss, perlite, vermiculite, dolomitic limestone, and a wetting agent. It is suitable for a wide variety of crops especially for those requiring good drainage and aeration;
- 72-Cell Seed Pack Insert; 10” x 20” Seed Flat Tray; Plastic Dome for 10” x 20” Tray;
- Seeds Ready for Planting: basil, borage, chives, dill, oregano, rosemary and thyme or other herb plant seeds as may be available;
- Plastic Plant, Flower and Nursery Garden Label Marker Sticks 60x100mm;
- Black Sharpie Permanent Marker
- Tap water for soil mix wetting and hydration

**Considerations for Germinating Herb or Scented Plant Seeds:**

- Buy only fresh quality seed from a reliable source.
- Germinating herb seeds requires good light, quality growing medium, proper amount of moisture, the right temperature, good air circulation, and adequate gardening attention and care.
- Some herbs are more difficult to start from seeds than others are. For this reason, many types of herbs are not grown from seeds, but from vegetative cuttings. We will learn about making cuttings of herbs and scented plants in Session 2.
- Germination container: Almost anything that will hold soil and allow excess water to drain away can be used for germinating herb seeds.

**Instructions for Growing Herbs and Scented Plants from Seed:**

1. We will use standard garden supply trays and cell-pack inserts for our seeding exercise. (Note: Our containers will be unused, but should you in the future reuse containers, consider removing plant disease organisms by first rinsing the container in a mixture of 10% chlorine bleach and water, let set for at least 10 minutes, followed by rinsing with water to remove the bleach. Allow the containers to dry before using.)
2. Break apart any soil clumps to ensure soil is of fine texture, then fill the cell-pack container to the top with the soil mix.

(Note: Do not compact the soil in the container.)

1. Wet down the soil in the container and let it sit for a few minutes to hydrate the soil.
2. While you are waiting, take some label sticks and write down the names of the seeds you are planting. On your label sticks, include the common name of the plant, the date you planted the seed, and your name; this will allow you to track the progress of your seeds.
3. Planting seeds at the proper depth is very important for best germination. The amount of growing medium you place on top of your seeds is partially determined by their size. A good general rule for planting seeds is to cover them with enough soil for 1-3 times their size.

(Note: For best results, follow the directions on the seed packet or consult a reliable gardening book for proper planting requirements.)

1. Place 2-3 seeds in each cell and cover with appropriate amount of soil mix.
2. To keep the seeds from drying out, bottom water your seeds by placing the containers in a flat tray of water with about ¼” of water. Leave containers in the tray of water until the soil at the top is moist.
3. Cover your containers with a clear plastic dome or you can use plastic wrap to create high humidity to help conserve moisture during germination. To avoid too much humidity remove the dome every other day for 30 minutes to evaporate excessive moisture and allow for some fresh air. Remove the plastic dome completely once the seedlings begin growing and have developed a few leaves.

(Note: Horticultural professionals often use mist houses, fog houses or humidity chambers to maintain high relative humidity or moisture around the seeds. We may place your seed trays in the Fifield Hall Plant Science Greenhouse Complex mist house).

1. Herb seeds need as much as 14 to 16 hours of light per day in order to germinate and develop properly.
2. Monitor the germination process during your return for each session in the program.

*We will cover transplanting your seedlings in Session 7.

**Resources:**

- “Florida Gardener’s Handbook” by MacCubbin, Tasker, Bowden and Lamp’l, Cool Spring Press, Minneapolis, MN. (2012) pgs. 85, 87, 88, 90-92, 93
- *Hartmann & Kester’s Plant Propagation* by Davies, Geneve, and Wilson. Prentice Hall, Upper Saddle River, NJ. (2017). 9th ed. Chapters 4-8.
- The Herb Society of America: <http://herbsociety.org/>

## Gardening Session 2: Activity, Goals, Materials, Supplies and Instructions for Exercise

| Session 2 | PROPAGATION: Cuttings of Herbs and Scented Plants e.g. tarragon, Cuban oregano, lavender, mint, rosemary, scented geraniums, thyme, pineapple, basil, ginger, Artemisia, and sweet potato (rooted plantlets may be used for container plantings in Session 7) |
| --- | --- |

**Plant Propagation by Cuttings**

Unlike people, many plants have the ability to regenerate vital body parts after amputation, because of a phenomenon called pluripotency. Pluripotent cells can form or become any of the cell types that make up the body; embryonic stem cells are considered pluripotent. Plants have stem cells? Plants grow at specific sites known as meristems where cell division can occur and give rise to new cells and new plant parts. Meristems contain the plant version of stem cells that can make cells of the same type or other types of cells. Apical (meaning apex or tip) meristems give rise to primary shoot and root elongation and growth. Lateral meristems are responsible for horizontal growth, for example the branches on the main stem or trunk and lateral roots which would be similar to side braches on the main stem. For many plants, the presence of apical meristems for growth will often suppress the growth of lateral meristems. When the apical meristem is removed, the suppression of lateral meristems can be relieved resulting in growth processes in the lateral meristems. Wounding can also stimulate the activation and growth of new meristems, a process involving a callus phase (a growing mass of unorganized parenchyma cells,) which can give rise to meristematic cells with the appropriate environmental signals i.e. light, relative humidity and temperature, sometimes referred to as wound periderm. Callus can play an important role in regeneration of a lost body part of a plant. This ability forms the important basis for the common practice of plant propagation using cuttings from an intact plant. A cutting can be a shoot, a node, a leaf with a petiole, a leaf without a petiole, a leaflet or part of a leaf.

Activation, growth and development of meristems are hormonally controlled within the plant. In our exercise, we will stimulate the growth of meristems that lead to the formation of roots at the base of the cuttings. We will use an artificial plant hormone mixed in a talc power to coat the cut end of the cuttings to promote the growth of new roots. Some plants species do not need supplemental hormones for the meristems to start growing new roots; however, some species will not root from cuttings without the use of plant hormones, such as the commonly used hormone auxin.

In today’s exercise, we will demonstrate making cuttings from a number of herb and scented plants. The cuttings will be used to generate new intact plants, thus turning one plant into several. This is a good way one can increase the numbers of a given plant with desirable traits. For example, cuttings are widely used in commercial horticulture to generate thousands or even tens of millions of identical plants starting from a single plant.

**Educational Purpose:** This exercise will again highlight a group of plants widely used in culinary practices and prized for their sensory stimulating attributes. Instruction will explain and demonstrate how many herbaceous plants can be cloned or asexually propagated implementing cutting propagation with vegetative parts of the plant such as stems, branches or leaves and division of ramets (a group of genetically identical individuals that have grown in a given location, all originating vegetatively, not sexually, from a single ancestor). Session activities will also highlight how these asexual forms of propagation provide advantages or disadvantages over seed-based, sexual propagation methods. Participants will learn how to make vegetative cuttings and divide ramets to create new individual plants, and what materials are needed to begin growing asexually propagated herbs and scented plants for use at home. Participants will also be prompted to touch and smell the plants and comment on the sensory aspects of the plants.

**Social Interactions:** Participants will interact with session leaders and other participants as appropriate. The mechanism for interactions will include questions and comments between leaders and participants. Participants can be asked to share their favorite herbs, scented plants, and recipes with the group. Participants can also be asked about some of their favorite scents in either their homes or in the landscape, and compare current preferences to their childhood preferences. Participants can demonstrate and/or observe planting techniques of other members of the group.

**Goals/Benefits:** Facilitate participant-plant interactions, increase knowledge; stimulate cognitive abilities and skills; enhance motor skills (hand-eye coordination); trigger sensory stimulation; promote social interactions; stimulate memory and reminiscing; and meet new people; experience individual and group interactions; and enjoy activity.

**Materials and Supplies:**

- Soil Mix, SunGro is an all-purpose growing mix made with Canadian sphagnum peat moss, perlite, vermiculite, dolomitic limestone, and a wetting agent. It is suitable for a wide variety of crops especially for those requiring good drainage and aeration;
- Stock plants to take cuttings from: Artemisia, lavender, mint, rosemary, scented geraniums, Cuban oregano, and thyme or others as available;
- 72-Cell Seed Pack Insert; 10” x 20” Seed Flat Tray; Clear Plastic Covers
- Soil mixing tray
- Hand-held Clippers with Sharp Cutting Surfaces
- Rooting Hormone Powder
- Medline Souffle 0.75 Oz Disposable Paper Cups
- Plastic Plant, Flower and Nursery Garden Label Marker Sticks 60x100 mm;
- Black Sharpie Permanent Marker
- Isopropyl Alcohol
- Cotton Swabs or Paper Towels
- Water

**Considerations for Propagating Herbs or Scented Plants from Cuttings:**

- Use clean stock or parent plant that is disease free.
- Herb cuttings require good light, quality growing medium, proper amount of moisture, the right temperature, good air circulation, and good gardening attention and care.
- Cuttings should be kept moist and not dry out during the initial stages when forming roots.
- Almost anything that will hold soil and allow excess water to drain away can be used for rooting cuttings.

**Instructions for Propagating Herbs and Scented Plants from Cuttings:**

1. We will use standard garden supply trays and cell-pack inserts for our cutting exercise.

(Note: Our containers will be unused, but should you in the future reuse containers, consider removing plant disease organisms by first rinsing the container in a mixture of 10% chlorine bleach and water, let set for at least 10 minutes, followed by rinsing with water to remove the bleach. Allow the containers to dry before using.)

1. Gather some soil and place it in a mixing container. Separate any clumps of soil and add water to the soil until moistened but not over saturated.

(Note: To avoid over saturation of soil, add just enough water so that soil will form a ball in your hand without falling apart.)

1. Fill the cell-pack container to the top with the soil mix.
2. Now take your clippers in hand, wipe the cutting surfaces with a cotton swab that is saturated with isopropyl alcohol to disinfect the surface. Let air dry until alcohol has evaporated.
3. Identify a good place on the stock plant to take a cutting. Your cutting should be about 3-4 inches long and contain several nodes on the cutting.
4. After taking the cutting, strip the bottom half of the cutting of all of its leaves. Only a few leaves should be left at the top of the plant. If leaves are large, consider cutting all leaves in half, this reduces evapotranspiration and results in greater rooting success.
5. Place the tip of the cutting in water and then in your rooting hormone. Tap off excess hormone powder; only a small amount of rooting hormone is needed.

(Note: Whenever working with rooting hormone, place powder into a separate container to prevent contamination of the stock container of rooting hormone)

1. Place one cutting into each cell in the cell-pack tray. Continue this until all the cells are full or you reach the desired amount of cuttings.
2. Take label sticks and write down the names of the plants you are planting. On your label sticks include the common name of the plant, the date you planted the cutting, and your name; this will allow you to track the progress of your cuttings. Insert these into the proper place in the tray.
3. To keep the cuttings from drying out, water the cuttings regularly or place under a misting system to keep the area around the cutting moist; this increases relative humidity.
4. In later sessions, you can check to see if your cuttings are producing roots by gently pulling on the cutting. If it pulls free easily, it has not produced roots. If it resists your pull, it has begun to form roots. Plants will start to form roots in the following weeks.

**Resources:**

- “Florida Gardener’s Handbook” by MacCubbin, Tasker, Bowden and Lamp’l, Cool Spring Press, Minneapolis, MN. (2012) pgs. 22, 89, 91, 92
- *Hartmann & Kester’s Plant Propagation* by Davies, Geneve, and Wilson. Prentice Hall, Upper Saddle River, NJ. (2017). 9th ed. Chapters 10-12.
- “Herbs in the Florida Garden” by James M. Stephens. CIR570, one of a series of the Horticultural Sciences Department, UF/IFAS Extension. Original publication date March 1994. Revised March 1998. Reviewed March 2014. Visit the EDIS website at <http://edis.ifas.ufl.edu/vh020>

## Gardening Session 3: Activity, Goals, Materials, Supplies and Instructions for Exercise

| Session 3 | TRANSPLANTING: Container Garden Succulent Collection Planting e.g. Aloe spp., *Echeveria* spp., Jade Plant, *Kalanchoe* spp., Panda Toes, and *Sedum* spp.) |
| --- | --- |

**Succulent Plants**

Succulent refers to a broad category of mostly xeriphytic plants, including cacti, that have thick fleshy leaves or stems that serve as water storage organs in arid conditions. Succulents are found worldwide, and include a number of familiar plants such as the medicine plant (*Aloe barbadensis*), the jade plant (*Crassula arborescens*), Kalanchoes and Sedums (*Sedum* sp.) that are commonly found in succulent or perennial gardens.

Many succulents are well adapted to living indoors in homes where the relative humidity is often much lower than outside in Florida. They require much less amounts of water and fertilizer than other types of plants. Most commonly grown succulents prefer warm temperatures and are not able to tolerate freezing. Succulents typically need abundant sunlight or light to remain healthy. At home, succulents should be located in bright, sunny spaces often in a window that faces east, south or west. Low natural light can be supplemented by artificial lighting. Cool white fluorescent bulbs or a combination of daylight and natural white fluorescent light can give good results. Position lights about 6-12 inches above the plants, and keep them on for 14-16 hours each day or as necessary.

Succulents are often grouped together in container or dish gardens. This can be an extremely attractive method of display, as long as several precautions are taken. Choose plants that are compatible in rate of growth so that one or two plants do not outgrow the rest. Plants should have similar water requirements. Generally speaking, most cacti need less water than do other succulents. Since shallow dishes seldom have drain holes, it is essential that the plants are not overwatered.

Most succulents and cacti grow in open, well-drained sandy soils. These conditions should be duplicated for your indoor container garden whenever possible. A mix of one part potting soil and one part coarse sand (builders sand) is usually porous enough for the health of succulents. A good test is to moisten the mixture and squeeze it in your hand. On release, the soil should fall apart. Both pot and growing medium should be sterile. Ideally, these plants should be grown in pots with drainage holes, because excess water trapped in the soil will result in rot and decay in a very short time.

**Educational Purpose:** This exercise will highlight a group of plants widely used in ornamental horticulture and valued for their fascinating morphological diversity and vegetative attributes. Instruction will explain and demonstrate how transplanting of young and rooted plants can be used to create an aesthetically pleasing arrangement for display and enjoyment. Key principles that maximize transplant success and some design elements will be presented.

**Social Interactions:** Participants will interact with session leaders and other participants as appropriate. The mechanism for interactions will include questions and comments between leaders and participants. Participants can be asked to share their favorite succulent plants with the group. Participants can be asked to pick their favorite plant out of the group and give reasons for why they may enjoy it. Participants can be asked to comment on why these plants have a different morphology than other plants, and they will be taught about xerophytic plants.

**Goals/Benefits:** Facilitate participant-plant interactions, increase knowledge; stimulate cognitive abilities and skills; enhance motor skills (hand-eye coordination); promote social interactions; trigger sensory stimulation; become familiar with other group members; experience individual and group interactions; and enjoy activity.

**Materials and Supplies:**

- Succulent Mix (composed of 50% sand); coarse builder’s sand and Soil Mix, SunGro an all-purpose growing mix made with Canadian sphagnum peat moss, perlite, vermiculite, dolomitic limestone, and a wetting agent. Colored sand small rocks can be used for topdressing the medium to add aesthetic and decorative value.
- Large container for transplanting young plants
- Transplants of succulents: aloe, Echeveria, jade plant, Kalanchoe, panda toes, sedum
- Colored coarse sand (optional)
- Small rocks for placing on the top of the soil around the plants
- Plastic Plant, Flower and Nursery Garden Label Marker Sticks 60x100mm;
- Black Sharpie Permanent Marker
- Water

**Considerations for Transplanting Succulents:**

- Containers with good drainage should be used
- Growing media should allow for drainage through the profile of the container
- Succulents planted in the same container should be matched in their water requirements
- Consider the growth habits of each succulent species for a compatible container garden

**Instructions for Transplanting Succulents into a Container Garden:**

1. We will use containers that will be appropriate for holding succulent plants and have good drainage on the bottom of the container.

(Note: Some containers have drain holes that have not been removed yet. Ensure that the plastic holes are popped out before use.)

1. Start by taking some of the succulent potting mix and placing it into a soil-mixing container. Add water to the soil until moistened but not over saturated.
2. Place the soil mix into your container.
3. Choose plants for your container and think where you will want to place them in the container. A good guide for designing container gardens is to use the “thrill, fill, spill” method. So perhaps choose plants that will fit these criteria.
4. Make holes in the media where you will plant your succulent transplants. Ensure that the roots have enough room when placed into their holes. Do not crush the root ball into a space where it does not fit. After placing a plant into a transplant hole, gently fill in the soil where there is remaining space.

(Note: Some succulents have leaves and stems that easily break off, so this can be a delicate process. If leaves or stems break off, consider leaving them in the container to root as cuttings.)

1. Repeat for all the plants until you have placed in all the plants that you desire.
2. Make sure that your plants have room to grow in the container. Do not overcrowd the container.
3. Place rocks around the top of the soil in order to add to the aesthetic appeal of the container.
4. You may want to write a label with some of the plants names on it. You will also want to label the container with your name.
5. After a few weeks, the plants will be established in the container and start to fill in all the empty spaces.
6. Make sure that you do not overwater these plants. These plants are desert plants and typically do not need much water or fertilizer. Ensure you let the soil medium dry out between watering, and place containers in a space with plenty of sun.
7. After a while, the plants may need to be transplanted to another container if this one seems to be getting overly crowded.

**Resources:**

- “Florida Gardener’s Handbook” by MacCubbin, Tasker, Bowden and Lamp’l, Cool Spring Press, Minneapolis, MN. (2012) 272 pgs. 127, 213
- “Container Gardens for Outdoor Spaces” by Gary W. Knox. #ENH1095, one of a series of the Environmental Horticulture Department, UF/IFAS Extension. Original publication date February 2008. Reviewed February, 2014. Visit the EDIS website at <http://edis.ifas.ufl.edu>.

## Gardening Session 4: Activity, Goals, Materials, Supplies and Instructions for Exercise

| **Session 4** | **PROPAGATION:** Cuttings/Division of Herbaceous Ornamentals (African violet, begonia, coleus cultivars, hosta, hoya, impatiens, pilea, prayer plant, and spider plant) |
| --- | --- |

**Plant Propagation by Cuttings and Division**

Session 2 demonstrated the value and importance of propagating desirable plants by cuttings. This session will again highlight and reinforce the use of making cuttings in plant propagation, but here the focus will be on ornamentals. For most of the plants in this exercise, we will make cuttings (stems or shoots) very similar to those made in Session 2. However, in this session cuttings using leaves and parts of leaves will also be demonstrated with African violet, begonia, and hoya.

This session will also illustrate a second form of propagation that is known as division using the hosta, prayer plant, and spider plant as examples. Some plants have growth habits that allow them to spontaneously multiply and give rise to clonal copies. Frequently new plantlets are spawned at the base of the stem of the parent plant, but in other cases, underground stems known as rhizomes (pilea) or above ground stems known as stolons (spider plant) can give rise to rooted plantlets that have their own roots and shoots while still being connected to the parent plant. When a parent plant produces clonal plantlets, the plantlets can be detached from the parent and transplanted to a new location or container. Generating new plants by division is the most successful form of propagation, because an intact plantlet is ready to begin independent growth without having to form replacement parts. The example of a parent plant producing clonal plants is the well-known spider plant. The spider plant will form many long aerial stolons that will each form a clonal plantlet at the end. Spider plants are often grown in hanging baskets allowing the long stolons to hang down with the clonal plantlets slowly growing at the ends. This is one of many survival strategies that plants use to asexually propagate themselves.

**Educational Purpose:** This exercise will highlight a group of plants widely used in ornamental horticulture and valued for their foliage, floral and vegetative attributes. Instruction will explain and demonstrate how many herbaceous ornamental plants can be cloned or asexually propagated implementing cutting propagation with vegetative parts of the plant such as a stems, branches or leaves and division methods. This session will also cover how these forms of propagation provide advantages or disadvantages over seed-based, sexual propagation methods. Participants will make vegetative cuttings, divide plants, and learn the materials necessary to begin growing asexually propagated herbaceous ornamental plants at home.

**Social Interactions:** Participants will interact with session leaders and other participants as appropriate. The mechanism for interactions will include questions and comments between leaders and participants. Participants will be asked to work in small groups to propagate the different types of plants. Participants can be asked to share their favorite ornamental flowers and plants with the group. Participants can also be asked to comment on why certain plants become popular horticultural crops while others do not.

**Goals/Benefits:** Facilitate participant-plant interactions, increase knowledge; stimulate cognitive abilities and skills (recognition, learning and concept formation); enhance motor skills (hand-eye coordination); promote social interactions; improve neuromusculoskeletal elements such as range of motion, muscle tone, strength, and postural control; enjoy activity. Participants will experience common group goals, shared accomplishments, and cooperative interactions.

**Materials and Supplies:**

- Soil Mix, SunGro an all-purpose growing mix made with Canadian sphagnum peat moss, perlite, vermiculite, dolomitic limestone, and a wetting agent. It is suitable for a wide variety of crops especially for those requiring good drainage and aeration;
- 4” and 6” and 1 gallon plastic pots (chosen based on size of cutting/division)
- Stock plants: African violet (leaf cutting), begonia (stem, leaf, or wedge cutting) , coleus (stem cutting), hoya (leaf cutting), hosta, prayer plant, and spider plant (division)
- Clippers
- Knife
- Rooting powder
- Small cups
- Isopropyl alcohol
- Cotton swabs
- Plastic Plant, Flower and Nursery Garden Label Marker Sticks 60x100 mm;
- Black Sharpie Permanent Marker
- Water

**Considerations for Propagation of Herbaceous Ornamentals:**

- Containers with good drainage should be used
- Some leaf and stem cuttings can be taken a few days prior to planting and be placed in water. Some plant species will start to develop roots while in water.
- Some plants can be propagated multiple ways. It can be fun to test out different ways and see which method works best for you.
- Leaf cuttings often take longer to develop roots than a stem cutting, so remember to be patient for this plant to take root and start producing a new plant.
- Wedge cuttings often take the longest to develop roots out of all the rooting methods described here. So remember to be patient for this plant to take root and start producing a new plant.
- Some plants produce new meristems at their base and quickly form a clump. When a clump becomes too large for a pot or if you simply would like to divide and share your plant, you can simply split the clump into separate pieces.

**Instructions for Propagation of Herbaceous Ornamentals:**

1. We will use standard garden pots for our exercise.

(Note: Our containers will be unused, but should you in the future reuse containers, consider removing plant disease organisms by first rinsing the container in a mixture of 10% chlorine bleach and water, let set for at least 10 minutes, followed by rinsing with water to remove the bleach. Allow the containers to dry before using.)

1. Gather some soil and place it in a mixing container. Separate any clumps of soil and add water to the soil until moistened, but not over saturated.

(Note: To avoid over saturation of soil, add just enough water so that soil will form a ball in your hand without falling apart.)

1. Fill the pots you plan on using with the moistened soil.

**Leaf Cutting**

1. Take your clippers and identify a healthy leaf to be cut off for propagation. Gently clip off this leaf at the petiole.
2. Place the tip of the petiole in water and then in your root hormone. Tap off excess hormone powder; only a small amount of rooting hormone is needed.

(Note: Whenever working with rooting hormone, place powder into a separate container to prevent contamination of the stock container of rooting hormone)

1. Place the leaf into a pot filled with soil. Make sure that the leaf has been pushed down into the soil and will not fall over when watered.
2. Take label sticks and write down the names of the plants you are planting. On your label sticks include the common name of the plant, the date you planted the cutting, and your name; this will allow you to track the progress of your cuttings. Insert these into the proper place in the pot.
3. To keep the cuttings from drying out, water the cuttings regularly or place under a misting system to keep the area around the cutting moist; this increases relative humidity.
4. In later sessions, you can check to see if your cuttings are producing roots by gently pulling on the cutting. If it pulls free easily, it has not produced roots. If it resists your pull, it has begun to form roots. Plants will start to form roots in the following weeks.

**Stem Cutting**

1. Take your clippers and identify a healthy place on the stock plant to take a stem cutting. Your cutting should contain three or more nodes on the stem. Having 4 to 5 is generally favorable.
2. After taking the cutting, strip the bottom of the cutting of all of its leaves. Only a few leaves should be left at the top of the plant. If the leaves are large, you can trim them to a smaller size using your clippers.
3. Place the tip of the cutting in water and then in your root hormone. Tap off excess hormone powder; only a small amount of rooting hormone is needed.

(Note: Whenever working with rooting hormone, place powder into a separate container to prevent contamination of the stock container of rooting hormone)

1. Place the cutting into a pot filled with soil. Make sure that the stem has been pushed down into the soil and will not fall over when watered.

(Note: Some cuttings root from the cut end, while others root from the node closest to the cut end; therefore, a good practice is to bury at least one basal node of the cutting)

1. Take label sticks and write down the names of the plants you are planting. On your label sticks include the common name of the plant, the date you planted the cutting, and your name; this will allow you to track the progress of your cuttings. Insert these into the proper place in the pot.
2. To keep the cuttings from drying out, water the cuttings regularly or place under a misting system to keep the area around the cutting moist; this increases relative humidity.
3. In later sessions, you can check to see if your cuttings are producing roots by gently pulling on the cutting. If it pulls free easily, it has not produced roots. If it resists your pull, it has begun to form roots. Plants will start to form roots in the following weeks.

**Wedge Cutting**

1. Take your clippers and remove a large leaf from the stock plant. You will then want to remove a “wedge” from this leaf that has a vein running through it. Roots will start to develop from this vein. There is a diagram at the bottom of this handout to show you what your wedge cutting should look like.
2. Place the bottom of the wedge cutting in water and then in your root hormone. Tap off excess hormone powder; only a small amount of rooting hormone is needed.

(Note: Whenever working with rooting hormone, place powder into a separate container to prevent contamination of the stock container of rooting hormone)

1. Push the wedge into the soil so that it is standing upright. You may need to tamp soil around the base of the leaf to ensure that it will be sturdy and not fall over.
2. Take label sticks and write down the names of the plants you are planting. On your label sticks include the common name of the plant, the date you planted the cutting, and your name; this will allow you to track the progress of your cuttings. Insert these into the proper place in the pot.
3. To keep the cuttings from drying out, water the cuttings regularly or place under a misting system to keep the area around the cutting moist; this increases relative humidity.
4. In later sessions, you can check to see if your cuttings are producing roots by gently pulling on the cutting. If it pulls free easily, it has not produced roots. If it resists your pull, it has begun to form roots. Plants will start to form roots in the following weeks.

**Division**

1. Decide how many divisions you would like to make of this plant. This will depend on the size of the plant and how many new plants you would like to have.
2. Start by removing the parent plant from the pot. Identify some gaps between the leaves where pulling apart or cutting apart may be easiest.
3. Gently pull apart a portion of the clump using your hands. If it does not break apart easily, you may need to use a knife or clippers to cut through the material.
4. It is OK to cut through roots and plant parts. Often times the plant will simply regrow new roots to replace those.
5. Place the new clump into a new pot with soil.
6. Take label sticks and write down the names of the plants you are planting. On your label sticks include the common name of the plant, the date you planted the plant, and your name; this will allow you to track the progress of your plant. Insert these into the proper place in the pot.
7. To keep the divided plants from drying out, water them regularly or place under a misting system to keep the area around the plants moist; this increases relative humidity.
8. In later sessions, you can check to see if your divided plants have established in the soil by looking for signs of new leaves or new roots from the bottom of the pot.

**Resources:**

- “Florida Gardener’s Handbook” by MacCubbin, Tasker, Bowden and Lamp’l, Cool Spring Press, Minneapolis, MN. (2012) pgs. 20, 143, 224,
- *Hartmann & Kester’s Plant Propagation* by Davies, Geneve, and Wilson. Prentice Hall, Upper Saddle River, NJ. (2017). 9th ed. Chapters 10-12.

## Gardening Session 5: Activity, Goals, Materials, Supplies and Instructions for Exercise

| Session 5 | EDUCATION/TASTING/SENSORY: Herbs and Herb Flavors (basil, chives, cilantro/coriander, dill, mint, oregano, parsley, rosemary, sage, and thyme) |
| --- | --- |

**Herbs and Plants Used in Culinary Practice**

Plants make an amazing array of chemical compounds, more so than almost any other type of organism. The extra ability of plants to make different chemical compounds beyond what is needed for basic cellular and organism function relates both to the unique biology of plants and the diversity of plants from a wide variety of environments. Unlike animals, plants are not able to move around to find food, to find mates or to escape an enemy or unfavorable environmental condition. One way they can accomplish these needs is through chemical biology. In the simplest form, many plants produce nectar to attract and reward insect pollinators coming to their flowers to spread pollen across long distances to other receptive flowers. Like the pollinators, humans can be attracted to plants because of their uniquely varied chemical constituents that we find pleasant smelling, visually attractive, tasty or in one way or another psychoactive.

Herbs belong to a loose grouping of plants that are grown for their special flavor and aromatic characteristics that can enhance our environment or modify the aroma and flavor of our foods. Botanically speaking, an herb is a seed-bearing plant that does not have a woody stem, and dies after flowering. Herb is from the Latin word *herba* which means grass or green crops. From a culinary perspective, the shoots, leaves, flowers, seeds and roots of many herbs can be used for flavoring. The flavoring is often due to a large array of volatile organic compounds and/or essential oils that the plant tissues contain. Depending on the herb and the intended use, the plant material can be used fresh or after drying and curing. In this exercise, the group will sample fresh forms of popular herbs; and taste some examples of herbs widely used in foods and beverages.

**Educational Purpose:** This exercise will further highlight a group of plants widely used in culinary practices and prized for their sensory stimulating attributes. Participants will learn about herbs, herb flavors and the plants that provide a wide range of flavorings and seasonings for culinary practice. Individuals will experience the visual, olfactory, taste and tactile qualities and characteristics of this grouping of plants. Instructors will describe how to dry herbs and how to create seasonings. Participants will experience making and sampling pesto served on a baguette.

**Social Interactions:** Participants will interact with session leaders and other participants as appropriate. The mechanism for interactions will include questions and comments between leaders and participants. Participants can be asked to share with the group their favorite herbs and scented plants, recipes, and other ways of using herbs and spices. Participants can also be asked if there is a particular herb they use often in cooking that would be a good option for them to grow at home to save money. Small groups of participants will prepare a custom-made pesto with different combinations of fresh herbs to share with other session members.

**Goals/Benefits:** Facilitate participant-plant interactions, increase knowledge; stimulate cognitive abilities and skills; enhance motor skills (hand-eye coordination); trigger sensory stimulation; promote social interactions; and enjoy activity. Experience group cooperativity and creativity in making a custom pesto.

**Materials and Supplies:**

- Fresh herbs: basil, chives, dill, mint, oregano, parsley, rosemary, sage, and thyme
- Dried herbs: basil, cilantro/coriander, dill, mint, oregano, parsley, rosemary, sage, and thyme (as available)
- Electric Kettle (for hot tea); cups, stirs, sugar, and agave nectar
- Food processor
- Parmesan cheese
- Lemons or lemon juice
- Kosher salt
- Extra virgin olive oil

**Considerations for Harvesting and Preparing Herbs:**

- To encourage branching in perennial herbs, remove the growing tips or apical meristems
- It is OK to cut perennial herbs to about half of their size
- Remove flowers for longer vegetative yields
- Review the pesticide application history before consuming herbs

**General Activities**

1. Fresh herbs will be passed around and people will be asked to smell, taste, and comment on each herb.
2. Fresh herbs will be compared to dry herbs in smell and flavor.

**Instructions on drying fresh herbs**

1. Clip herb when they are at their freshest and most flavorful. For leaf herbs, this is when the plant is producing buds, but before it has flowered.
2. Tie together your bundle of herbs. Hang in a cool, well-ventilated place. Indoor drying is more favorable than outdoor drying because flavor is better preserved.

(Note: This works well for less tender herbs such as rosemary, sage, thyme, and parsley)

1. For tender herbs (basil, oregano, tarragon, lemon balm, and mint), place the herb in small bundles in a paper bag. To prevent molding, tear holes in the bag to allow for air-circulation. Close the top of the bag with a rubber band and hang upside down. The leaves will fall off into the bottom of the bag
2. Tender herbs can also be dried using the oven. Pull leaves off the stem. Lay the leaves flat on a sheet in the oven. Turn on the oven light and leave overnight. The oven-light often produces enough heat to dry the leaves.

**Resources:**

- “Florida Gardener’s Handbook” by MacCubbin, Tasker, Bowden and Lamp’l, Cool Spring Press, Minneapolis, MN. (2012) pgs. 85, 87, 88, 90-92, 93
- “Herbs in the Florida Garden” by James M. Stephens. CIR570. Series of the Horticultural Sciences Department, UF/IFAS Extension. Original publication date March 1994. Revised March 1998. Reviewed March 2014. Visit the EDIS website at: <http://edis.ifas.ufl.edu>

## Gardening Session 6: Activity, Goals, Materials, Supplies and Instructions for Exercise

| **Session 6** | **PROPAGATION:** Planting Seeds of Fast Germinating Vegetables e.g. radish, lettuce and tomato), (Microgreens trays: arugula, garden cress, kale, radish, spinach, and Swiss chard) |
| --- | --- |

**Plant Propagation by Seeds and Microgreens**

Reviewing from Session 1, seeds are an important way that plants reproduce themselves for the next generation. Seeds come in a wide variety of shapes and forms, in different colors and sizes and with different surface textures. Equally variable are the conditions that different types of seeds require to start the process of germination, grow, and develop into a seedling. Some seeds can germinate right away when placed in a favorable soil medium with adequate moisture and temperature, while others are blocked from germination by an internal mechanism known as dormancy. None of the seeds used in this exercise will be inhibited from germinating because of dormancy.

This exercise will introduce you to the seeds of vegetable plants that can be a starting point to make a single container or potted plant, start a vegetable garden or grow your own microgreens. You will be able to observe the germination process and the growth and development of the young seedlings, and see some of the variation that exists in the various types of plants.

Microgreens are a relatively recent and trendy concept in vegetable production that has stimulated interest in the popular press. One example is an article at WebMD entitled: “Tiny Microgreens Packed with Nutrients,” (<http://www.webmd.com/diet/news/20120831/tiny-microgreens-packed-nutrients>). Many recipes can now be found incorporating the use of microgreens. Microgreens are young immature vegetable seedlings that are harvested at the first true leaf stage, and they are used for color and to add texture and flavor to salads and other dishes. Microgreens can be differentiated from baby greens by the fact that microgreens are younger and usually smaller than baby greens. The distinction is not characterized by a specific botanical growth stage. The flavor and aroma of microgreens are going to differ from those of their mature vegetable forms that we know. This illustrates how plant chemical composition can change as plants develop, mature and even senesce.

With the microgreens, we might also be able to observe how planting density influences the development of young seedlings. You may find it surprising that plants can send and receive chemical signals from one another, but they do this all the time! Plants can also sense when they have neighbors and how close its neighbors might be. We will be planting the seeds for microgreens very densely and we should be able to observe how crowding influences the growth of the young seedlings. In Session 8, we plan to harvest the microgreens and sample them individually and in a salad.

**Educational Purpose:** This exercise will highlight a group of very important vegetable food crops valued for their nutritional attributes. Instruction will further explain the role and significance of seeds in plant reproduction, propagation, and the development of a plant from a seed. This activity will illustrate variation in plant seed morphology, size, shape and tactile characteristics, demonstrate sowing methods, and observe the rate of germination of different species. Participants will learn how to sow seeds and what materials are needed to begin growing vegetables for use at home. This exercise in conjunction with Session 10 will also demonstrate the rapid production of fresh microgreens for home use.

**Social Interactions:** Participants will interact with session leaders and other participants as appropriate. The mechanism for interactions will include questions and comments between leaders and participants. Participants can be asked to share their favorite microgreens, vegetable plants and recipes with the group. Participants can give and receive advice on how to sow the different types of seeds, and share life experiences and memories of growing and consuming vegetables and salads.

**Goals/Benefits:** Facilitate participant-plant interactions, increase knowledge; stimulate cognitive abilities and skills; enhance motor skills (hand-eye coordination); promote social interactions; and enjoy activity.

**Materials and Supplies:**

- Soil Mix, SunGro an all-purpose growing mix made with Canadian sphagnum peat moss, perlite, vermiculite, dolomitic limestone, and a wetting agent. It is suitable for a wide variety of crops especially for those requiring good drainage and aeration;
- Greens growing tray: 10” x 20”, Seed Flat Tray with holes, Plastic Dome for 10” x 20” Tray
- 4” plastic pots
- Seeds for microgreens and vegetables
- Soil mixing tray
- Plastic Plant, Flower and Nursery Garden Label Marker Sticks 60x100 mm;
- Black Sharpie Permanent Marker
- Water

**Considerations for Growing Vegetables and Microgreens from Seed:**

- Appropriate seeding density should be achieved. For smaller seeded varieties, aim for 10-12 seeds per sq. in. For larger seeded varieties, aim for 6-8 seeds per sq. in.
- If not seeding enough, you may not produce a desirable yield
- If seeding too much, you increase risk of disease

**Instructions for Growing Vegetables and Microgreens from Seed:**

1. We will use containers that will be appropriate for holding microgreens and vegetable sprouts with good drainage from the bottom of the container.
2. In the soil-mixing tray, place some soil and break apart any soil clumps to ensure soil mix is of fine texture.
3. Fill your microgreen tray with dry soil until it reaches 1” below the top of the tray.

(Note: Do not compact the soil in the container.)

1. Wet down the soil in the tray and let it sit for a few minutes to hydrate the soil.
2. While you are waiting, take some label sticks and write down the names of the plants you are planting. On your label sticks, include the common name of the plant, the date you planted the seed, and your name; this will allow you to track the progress of your seeds.

**Planting Microgreens**

1. Divide your tray into different sections where you will spread your different types of seeds and place label sticks in each section.
2. Start to spread your seeds on the top of the soil and try to spread evenly (this is easier said than done). These plants can grow very close together, aim for about 10 seeds per sq. in.
3. Sprinkle a small layer of soil on the top of the seeds, about ¼” thick.
4. Gently water the seeds.
5. Plants should begin to germinate in a week. Once the first true leaves are opened, the microgreens can be harvested. Time elapsed from seeding to harvest varies between 7-21 days, depending on the seed.

**Resources:**

- “Florida Gardener’s Handbook” by MacCubbin, Tasker, Bowden and Lamp’l, Cool Spring Press, Minneapolis, MN. (2012) 88, 92, 94-95
- *Hartmann & Kester’s Plant Propagation* by Davies, Geneve, and Wilson. Prentice Hall, Upper Saddle River, NJ. (2017). 9th ed. Chapters 4-8.
- “Microgreens: A New Specialty Crop” by Danielle Treadwell, Robert C. Hochmuth, and Linda Landrum. #HS1164. Series of the Horticultural Sciences Department, UF/IFAS Extension. Original publication date April 2010. Revised July 2013. Reviewed October 2016. Visit the EDIS website at: <http://edis.ifas.ufl.edu>

## Gardening Session 7: Activity, Goals, Materials, Supplies and Instructions for Exercise

| **Session 7** | **TRANSPLANTING:** Container Herb and Lettuce Salad Bowl Planting (seeds and cuttings from Sessions 1 and 2 can be used) |
| --- | --- |

**Transplanting**

Long before transplantation became possible in medicine, botanists and horticulturists were transplanting plants that they fancied. The word “transplant” comes from Latin *trans* which means across and *plantare* which means to plant. It has always been part of our nature to take plants that we like or need wherever we go. Sometimes that means taking a fruit, seed, cutting or an entire plant. Our expanding understanding of plant biology and our large machines have made it possible in recent times to successfully transplant fully-grown large trees. Perhaps you have seen trucks on the highways carrying large palm trees or other trees to be transplanted. In fact, most of the large palms on campus were transplanted from somewhere off campus. Many of the plants in Wilmot Gardens are transplants.

This exercise will demonstrate how to transplant young, small plants to make a containerized herb and lettuce bowl that can provide greenery and a fresh source of herbs and lettuce for culinary purposes in the home. Some of the herb seeds germinated from Session 1 and herb cuttings rooted from Session 2 should be ready for transplanting into our containers. Some keys to successful transplanting include: 1, preparing the soil at the site where the transplant will be planted; 2, carefully removing the plant from its original site ensuring that damage to the root system is minimized; 3, planting to the proper depth (not too deep or too shallow); and 4, watering and caring for the plant post-transplant. Like people, being transplanted can also be stressful to a plant. Plants can and often do experience transplant shock, but with proper attention and care it can be minimized and the success rate for plant survival and healthy growth can be maximized.

The beauty of an herb and lettuce or salad bowl is that it does not require a lot of supplies or an outside plot of land, instead requiring little space and in a few weeks, you can raise a small edible garden. Provided you have a suitable source of light, your salad bowl can be grown indoors. Apartment patios, back porches screened in enclosures with good sun exposure (at least for a few hours a day direct light) can be a perfect place for your container garden.

A salad bowl can provide fresh leafy vegetables and herbs with the first crop ready for harvest in a few weeks, and the regrowth can perhaps be harvested multiple times. Most leafy vegetables can be harvested down almost to the ground level and will still re-grow. The plants can be planted much closer together than would be typical, because harvesting does not require waiting for mature, full-size plants.

**Educational Purpose:** This exercise will further highlight a group of plants widely used in sensory stimulation and culinary practices. Instruction will explain and demonstrate how transplanting of young and rooted plants can be used to create a small sensory garden that can be aesthetically pleasing, serve as a source of sensory stimulation, and provide flavoring and nutrition for culinary uses. Key principles that maximize transplant success will be reinforced, and some elements of design will be presented.

**Social Interactions:** Participants will interact with session leaders and other participants as appropriate. The mechanism for interactions will include questions and comments between leaders and participants. Participants can be asked to share their favorite herbs, scented plants and recipes with the group.

**Goals/Benefits:** Facilitate participant-plant interactions, increase knowledge; stimulate cognitive abilities and skills; enhance motor skills (hand-eye coordination); promote social interactions; increase sense of pride and self-expression; and enjoy activity.

**Materials and Supplies**

- Soil Mix, SunGro an all-purpose growing mix made with Canadian sphagnum peat moss, perlite, vermiculite, dolomitic limestone, and a wetting agent. It is suitable for a wide variety of crops especially for those requiring good drainage and aeration
- Slow Release Fertilizer (Scott’s Osmocote 14-14-14 or equivalent)
- Herb and lettuce transplants: basil, chives, dill, mint, oregano, and thyme
- Larger container for transplanting into young plants
- Plastic Plant, Flower and Nursery Garden Label Marker Sticks 60x100 mm;
- Black Sharpie Permanent Marker
- Water

**Considerations for Transplanting Herbs/Lettuce into a Container Garden:**

- Many leafy vegetables can be harvested down almost to the ground level and will still re-grow.
- Plants in a salad bowl can be planted much closer together than would be typical, because harvesting does not require waiting for mature, full-size plants
- When transplanting large plants with greater overall leaf surface area, consider reducing leaf area and number by cutting leaves in half or removing older leaves completely
- Herbs and lettuce require quite a bit of water and full to partial sun in order to grow large.
- Watering herbs less once the plants are established and ready to use in cooking is a good idea as it stresses the plants, causing them to produce more of the compounds that are responsible for flavor and smell.
- Whenever you would like to eat a salad, simply harvest the greens from the container. Use a pair of scissors to cut the greens about 1” above the base. Leaving this 1” will let the plants continue to grow so you can get a few more harvests out of the greens.

**Instructions for Transplanting Herbs/Lettuce into a Container Garden:**

1. We will use containers that will be appropriate for holding herbs and lettuce with good drainage from the bottom of the container.
2. Gather some soil and place it in a mixing container. Separate any clumps of soil and add water to the soil until moistened but not over saturated.

(Note: To avoid over saturation of soil, add just enough water so that soil will form a ball in your hand without falling apart.)

1. Place the soil mix into your container.
2. Choose plants for your container and think where you will want to place them in the container. A good guide for designing container gardens is to use the “thrill, fill, spill” method. So perhaps choose plants that will fit these criteria.
3. Make holes in the media where you will plant your transplants. Make sure that the roots have enough room when placed into their holes. Do not crush the root ball into a space where it does not fit. Place a small amount of slow release fertilizer (¼ of the scoop) in the planting hole and cover with a layer of soil.
4. Place a plant into a transplant hole; gently fill in the soil where there is remaining space.
5. Repeat for all the plants until you have placed in all the plants that you desire.
6. Make sure that your plants have room to grow in the container. Do not overcrowd the container.
7. You may want to write a label with some of the plant names on it. You will also want to label the container with your name.
8. After a few weeks, the plants will be established in the container and start to fill in all the empty spaces.
9. After several months, the herb plants may need to be transplanted to another container if this one seems to be getting crowded. Many of the herbs are annual plants and will not survive a freeze. So either protect your container by covering it in the cold or simply replant with cool season herbs in the winter.
10. After a few harvests, the lettuce greens may start to decline or look bad. This means that they are done producing and can be composted or thrown away. They may also bolt if you leave them growing for too long. When the plant bolts, the leaves will become bitter, so leaves should be harvested before then.

**Resources:**

- “Florida Gardener’s Handbook” by MacCubbin, Tasker, Bowden and Lamp’l, Cool Spring Press, Minneapolis, MN. (2012) pgs. 85, 87, 88, 90-92, 93
- “Starting The Garden With Transplants” by James M. Stephens. #HS507, one of a series of the Horticultural Sciences Department, Florida Cooperative Extension Service, Institute of Food and Agricultural Sciences, University of Florida. Original publication date April 1994. Revised April 1994. Reviewed May 2011. Visit the EDIS website at <http://edis.ifas.ufl.edu>.

## Gardening Session 8: Activity, Goals, Materials, Supplies and Instructions for Exercise

| **Session 8** | **EDUCATION/TASTING/SENSORY:** Florida Vegetables and Flavors (bell pepper, broccoli, carrot, celery, collards, cucumber, lettuce, and onion); Microgreen salad tasting (arugula, garden cress, kale, radish, spinach, and Swiss chard). |
| --- | --- |

**Florida Vegetables**

Florida is a major vegetable producing state. According to the USDA (Vegetables, NASS, USDA, 2015), in 2015 Florida ranked second in the nation in vegetable production in terms of acreage harvested and gross sales. The farm value of vegetables grown in Florida was estimated to be more than 1.0 billion dollars. Florida farms commercially grow more than 40 different vegetable crops, with harvests largely occurring in fall, winter and spring. In Florida, tomatoes account for about $3 out of every $10 dollars in production value. The top ten vegetable crops according to market value in Florida are in order: tomato, strawberry, bell pepper, sweet corn, potatoes, watermelon, snap beans, cucumber, cabbage, and squash.


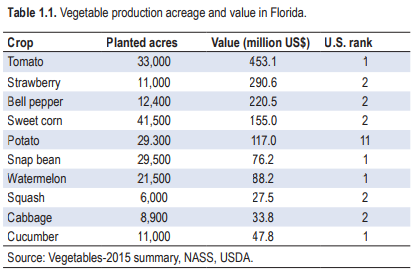


Perhaps one finds it surprising that strawberry and watermelon are included as a vegetable by the USDA. Many would consider strawberry to be a fruit, but then why is it listed as a vegetable? Well the word vegetable is not a botanical or scientific word, and there lies the source of confusion as to what is and what is not a vegetable. The meaning of the term *vegetable* is rather arbitrary and subjective whereas the term *fruit* is a botanical and scientific word with a specific meaning “the mature ovary of a plant.” What is an ovary of a plant? An ovary is the enlarged basal portion of the pistil where the embryo and seeds form and includes the fleshy tissues that surround the embryo or seed. Apples, pears, oranges and blackberries are fruits. A fruit is produced only from a flower, yet while a tomato is botanically speaking a fruit; it is also non-scientifically considered a vegetable. One definition of vegetable would be the edible portion of an herbaceous plant including leaves (lettuce), stems (celery), roots (carrot), tubers (potato), bulbs (onion) and flowers and floral stalks (broccoli) or fruits. Other definitions of vegetable might include any of the following: an accompaniment to meat or fish; any plant whose fruit, seeds, roots, tubers, bulbs, stems, leaves, or flower parts are used as food; parts of a plant that are savory but not necessarily sweet.

**Educational Purpose:** This exercise will additionally highlight a group of very important vegetable food crops valued for their nutritional attributes. Participants will learn about different vegetable types and some of the important features that contribute to flavor and nutritional value. They will experience the visual, olfactory, taste and tactile qualities and characteristics of this grouping of plants. Salads for tasting will be prepared from the microgreens that were planted in Session 6.

**Social Interactions:** Participants will interact with session leaders and other participants as appropriate. The mechanism for interactions will include questions and comments between leaders and participants. Participants can be asked to share with the group their favorite vegetables and recipes. Participants can also be asked to share preferences in texture of foods.

**Goals/Benefits:** Facilitate participant-plant interactions, increase knowledge; stimulate cognitive abilities and skills; trigger sensory stimulation; promote social interactions, and enjoy activities.

**Materials and Supplies**:

- Scissors
- Bowls
- Utensils
- Salad dressings

**General Activities:**

- For fun, based on the introduction and definition(s) of vegetable, make a list of five vegetables that you can think of other than those already mentioned that are actually fruits, but also considered vegetables.
- Make a table listing of common vegetables according to the part of the plant that is used as a vegetable.
- Pass around various vegetables grown in Florida and explore their sensory aspects such as smell, taste, texture, and appearance
- Harvest the microgreens grown in Session 6 and taste them with some salad dressing
- Discuss some favorite recipes or ways of cooking these vegetables

**Resources:**

- “Florida Gardener’s Handbook” by MacCubbin, Tasker, Bowden and Lamp’l, Cool Spring Press, Minneapolis, MN. (2012) pgs. 85-95
- “Florida Vegetable Gardening Guide” by Sydney Park Brown, James M. Stephens, Danielle Teadwell, Susan Webb, Amanda Gevens, R.A. Dunn, G. Kidder, D. Short and Gary W. Simone. #SP 103, one of a series of the Horticultural Sciences Department, Florida Cooperative Extension Service, Institute of Food and Agricultural Sciences, University of Florida. Original publication date December 1999. Revised December 2010. Reviewed February 2012. Visit the EDIS website at <http://edis.ifas.ufl.edu>

# Appendix of Plant Latin Names per Session

Session 1 - **PROPAGATION: Planting Seeds** **of** **Herbs and Aromatic Plants**

Basil, *Ocimum basilicum* L.

Chives, *Allium schoenoprasum* L.

Cilantro/Coriander, *Coriandrum sativum* L.

Dill, *Anethum graveolens* L.

Oregano, *Origanum vulgare* L.

Rosemary, *Rosmarinus officinalis* L.

Thyme, *Thymus vulgaris* L.

Session 2 - **PROPAGATION: Cuttings of Herbs and Aromatic Plants**

Lavender, *Lavender* spp. L.

Mint, *Mentha* spp. L.

Rosemary, *Rosmarinus officinalis* L.

Scented Geranium, *Pelargonium* spp. (L.) L’Her.

Tarragon, *Artemisia dracunculus* L.

Thyme, *Thymus vulgaris* L.

Session 3 - **TRANSPLANTING: Container Garden Succulent Collection Planting**

Aloe, *Aloe* spp. L.

Echeveria, *Echeveria* spp. DC.

Jade Plant, *Crassula ovate* (Miller) Druce

Kalanchoe, *Kalanchoe* spp. Adans.

Panda toes, *Kalanchoe tomentos* Baker

Stonecrops, *Sedum* spp. L.

Session 4 - **PROPAGATION: Cutting/Division of Herbaceous Ornamentals**

African Violet, *Saintpaulia* spp. H. Wendl.

Begonia, *Begonia* spp. L.

Coleus, *Solenostemon scutellarioides* (L.) R. Br.

Hosta, *Hosta* spp. Tratt

Hoya, *Hoya* spp. R. Br.

Impatiens, *Impatiens* L.

Pilea, *Pilea Peperomioides*

Prayer Plant, *Maranta* spp. L.

Spider Plant, *Chlorophytum comosum* (Thunb.) Jacques

Session 5 - **EDUCATION/TASTING/SENSORY: Herbs and Herb Flavors**

Basil, *Ocimum basilicum* L.

Chives, *Allium schoenoprasum* L.

Cilantro/Coriander, *Coriandrum sativum* L.

Dill, *Anethum graveolens* L.

Mint, *Mentha* spp. L.

Oregano, *Origanum vulgare* L.

Parsley, *Petroselinum crispum* (Mill) Fuss

Rosemary, *Rosmarinus officinalis* L.

Sage, *Salvia officinalis* L.

Thyme, *Thymus vulgaris* L.

Session 6 - **PROPAGATION: Planting Seeds of Fast Germinating Vegetables**

Arugula, *Eruca sativa* Mill.

Garden Cress, *Lepidium sativum* L.

Kale, *Brassica oleracea* Acephala Group

Lettuce, *Lactuca sativa* L.

Radish, *Raphanus sativus* L.

Spinach, *Spinacea oleracea* L.

Swiss Chard, *Beta vulgaris* subsp. cicla L.

Tomato, *Solanum lycopersicum* L.

Session 7 - **TRANSPLANTING: Container Herb/Lettuce Salad Bowl Planting**

Basil, *Ocimum basilicum* L.

Chives, *Allium schoenoprasum* L.

Dill, *Anethum graveolens* L.

Mint, *Mentha* spp. L.

Oregano, *Origanum vulgare* L.

Thyme, *Thymus vulgaris* L.

Arugula, *Eruca sativa* Mill.

Garden Cress, *Lepidium sativum* L.

Kale, *Brassica oleracea* Acephala Group

Lettuce, *Lactuca sativa* L.

Radish, *Raphanus sativus* L.

Spinach, *Spinacea oleracea* L.

Swiss Chard, *Beta vulgaris* subsp. *cicla* L.

Session 8 - **EDUCATION/TASTING/SENSORY: Florida Vegetables and Flavors**

Arugula, *Eruca sativa* Mill.

Bell Pepper, *Capsicum annuum* L.

Broccoli, *Brassica oleracea* Italica Group L.

Carrot, *Daucus carota* subsp. *Sativus* (Hoffm.) Thell.

Celery, *Apium graveolins* var. *dulce* (Mill.) Pers.

Collards, *Brassica oleracea* Acephala Group L.

Cucumber, *Cucumis sativus* L.

Garden Cress, *Lepidium sativum* L.

Kale, *Brassica oleracea* Acephala Group L.

Lettuce, *Lactuca sativa* L.

Onion, *Allium cepa* L.

Radish, *Raphanus sativus* L.

Spinach, *Spinacea oleracea* L.

Swiss Chard, *Beta vulgaris* subsp. *cicla* L.

Tomato, *Solanum lycopersicum* L.

# Glossary of Key Terms

**Asexual propagation:** producing genetically uniform copies of plants via stem, root or leaf cuttings

**Auxin:** a plant hormone produced in the shoot meristems that promotes cell elongation, and often used in asexual propagation to promote rooting

**Bolt:** when a plant starts to produce flower stems; lettuce, basil and spinach are examples of plants inclined to bolt

**Callus:** a mass of unorganized and prolific parenchyma cells produced as a result of wounding. Callus commonly occurs at the cut end of a cutting during the rooting process.

**Chemical biology:** the discipline involving chemistry, biology and physics of biological systems

**Cuttings:** portions of stems, roots, or leaves that are detached from a plant and used to clonally multiply new plants

**Division:** a type of asexual propagation where the plant is sectioned into portions containing shoot and root parts

**Dormancy:** a condition when seeds are blocked from germination by an internal or external mechanism, even in favorable environmental conditions. Seed embryo or seed coat factors can cause different types of dormancy.

**Embryo:** the part of the seed developed from the sexual union of male and female gametes during fertilization

**Evapotranspiration:** the combined process of evaporation of water vapor from soil and plant surfaces into the atmosphere and transpiration or water movement from roots through the plant canopy and out from the leaves

**Fruit:** the mature ovary and seed-bearing structure of a plant by which flowering plants disseminate seeds

**Germination:** the process of seed development beginning with water uptake and leading to radicle protrusion from the seed coating

**Herbaceous plant:** a soft plant that lacks lignified or woody structures

**Meristems:** plant tissue composed of meristematic cells capable of dividing; found in the shoot and root tips and secondary growing points (vascular cambium, cork cambium, leaf marginal meristems)

**Apical Meristem:** meristem found at the highest shoot tip or lowest root tip

**Lateral Meristem:** meristem found below the highest shoot tip or above the lowest root tip

**Microgreens:** the shoots of salad vegetables harvested after the first true leaves have emerged

**Node:** a part of the stem where one or more leaves are held and buds emerge with vegetative or floral organs

**Ovary:** the enlarged basal portion and fleshy tissues of the pistil where the embryo and seed form

**Petiole:** the stalk that holds the leaf to the stem

**Pluripotency:** the concept that a single cell is able to divide and differentiate to produce all of the cells and tissues needed produce a new plant

**Ramets:** the vegetative offspring of a stock plant

**Relative humidity:** the amount of water vapor in the air relative to the amount of water vapor needed to reach saturation at the same temperature

**Rhizome:** a specialized, fleshy, stem structure growing horizontally at, or near, the surface of the ground

**Seed:** the next generation of a plant produced via sexual reproduction. The main parts include an embryo, food storage, and a protective coating.

**Senesce:** the process of aging in plants resulting from normal development or stress

**Sexual reproduction:** male pollen comes into contact with female egg cell in the ovule known as pollination and fertilization, then after recombination of genetics the ovule grows into seeds

**Stolon:** a specialized stem that grows laterally, either above or below ground, from the crown of the plant to produce either another plant or a tuber.

**Transplant shock:** a period of plant stress after transplanting typically expressed by plant wilting and caused by unfavorable weather conditions and a disproportionate root to shoot ratio

**Transplant:** a plant that is moved from one location to another

**Vegetable:** the edible portion of an herbaceous plant including leaves, stems, roots, tubers, bulbs and flowers/floral stalks (broccoli) or fruits

**Xeriphytic plants:** plant that are adapted to dry climatic conditions
