# Supplementary material for: A pilot randomized controlled trial of group-based indoor gardening and art activities demonstrates therapeutic benefits to healthy women
Source: PLoS One. 2022 Jul 6;17(7):e0269248. doi: 10.1371/journal.pone.0269248 (PMC9258874; doi:10.1371/journal.pone.0269248)
Supplement: S1 File — Gardening and Art Protocol Revision 3. (DOCX) [file pone.0269248.s008.docx]

**S2 IRB Approved Trial. Gardening and Art Protocol Revision 3.**

**Title:** “Assessing Human Health Benefits of Gardening”

**Investigators and Study Staff:**

Charles Guy, PhD, Principal Investigator

Raymond Odeh, BS, Study Coordinator

**Abstract:**

The purpose of this study is to investigate the effects of engaging in gardening and art activities on the psychological, social, and physiological characteristics of healthy women as revealed by psychometric assessments and blood pressure and heart rate readings.

**Background:**

There is a limited, but sufficient body of anecdotal and empirical evidence that suggests gardening (Kaplan 1973; Kidd and Brascamp 2002; Park et al 2009; Sommerfeld et al 2010; van den Berg and Custers 2011; Cipriani et al 2017; Soga et al 2017), walking in a park (Hartig et al 2003; Maller et al 2006; Taylor and Kuo 2009) or experiencing nature (Ulrich 1981; 1986; Kaplan 1995; Berman et al 2008; Howell et al 2011; Keniger et al 2013) and similar people-plant interactions are therapeutic. In recent times, it is widely reported in the popular media that activities like gardening and spending time in gardens and nature help reduce stress and contribute to healing (Phillips, 2011; Franklin, 2012; Andrews, 2013). Indeed, gardening or working in the yard is a popular pastime or hobby for millions of adult Americans, being slightly more so for men than women, according to the latest U.S. Department of Labor’s Time Use Survey (ATUS, 2003-2015).

Studies have suggested that visual art, and particularly art depicting scenes of nature, appear to reduce anxiety and stress experienced by patients in hospitals (Ulrich and Gilpin, 2003; Nanda et al., 2010; Ulrich 2009). Ulrich and Gilpin (2003) proposed that visual art in healthcare settings should contain images of flowers, the countryside, landscapes and/or waterscapes. Observed therapeutic benefits attributed to images of nature may link with attention restoration theory (Kaplan 1995) where engagement of directed attention leads to fatigue and natural environments or images of natural environments appear to promote replenishment or restoration of the ability for using directed attention.

While creative arts therapy research has historically been mostly qualitative in approach, there have been studies that have taken a quantitative approach and provided evidence suggesting that for cancer patients there can be therapeutic benefits. Based on systematic review of 11 studies by Uttley and colleagues (2015), there was notable heterogeneity in the data within the art therapy literature leading to inconclusive support for the positive clinical effects of art for individuals with a mental health condition. Studies analyzed tended to be of low quality with small study populations and limited randomized and controlled experimental designs. In contrast, meta-analysis of 13 studies conducted by Boehm and colleagues (2014) indicated significant improvements in levels of anxiety for breast cancer patients compared to controls following art-based interventions. Boehm and colleagues found that treatment effects for depression symptomatology and quality of life were not significant. Another systematic review conducted by Puetz and colleagues (2013) found small to moderate effects in response to music, art, and dance/ movement therapies with regard to psychological outcomes in cancer patients. The review included 27 randomized clinical trials encompassing 1576 cancer patients that had studied the impact of arts interventions on psychological outcomes in cancer patients. The review was performed following guidelines set forth in the Preferred Reporting Items for Systematic Reviews and Meta-Analyses. The analyses showed that pooled arts interventions of the studies revealed reduced anxiety, depression, and with concomitant improved patients’ quality of life. While the evidence of therapeutic benefits accruing from engagement in arts activities is not extensive, there is sufficient empirical support to warrant further investigation regarding the potential therapeutic benefits of this form of intervention.

This proposed study is a follow-on study to a gardening study conducted at Wilmot Gardens in 2015 (IRB 201400425). The previous study revealed therapeutic benefits for study participants in a gardening treatment group compared to a no treatment concurrent control group (unpublished data). In part, this proposed study is designed to follow the earlier gardening study, while improving and reducing potential for bias in the experimental design with the addition of an active concurrent control group, in the form of an arts program. This design model allows for statistical analyses of relative-effect estimates as compared to absolute-effect estimates.

The reasons for selecting an all women, wellness study population are several-fold. There are known cultural, societal and sex differences in the way males and females respond to interactions with plants, and these factors are reflected in part by known consumer patterns. For example, Japanese men were less favorable towards the presence of street-side flowers compared to trees than women (Todorova et al 2004). More women than men agreed with statements that gardening satisfaction is peace/tranquility and plants make me feel calmer/more relaxed in a study of attitudes towards plants and gardening (Relf et al 1992). Women exhibited a stronger flower color preference than men when purchasing geraniums (Behe et al 1999). In a study of images of natural environments versus urban settings, Ulrich and colleagues (1981) found significant sex differences in the responses of women and men with respect to positive affect and attentiveness. Both aspects of psychological status declined more when women viewed urban scenes than they did for men. In a study of garden centers and mass merchandisers, it was found that 85% of shoppers were female and 58% were between the ages of 25 and 55 (Yue and Behe 2009). According to the Society of American Florists, men spend more on floral gifts, and more men than women buy roses and floral arrangements valued above $30. In contrast, women buy a wider range of floral gifts at a lower cost, and more women than men buy less expensive loose or bunched flowers, floral arrangements, flowering plants and dish gardens (<http://www.safnow.org/>).

Additionally, there appears to be evidence for sex differences in stimulus processing in the brain, particularly with respect to responses to various stimuli including visual stimuli (Klein et al 2003; Wrase et al 2003; Smeets et al 2006; Garn et al 2009; Frank et al 2010) in a number of fMRI studies. We believe a study population of a single sex, in this case only women, will reduce experimental variability and increase the probability of detecting a biological effect of the experimental treatment (McCarthy et al 2012). Moreover, it may be that some of the best-documented benefits of working and interacting with plants align well with disorders known to be more prevalent in women such as depression, Multiple Sclerosis, Chronic Fatigue Syndrome and Celiac Disease. We do understand over the time course of the proposed experimental treatment, participants’ menstrual cycle may contribute to variability in the experimental results.

The objectives of this research are three-fold:

1. Ascertain if engaging in a group-based gardening program will differentially alter the mental health status and/or quality of life of healthy women compared to engaging in a group-based art program.
2. Determine if engaging in a group-based gardening program will differentially alter satisfaction with participation in discretionary social activities compared to engaging in a group-based art program.
3. Establish whether blood pressure and/or heart rate will change relative to involvement in a group-based gardening program compared to a group-based art program.

The hypotheses, listed below, will be tested with a well-defined wellness population of women as detailed in a later section of this protocol (see Participant Eligibility Criteria section below). Assessment of the effects of the group-based gardening activity (GA) or art activity (AA) on the experimental populations will take two approaches, the first being the use of wrist cuff blood pressure monitors on the participants to assess systolic and diastolic blood pressure and heart rate that may be improved as a consequence of experiencing the cumulative gardening or art programs and associated stimuli. The second approach will employ widely used and well-established self-reported psychometric assessment instruments that will capture information about the health and well-being of participants.

**Specific Aims:**

The project goal is a better understanding of how working with plants in an indoor gardening program compares with working with art media in an indoor art program with respect to mental health status, satisfaction with participation in social activities, and heart rate and blood pressure patterns. Specifically, this project seeks to determine whether engaging in GAs or AAs can influence overall quality of life of the participants in a wellness population of women, and elicit a physiological effect that can be revealed by changes in blood pressure and heart rate. Assessments will be employed to characterize additional psychometric parameters and determine how they may correlate with changes in blood pressure and heart rate.

Three hypotheses will be tested:

1. Engaging in a group-based gardening program will differentially alter the mental health status and/or quality of life of participants, as compared to engaging in a group-based art program.
2. Improvements in satisfaction with participation in discretionary social activities in the gardening group will parallel improvements observed in the art group.
3. Blood pressure and heart rate of participants will be reduced post-intervention for both the gardening and art groups.

**Research Plan:**

**Experimental Design:** Up to 40 (20 in gardening group and 20 in art group) participants will be recruited to take part in a series of GAs or AAs in experimental treatment sessions to take place over a four to five week period when greenhouse conditions are environmentally favorable. The experiment is designed to test for effects of engaging in group-based gardening or art on blood pressure and heart rate; satisfaction with leisure interests and relationships with friends; mental health which includes dimensions of depression symptomatology, anxiety, stress and mood disorder status; and overall quality of life of the participants. The purpose of this follow-up study is to determine whether there is a difference in treatment response by comparing assessments between the art and gardening groups using both physiological metrics and psychosocial measurements from both groups.

**Participant Eligibility Criteria:** We will select participants from a pool of potential volunteers assembled from respondents to a variety of print and broadcast advertisements, and those expressing interest resulting from word-of-mouth awareness of the recruitment efforts for the study. The study will focus on a wellness population, with similar eligibility criteria as the gardening study conducted in 2015. Participants must be 26-49 years of age, premenopausal women, preferably not pregnant at the time of enrollment in the study, be a non-gardener and non-artist, nonsmoker, in good health and have the ability to participate in all aspects of the study including working with plants in the greenhouse and working with art media in a conference room. Additional eligibility criteria include not having allergies to plants, plant parts like pollen, or plant-based foods, not having an addiction to alcohol or to recreational drugs or prescription medications, not suffering from recurring pain, exhibiting an average BMI less than 32 (a BMI chart will be provided for reference), and not having been diagnosed with any diseases, disorders or disabilities that presently negatively affect daily life. A screening questionnaire has been developed to reveal these health-related criteria (see In-Person Eligibility Screening Questionnaire Gardening and Art Study).

**Study Sample Size:** Based on a set of power calculations from the previous gardening pilot study (IRB201400425), we estimate needing up to 20 individuals in each of the experimental conditions, i.e. gardening program and art program. We will consider the study fully enrolled when 40 study participants are deemed eligible and ready to participate in the experimental intervention. Based on power calculations of psychometric data from the previous gardening study, minimum estimated sample sizes range from 12 to 17 participants required for the gardening experimental condition to achieve sufficient power. However, it is anticipated that perhaps as many as 50% of consented volunteers may fail one or more eligibility requirements, or elect not to continue in the experimental stage of the study.

We anticipate over the experimental treatment duration, that some participants might experience a life event (illness, loss of a loved one, serious accident or similar occurrences) that makes it necessary for them to withdraw from the study prior to study completion. Others may simply decide to cease participating. Some participants may miss some of the sessions. We believe the study, if fully populated, can sustain a 20% attrition rate in either of the two treatment groups and still provide valuable scientific information. Study staff will keep records of participant attendance. We have arbitrarily set an attendance minimum of 6/8 GA or AA sessions for the inclusion of data from a subject. Participants that have a major life event or miss more than two sessions will be allowed to attend all remaining gardening or art sessions. However, no further data from a subject will be collected following the third missed gardening or art session. Any collected data from such participants will be flagged and excluded from data analyses as determined to be scientifically appropriate.

**Staging of Experimental Gardening and Art Programs:** The experimental design for the GA and AA programs will consist of twice weekly group sessions of up to a maximum of 10 participants in an individual session and totaling up to 20 participants overall in each of the treatment group cohorts. Both GA and AA sessions will be approximately 60 minutes in duration and will take place in the Therapeutic Horticulture Greenhouse at Wilmot Gardens or a multi-purpose room in the Conference Center at Wilmot Gardens. Gardening and art sessions will take place on the same day(s) and at the same time. A total of eight individual gardening activity sessions and eight individual art activity sessions will be conducted over a period of four weeks. There will be two GA treatment sessions per week, and two AA sessions per week. Participants will be assigned to attend sessions occurring on Monday and Wednesday or Tuesday and Thursday. If participants are absent from a session, there will be no opportunity to make-up the session. If a study subject misses more than 2 sessions, any further data collected will be excluded from the data set, and the participant will be dropped from the study. An AHTA-registered, master-level horticultural therapist (Co-Principal Investigator) will review each GA experimental treatment planned. A Master’s student and Study Coordinator (Raymond Odeh) and/or the Principal Investigator (Charles Guy) or other study staff member will lead each individual gardening activity session. Dr. Guy will monitor the gardening and art sessions as necessary to confirm experimental comparability. Each art activity will be reviewed and administered by a professional artist(s) and/or experienced personnel in the UF Arts in Medicine program. Each individual gardening and art activity session will tightly follow the same sequence (Table 1).

The experimental design of the study is upgraded from the previous gardening study protocol executed in spring 2015. Previously, the design implemented a protocol in which women self-selected to be in a treatment group or non-active control group. The current design includes the alternate, comparable group of art activity. Eligible volunteers will be screened, consented, selected and enrolled in the study. Participants will then be randomly assigned to receive either the gardening treatment intervention or art intervention. Further participant assignment into two subgroups of gardening and art will be random, but it may be necessary to adjust some assignments to accommodate participant schedules. Both treatment groups will undergo the same self-report psychological and social assessments and physiological measures along the same timeline with congruent intervals of administration.

If study volunteers are eligible, they will be enrolled in the study and invited to attend an orientation session for either the art or gardening groups that includes a tour of the facilities, parking instruction, rules of the experiment, introductions of the group members to study staff and other members of the groups. This is when baseline psychometric assessments will be given.

**Table 1. Sequence Timeline for the Eight Planned Experimental Gardening and Art Sessions.**

**Session Timeline**

| Timeline | Session Component |
| --- | --- |
| 0 min | Arrival and Sign-in/Pick up Name Tag |
| 0-10 min | Individuals Review Previous Activities (i.e. plant growth or art projects)/Instructors Greet Individuals and Assemble Group/Heart Rate and Blood Pressure Measurements Recorded |
| 10-20 min | Educational Module/Introduction to Activity/Demonstration of Activity as Warranted (booklet, instruction sheet, resource material handouts) |
| 20-50 min | Gardening or Art Activity, Questions |
| 50-70 min | Clean-up/Heart Rate and Blood Pressure Measurement Recorded/POMS and PSS Assessments (Even-numbered sessions) and BDI (Sessions 4 and 8)/Departure |
| 70-95 min | Session Review and Record Notes and Observations (Staff only) |

The GA sessions will be conducted inside the Wilmot Gardens 2700 sq. ft. greenhouse to increase control of and uniformity of environmental conditions compared to conducting sessions outside. Ambient environmental conditions for temperature and humidity in the greenhouse will be recorded at the beginning and end of each gardening session to provide information about the conditions over the duration of the gardening treatments. An evaporative cooling system and automated, adjustable shade cloth will be used to keep the greenhouse in a comfortable range for participants, and water fountains are located nearby for participants to keep hydrated throughout the sessions. Outside weather conditions during the scheduled gardening sessions will also be noted as sunny, overcast, rainy, hot or cold, low or high humidity. A restroom is readily accessible in the adjacent lobby of the greenhouse at the front of the structure. Art sessions will be conducted in the air-conditioned Wilmot Gardens Conference Center 1032 sq. ft. conference room that has the capacity to comfortably fit approximately 35 seated people or 10 art session participants and art materials and stands. The conference center is equipped with a kitchenette and accessible bathrooms.

Following the conclusion of each gardening and art session, the leader/study staff of the session will record de-identified notes of informal observations made during the session (see Post-Session Review Form Gardening and Art Study). The review will consider general group interest, engagement and participation in the activities, and note unsolicited feedback from unidentified participants such as “I had fun” or “I liked planting seeds” or “I liked last week’s activity better than today’s,” or whether participants are interactive with other participants in the group activities.

**Gardening Activities (GAs):** Participants assigned to the gardening treatment group will receive eight individual gardening activity sessions (see Gardening Activity Session 1 example in Table 2) over a period of 4 weeks equating to two sessions per week. Additionally, there will be an orientation session before and a wrap-up session after the eight gardening sessions. The individual sessions will take place at the same time of day on Mondays and Wednesdays or on Tuesdays and Thursdays. The individual sessions are designed to fit together within the larger context of the overall experimental treatment. The experimental treatment is configured to introduce and reinforce a limited number of different gardening themes that include: 1) propagation that will be presented in two forms (seeds/bulbs/tubers and cuttings/division); 2) transplanting; and 3) simulated harvest and sensory stimulation. The sensory stimulation theme will be coupled to an educational component about the plants providing the sensory stimulation. The emphasis and reinforcement of the selected gardening themes will be achieved using different types of plants with different uses and characteristics in the different sessions. The participants will be given a free copy of the “Florida Gardener’s Handbook” by MacCubbin, Tasker, Bowden and Lampl’l (2012) (see file of front cover of the book and Table of Contents, Florida Gardeners’s Handbook Cover ToC) that will provide some general background information on the plants used in the GAs. The purpose for supplying the handbook is to provide a more complete gardening and educational experience to complement the hands-on activities in the greenhouse. The activities for all of the individual sessions are designed to involve approximately the same level of physical, cognitive and social engagement to filter out differential competing interacting effects in these aspect areas. Participants will receive a handout booklet (see Gardening Sessions Booklet) for each of the eight sessions that will provide instructions and information for each of the GAs being experienced (see Session 1 Gardening Activity Handout and Instructions Exemplar). Table 2 below provides a detailed example of Session 1 and is generally representative of the information, as well as the educational, social and physical purposes, goals and benefits associated with the eight gardening sessions. A more complete description for all gardening sessions can be found in the supporting documents (see Eight GA and AA Sessions Descriptions) which may undergo some minor changes and adjustments based on availability of plants and/or other gardening materials or as needed to optimize the effectiveness of a given session activity.

**Table 2. Gardening Activity Session 1 Exemplar: Educational Purpose, Anticipated Social Interactions, Specific Goals and Benefits to the Participants.**

**Session and Activities**

| Session 1 | PROPAGATION: Planting Seeds of Herbs and Scented Plants (e.g. basil, borage, chives, dill, oregano, rosemary and thyme)(seedlings can be used for session 7) |
| --- | --- |

**Educational Purpose:** This exercise will highlight a group of plants widely used in culinary practices and prized for their sensory stimulating attributes. Instruction will explain the role and significance of seeds in plant reproduction, propagation, and the development of a plant from a seed, and show variation in plant seed morphology, size, shape and tactile characteristics, demonstrate sowing methods, and observe the rate of germination of different species. Participants will learn how to sow seeds and what materials are needed to begin growing herbs and scented plants for use at home.

**Social Interactions:** Participants will interact with session leaders and co-participants. Mechanism for interactions will include questions and comments between leaders and participants. Participants can be asked to share with the group their favorite herbs and scented plants, including any recipes for herbs. Participants will need to pass seeds to other participants who would like to plant those various varieties. Participants may be asked to work in pairs or small groups in order to share limited materials and tools. In this first session, participants will also be asked to share their first name only with the group.

**Goals/Benefits:** Facilitate participant-plant interactions, increase knowledge about plants and planting seeds; stimulate cognitive abilities and skills; increase concentration, restore directed attention; experience visual, tactile and olfactory sensory stimulation, enhance motor skills (hand-eye coordination and fine motor function); experience group interactions; and enjoy the activity.

____________________________________________________________________________

**Art Activities (AAs):** Participants randomly assigned to the art comparison group will receive eight individual art activity sessions (see Art Activity Session 1 example in Table 3) over a period of four weeks with two sessions per week. Additionally, there will be an orientation session before and a wrap-up session after the eight art sessions. The individual art sessions will take place at the same time of day on Mondays and Wednesdays or on Tuesdays and Thursdays as the gardening sessions. The participants will be given a free copy of the “The Artist’s Handbook” by Ray Smith, 3^rd^ Edition, (2009) (see file of front cover of the book and Table of Contents, The Artist’s Handbook Cover ToC) that will provide some general background information on the art media and tools used in the AAs. The purpose for supplying the handbook is to provide a more complete art and educational experience to complement the hands-on activities taking place in the conference center. The activities for all the individual art sessions are designed to involve approximately the same level of physical, cognitive and social engagement as the gardening intervention in an effort to filter out differential competing interacting effects in these aspect areas. Participants will receive a handout booklet (see Art Sessions Booklet) for each of the eight sessions that will provide instructions and information for each of the AA being experienced (see Session 1 Art Activity Handout and Instructions Exemplar). Table 3 below provides a detailed example of Session 1, and is representative of the information, as well as the educational, social and physical purposes, goals and benefits associated with the eight art sessions. A description for all art sessions can be found in the supporting documents (see Eight GA and AA Session Descriptions) which may undergo some modification or changes and adjustments based on availability of supplies and/or other art materials or as needed to optimize the effectiveness of a given session activity.

**Table 3. Art Activity Session 1 Exemplar: Educational Purpose, Anticipated Social Interactions, Specific Goals and Benefits to the Participants.**

**Session and Activities**

| Session 1 | PAPERMAKING: Making Paper from Recycled Materials |
| --- | --- |

**Educational Purpose:** This exercise will explore basic handmade papermaking techniques and highlight one of the oldest recycling practices. Paper dates back to 105 AD in China and was made by hand until industrial manufacturing in 1803. Hand papermaking continues to be a specialized craft today, and is widely used as a medium for artistic expression. Participants will learn how to create a paper pulp from recycled papers and cotton linter using a household blender. They will experiment with color options and provided additives before pulling sheets of handmade paper using a dip hand mold. Papers will be transferred to couch sheets and left to dry until a subsequent workshop. Participants will also be shown a basic pour method utilizing a tin can mold to increase accessibility of papermaking at home without specialized materials.

**Social Interactions:** Participants will interact with session leaders and other participants. Mechanism for interactions will include questions and comments between leaders and participants. Participants will be sharing pulp vats and additives with one another. In this first session, participants will be asked to share their first name only with the group.

**Goals/Benefits:** Facilitate participant-art medium interactions, increase knowledge; stimulate cognitive abilities and skills; increase concentration; experience visual and tactile sensory stimulation, enhance motor skills (hand-eye coordination); experience group interactions; and enjoy the activity.

____________________________________________________________________________

**The Health and QOL Evaluations:** Gardening programs and horticultural therapy have been linked with reduced levels of anxiety, depression, pain, and stress, and lower heart rate, and improved cognitive function, self-esteem, physical and mental health and quality of life. Similarly, art programs and art therapy have been linked to improvements in positive and negative affect, anxiety, self-esteem, depression symptomatology, and quality of life (Uttley et al 2015). The impacts of the treatment regime on participants will be evaluated with several self-report assessment instruments that have been properly licensed or purchased. The standard SF-36 Health Survey instrument (Ware and Sherbourne 1992; Hays et al 1993) will be used to evaluate eight scales that link to physical and mental health over a four-week recall interval. To gain information relative to stress, the study will use the Perceived Stress Scale (PSS) (Cohen et al 1983) with a recall interval of one week. To gain insight relative to the intensity of depression, the study will use the Beck Depression Inventory 2^nd^ edition (BDI-II) (Beck et al 1988) with a recall interval of two-weeks. To gain information relative to anxiety, the study will use the State Trait Anxiety Inventory (STAI) instrument (Form Y) (Spielberger et al 1983). The STAI reports on *“at this moment”* status. The study will also use the POMS 2-A® short form instrument (McNair et al 1981; Shacham 1983) to assess the mood states of individuals in an effort to examine changes in mood during the course of the treatment. The POMS reports on *“at this moment”* status with regard to mood disturbance. The Profile of Mood States™ provides self-report scales that allow for the assessment of transient, fluctuating feelings, and enduring affect states. The tool is applicable in settings where sensitivity to change is needed to assess treatment progress monitoring and evaluation. The Satisfaction with Participation in Discretionary Social Activities (SPDSA) PROMIS short form v1.0 instrument will be used to assess contentment with participants’ leisure time and affairs with friends, and encompasses a recall interval of one week. The SF-36, PSS, BDI-II, STAI, POMS 2-A Short, and SPDSA instruments will be administered prior to the beginning and following the completion of the gardening and art sessions treatment regime during visits 2 and 11. In addition, for both the GA and AA groups the POM 2-A Short and PSS instruments will be administered on a weekly basis at the conclusion of the 2^nd^, 4^th^, 6^th^ and 8^th^ gardening and art sessions during visits 4, 6, 8 and 10 to provide a time-course of mood and stress status, respectively, during the experiment. The BDI-II will be administered at the conclusion of the 4^th^ and 8^th^ gardening and art sessions during visits 6 and 10 to provide information on the depression status during the experimental regime. Heart rate and blood pressure measurements will be made at the beginning and end of visits 2-11.

**Data Collection:** The study team will collect baseline data on each subject prior to the beginning of the gardening or art activity sessions by administering various assessments during the orientation session, visit 2. In a final wrap-up session, visit 11, we will repeat the data collection, administering the complete battery of assessments following participation in the 8^th^ and final activity session of the program. Assessments of the participants will be in the form of self-administered health-related questionnaires, a discretionary social activities questionnaire, and heart rate and blood pressure monitoring. Trained research study staff will administer questionnaires. Heart rate monitoring for each participant will be accomplished for each session by way of BP wrist cuff readings and heart rate monitoring with the Omron 7 monitor (BP and HR) wearable. Two to three measurements will be taken at the beginning and at the end of each session.

**Other Study Procedures:** If a study participant completes a health and psychometric assessment instrument, and the study staff sees that their responses indicate depressive or anxiety symptomatology, the study staff will recommend that the individual seek professional help.

**Incidental Findings:** No incidental findings are anticipated, but if an incidental finding is made, the participant will be given a direct referral for medical follow-up. The results from the medical follow-up and review will not be provided by the service to the PI.

Anytime a health and psychometric assessment instrument is completed and the study staff sees responses that indicate depressive or anxiety symptomatology, the study staff will recommend the participant seek professional help. In the event that evaluation of the BDI-II or STAI responses suggests a risk of self-harm or suicidal behavior by a study participant, a qualified mental health professional arranged by the study staff irrespective of day and time will immediately evaluate the individual. Study staff will assist as needed to access emergency care at a recognized mental health treatment facility.

**Community Outreach Recruitment Campaign:**

The study recruitment campaign will encompass, as necessary, all of the following recruiting steps that will invite women to contact study staff if interested in participating in the study:

- Distribute and post flyers in the Gainesville community (see Gardening and Art Study Flyer) (e.g. churches, gyms, coffee shops, local bulletin boards, Haile plantation and downtown Gainesville farmers markets, bus stations, college campuses, UF Health complexes including UF Health Springhill, parks e.g. Depot Park). See flyer locations map for more community flyer areas: <https://www.google.com/maps/d/edit?mid=1JS_DWETw3aG-A4Dv2fbO1Wqg95k&ll=29.661801102895165%2C-82.39106939999999&z=11> .
- If necessary, contact local radio stations to read public service announcement script (e.g. WUFT) (see Gardening and Art Study Radio PSA Script).
- If necessary, contact local newspapers to run print advertisement (e.g. Gainesville Sun) (see Gardening and Art Study Print Ad Script).
- Post approved flyer (see Gardening and Art Study Digital Flyer) to newsletters or bulletin boards:
  - Contact the Center for Gender, Sexualities, and Women’s Studies Research to place a study recruitment flyer in their newsletter or send out flyer to mailing list.
  - Contact the University Women's Club organization comprised of faculty and staff and wives of faculty and staff of the University of Florida, and of women who actively support the mission and goals of the Club. Inquire if study could send an announcement for inclusion in their September newsletter. <http://universitywomensclub.ufl.edu/>
  - Contact Alachua County Extension, Master Gardener Coordinators, colleagues in plant sciences, and botanical gardens and send informational flyer about study, asking them if they would distribute flyers or post in their bulletin boards or newsletters.
  - Contact American Association of University Women to provide flyer and information about study, ask to include in newsletter or share with mailing list.
- Provide flyers to UF Health Women's Center – Springhill <https://ufhealth.org/uf-health-womens-center-springhillhill>.
  - And Women and Wellness Classes (North Florida Regional Medical Center).
- Contact UFHealth Integrative Medicine send flyers to <https://ufhealth.org/integrative-medicine/about-us> to make available to wellness classes and distribute around facilities.
- Submit UF StudyConnect request form to Clinical and Translational Science Institute <https://www.ctsi.ufl.edu/research/participant-recruitment/uf-studyconnect/> to display study on UFHealth.org Research Studies and Clinical Trials and UF StudyConnect pages.
- Communicate with HealthStreet: <http://healthstreet.program.ufl.edu/> to coordinate community recruitment.
- Post study details on ResearchMatch.org.
- If recruitment is insufficient following distribution of flyer and radio announcement, we will use a targeted GoogleAd service to target advertisement based on key word searches such as gardening, health and fitness, salad recipes and others to zip codes in Gainesville, FL (see Gardening and Art Study GoogleAd). The GoogleAd Campaign will show the Study ad based on the study flyer, and anyone interested in the study will click on the link (URL) in the ad. The link will be to a webpage on the Environmental Horticulture Department server where it will display the approved Gardening and Art Study Digital Flyer.
- To assist in sharing information about the study, a Gardening and Art Study Information Sheet has been prepared to share with potential volunteers that have requested additional information beyond that contained in the Gardening/Art Study Flyer, but before the Study Staff has contacted an interested party for the scripted phone interview. The information sheet was prepared using content from the approved phone interview script.

**Informed Consent and Screening:**

When interested community members contact study staff by phone, study staff will follow an approved script when returning phone calls (see Gardening and Art Study Phone Script). The phone conversation will include a description of the study, inquiry of the community member’s interest in participation, and assessment of eligibility requirements. Individuals that meet basic eligibility criteria will be invited to schedule an in-person meeting at a private location (a private office or room in Fifield or Mehrhof Hall on the UF campus will be suggested, but other locations will be considered as necessary).

Individuals that decide to attend the in-person meeting will be consented and given an eligibility/screening questionnaire (see In-Person Eligibility Screening Questionnaire Gardening and Art Study) and demographic questionnaire (see Participant Demographic Questionnaire). Study staff will inform the study volunteer that an email and/or phone call will be sent to relay information on volunteer’s status in the study. Volunteers will be given a phone number to contact study staff if they are interested in their status in the study and if they want to follow-up with any questions for the study staff (e.g. asking about which group they have been randomly assigned).

If study volunteers are eligible, they will be randomly assigned to either the gardening or art group and invited to attend an orientation session (visit 2) that includes a tour of the facilities, parking instruction, rules of the experiment, introductions of the group members to study staff and other members of the groups. This is also when baseline psychometric assessments and heart rate and blood pressure measurements will be given.

**Protection of Data:**

Personal Health Information (PHI) will be strictly maintained following the Cyber Security Checklist for Protected Health Information issued by the University of Florida Office of IT Security Management. Once we have completed consenting, we will assign a randomly generated study identification (ID) number. Any collected PHI during the screening process will be kept secure at all times. The PI of the project will create a document linking participant study ID with the participant’s name, and contact information. This study ID document key will be kept in a locked file cabinet in the PI’s office. Electronic copies of this document will be kept on an encrypted drive in an encrypted folder on a server maintained by the department accessible only to department IT administrator authorized users through a password protected computer operating Truecrypt or Bitlocker encryption software. Only the PI (Charles Guy) and student study coordinator (Raymond Odeh) will have access to identifiable PHI. De-identified PHI may be shared with members of the study staff for data analyses as is appropriate.

Participants that expressed permission to contact, but were not eligible to participate in current or future therapeutic horticulture sessions will have their forms destroyed at the end of the study enrollment period.

**Data Preservation and Access:**

The data acquired in the project will include human participant data that require Institutional Review Board approval. All rules and regulations related to privacy (i.e., HIPPA) will be followed with specific regard to collected data.

The electronic data will be preserved in multiple on-site backups in the form of DVDs and RAID hard drive storage. Copies of the (de-identified, see other) electronic data will be preserved online on a departmental server storage space allocated to the Department of Environmental Horticulture. The contents of server storage space is automatically backed up daily, weekly, and monthly. Lost or corrupted data files can be retrieved from the data backup repository. Additional backup on external hard drives stored off campus will not be permitted. No local computer or mobile device will contain files of PHI or any sensitive information.

Original laboratory notebooks will be secured by the PI in his campus office. The data will be archived at the end of the project in the University’s institutional repository, a long-term digital archive containing scholarly or artistic work produced by researchers at the University of Florida.

Research data residing on a secured server will be accessible via VPN and Gatorlink password via the internet. The PI will transfer the research data from this proposed project to the data repository after completion of the research. If patents result from the research, the data will be embargoed until patents have been received. Otherwise, the data will be suppressed until all submitted papers have been published. Research data will be fully accessible for a period of time deemed relevant for the data, typically 5 years for the raw data described above, but not less than three years. Final products such as reports and published materials will be maintained indefinitely or for a period of at least 5 years. In the event the PI leaves the University, the data shall remain at UF for a period of 5 years.

De-identified raw data from the proposed research may be linked to published research article(s) as supplemental information. Data will, in principle, be available for access and sharing as soon as is reasonably possible, and not longer than four years after the acquisition of the data. As described above, the data will be retained for up to 5 years, exceeding the NSF guidelines to preserve the data at least three years beyond the award period, as required by NSF guidelines.

For long-term storage and archiving of analyzed data, the UF ResearchVault can be utilized. The ResearchVault is a secure computing environment that research data can be stored and archived. The ResearchVault is designed to store restricted data including:

• electronic protected health information (ePHI) (HIPAA)

• export-controlled data (ITAR/EAR)

• student data (FERPA)

• controlled unclassified information (CUI)

• intellectual property data (IP)

**Other Data Considerations:**

No significant intellectual property issues involved with the acquisition of the data are anticipated. In the event that discoveries or inventions are made in direct connection with this data, access to the data will be granted upon request once appropriate invention disclosures and/or provisional patent filings are made.

The data acquired and preserved in the context of this proposal will be further governed by the University Florida policies pertaining to intellectual property, record retention, and data management.

Possible Discomforts and Risks:

There is minimal potential from the GA and AA for discomforts and risks beyond what would be experienced while engaging in low exertion gardening or art at home. Participants are not expected to get too hot or too cold because of working in a greenhouse or conference center during the time of year the experiments would take place. There are no risks to participants greater than those individuals would experience in their daily lives at home. GA sessions will only take place after the safe entry period has been satisfied should any pesticides be used in the greenhouse to control pests or diseases during the course of the experiments. Plant materials in the greenhouse treated with pesticides will not be used for any edible purposes.

Plants selected for inclusion in this research study have been screened for potential hazardous properties, and only plants known not to be hazardous have been selected for use. Plants grown for use in the GA by the study group will be free of harmful or hazardous pesticide residues. All plant materials grown by the study group that may be consumed by the participants will be pesticide free, and grown in compliance with established safety standards and recommendations (USDA Food Safety Tips for School Gardens). Some plant materials that are purchased from supermarkets that may be consumed during the experiment may contain some level of pesticide residues that are found on commercially salable produce and fruits.

There is a possible risk of protected health information of participants to be disclosed to unauthorized individuals. The study team will keep electronic files with PHI on a server that can only be accessed by a password-protected computer available only to the PI and study coordinator and appropriately authorized for access by the departmental IT administrator. Paper copies with PHI will be held in locked file cabinets for protection in the PI’s office. Data from ineligible participants (“screening failures”) will be safely shredded upon completion of screening visit.

Possible Benefits:

There are some direct benefits to participating in this research. Participants who complete the gardening program will benefit from learning about the forms, characteristics, sensory features and uses for a variety of plants, plant propagation techniques, planting and cultivation techniques that may contribute to the enhancement of participants’ quality of life. Similarly, participants that complete the art program will benefit from learning about art activities that may also contribute to the enhancement of participants’ quality of life.

**Reimbursement of Participation Costs:** To defray the costs associated with transportation and parking, the study will offer compensation up to the amount of $60 in gift cards for each participant who attends at least six of the gardening sessions, with payments of $20 at visits 2, 6 and 10. Participants in the gardening program will also receive a plant guidebook (“Florida Gardener’s Handbook” by MacCubbin, Tasker, Bowden and Lamp’l, 2012; valued at $24.00 containing information about most of the plants used in the GA. Participants in the gardening program will also be able take home the containerized plantings and individual plants generated from the activities. Total value for take home items from the GAs is estimated at $100 per participant. The participants in the art program will also receive up to $60 compensation, with payments of $20 at visits 2, 6 and 10. Participants in the art program will also be able to take home any art projects they have worked on during the program and an art guidebook (“The Artist’s Handbook” by Ray Smith, 2009) valued at $16.00 containing information about art techniques similar to those learned in the AAs. The value for the take home items for the AAs is estimated at $100. All participants will be asked to a sign a receipt, and study staff will track monetary compensation during the course of the experiments.

Conflict of Interest:

The study team does not have any conflict of interests to disclose.

References:

Andrews, L.W. (2013). Minding the Body: Nine ways to relieve stress by gardening. Psychology Today. April 1, 2013. <http://www.psychologytoday.com/blog/minding-the-body/201304/nine-ways-relieve-stress-gardening> Accessed June 2, 2014.

ATUS 2003-2015. US Department of Labor, Bureau of Labor Statistics and US Department of Commerce, US Census Bureau. American Time Use Survey. <https://www.bls.gov/tus/datafiles_0315.htm>. Accessed June 27, 2017.

Beck, A.T., Steer, R.A. and Garbin, M.G. (1988). Psychometric properties of the Beck Depression Inventory: twenty-five years of evaluation. Clinical Psychology Review. 8: 77-100.

Behe, B., Nelson, R., Barton, S., Hall, C., Safley, C.D. and Turner, S. (1999). Consumer preferences for geranium flower color, leaf variegation and price. HortScience. 34: 740-742.

Berman, M.G., Jonides, J. and Kaplan, S. (2008). The cognitive benefits of interacting with nature. Psychological Science. 19: 1207-1212.

Boehm, K., Carmer, H., Staroszynski, T. and Ostermann, T. (2014). Arts therapies for anxiety, depression, and quality of life in breast cancer patients: A systematic review and meta-analysis. Evidence-Based Complementary and Alternative Medicine. Article ID 103297, 9 page.

Cipriani, J., Benz, A., Holmgren, A., Kinter, D., McGarry, J. and Rufino, G. (2017). A systematic review of the effects of horticultural therapy on persons with mental health conditions. Occupational Therapy in Mental Health. 33: 47-69.

Cohen, S., Kamarck, T. and Mermelstein, R. (1983). A global measure of perceived stress. Journal of Health and Social Behavior. 24: 385-396

Frank, S., Laharnar, N., Kullmann, S., Veit, R., Canova, C., Hegner, Y.L., Fritsche, A. and Preissl, H. (2010). Processing of food pictures: Influence of hunger, gender and calorie content. Brain Research. 1350: 159-166.

Franklin, D. (2012). How hospital gardens help patients heal. Scientific American. 306: 16-17.

Garn, C.L., Allen, M.D. and Larsen, J.D. (2009). An fMRI study of sex differences in brain activation during object naming. Cortex. 45: 610-618.

Hartig, T., Evans, G.W., Jamner, L.D., Davis, D.S. and Gärling, T. (2003). Tracking restoration in natural and urban field settings. Journal of Environmental Psychology. 23: 109-123.

Hays, R.D., Sherbourne, C.D. and Mazel, R.M. (1993). The RAND 36-item Health Survey 1.0. Health Economics. 2: 217-227.

Howell, A.J., Dopko, R.L., Passmore, H.-A. and Buro, K. (2011). Nature connectedness: Associations with well-being and mindfulness. Personality and Individual Differences. 51: 166-71.

Kaplan, R. (1973). Some psychological benefits of gardening. Environment and Behavior. 5: 145-162.

Kaplan, S. (1995). The restorative benefits of nature: Toward an integrative framework. Journal of Environmental Psychology. 15: 169-182.

Keniger, L.E., Gaston, K.J., Irvine, K.N. and Fuller, R.A. (2013). What are the benefits of interacting with nature? International Journal of Environmental Research and Public Health. 10: 913-935.

Kidd, J.L. and Brascamp, W. (2002). Benefits of gardening to the well-being of New Zealand gardeners. Proc. XXVI IHC – Horticulture, Human Well-Being and Life Quality, Ed. D. Relf. Acta Horticulturae. 639, 103-112.

Klein, S., Smolka, M.N., Wrase, J., Gruesser, S.M., Mann, K., Braus, D.F. and Heinz, A. (2003). The influence of gender and emotional valence of visual cues on fMRI activation in humans. Pharmacopsychiatry. 36 Suppl 3: S191-S194.

Maller, C., Townsend, M., Pryor, A., Brown, P. and St. Leger, L. (2006). Healthy nature healthy people: “contact with nature” as an upstream health promotion intervention for populations. Health Promotion International. 21: 45-54.

McCarthy, M.M., Arnold, A.P., Ball, G.F., Blaustein, J.D. and De Vries, G.J. (2012). Sex differences in the brain: The not so inconvenient truth. Journal of Neuroscience. 32: 2241-2247.

McNair, P.M., Lorr, M. and Droppleman, L.F. (1981). POMS manual (2nd ed.). San Diego: Educational and Industrial Testing Service.

Nanda, U., Gaydos, H.L.B., Hathorn, K. and Watkins, N. (2010) Art and posttraumatic stress: A review of the empirical literature on the therapeutic implication of artwork for war veterans with posttraumatic stress disorder. Environment and Behavior. 42: 376-390.

Park, S.-A., Shoemaker, C.A. and Haub, M.D. (2009). Physical and psychological health conditions of older adults classified as gardeners or nongardeners. HortScience. 44: 206-210.

Phillips, A.L. (2011). A walk in the woods. American Scientist. 99: 301.

Puetz, T.W., Morley, C.A. and Herring, M.P. (2013). Effects of creative arts therapies on psychological symptoms and quality of life in patients with cancer. JAMA Intern Medicine. 173: 960-969.

Relf, D., McDaniel, A.R. and Butterfield, B. (1992). Attitudes towards plants and gardening. HortTechnology. 2: 201-204.

Shacham, S. (1983). A shortened version of the Profile of Mood States. Journal of Personality Assessment. 47: 305-306.

Smeets, P.A.M., de Graaf, C., Stafleu, A., van Osch, M.J.P., Nievelstein, R.A.J. and van der Grond, J. (2006). Effect of satiety on brain activation during chocolate tasting in men and women. American Journal of Clinical Nutrition. 83: 1297-1305.

Soga, M., Gaston, K.J. and Yamaura, Y. (2017). Gardening is beneficial for health. A meta-analysis. Preventive Medicine Reports. 5: 92-99.

Sommerfeld, A.J., Waliczek, T.M. and Zajicek, J.M. (2010). Growing minds: Evaluating the effect of gardening on quality of life and physical activity level of older adults. HortTechnology 20: 705-710.

Speielberger, C.D., Gorsuch, R.L. and Lushene, R. (1983). The state-trait personality Inventory STAI-Y, form Y. Consulting Psychologists press, Palo Alto.

Taylor, A.F. and Kuo, F.E. (2009). Children with attention deficits concentrate better after walk in the park. Journal of Attention Disorders. 12: 402-409.

Todorova, A., Asakawa, S. and Aikoh, T. (2004). Preferences for and attitudes towards street flowers and trees in Sapporo, Japan. Landscape and Urban Planning. 69: 403-416.

Ulrich, R.S. (1981). Natural versus urban scenes: Some psychophysiological effects. Environment and Behavior. 13: 523-556.

Ulrich, R.S. (1986). Human responses to vegetation and landscapes. Landscape and Urban Planning. 13: 29-44.

Ulrich, R.S. (2009). Effect of viewing art on health outcomes. S.B. Frampton, P.A. Charmel (Eds.), Putting patients first: Best practices in patient centered-care (pp. 129–149). Jossey Bass, San Francisco

Ulrich, R.S. and Addoms, D.L (1981). Psychological and Recreational Benefits of a Residential Park. Journal Leisure Research. 13: 43-65

Ulrich, R.S. and Gilpin, L. (2003). Healing arts: Nutrition for the soul. In S. B. Frampton, L. Gilpin & P. Charmel (Eds.), Putting patients first: Designing and practicing patient centered care (pp. 117-146). Jossey-Bass :San Francisco.

USDA Food Safety Tips for School Gardens, Memo SP 32-2009. USDA Food and Nutrition Service. <https://www.fns.usda.gov/sites/default/files/foodsafety_schoolgardens.pdf>. Accessed on June 27, 2017.

Uttley, L., Scope, A., Stevenson, M., Rawdin, A., Buck, E.T., Sutton, A., Stevens, J., Kaltenthaler, E., Dent-Brown, K. and Wood, C. (2015). Systematic review and economic modelling of the clinical effectiveness and cost-effectiveness of art therapy among people with non-psychotic mental health disorders. Health Technology Assessment, 19 (18).

Van Den Berg, A.E. and Custers, M.H.G. (2011). Gardening promotes neuroendocrine and affective restoration from stress. Journal of Health Psychology. 16: 3-11.

Ware, J.E. and Sherbourne, C.D. (1992). The MOS 36-item short-form health survey (SF-36). I. Conceptual framework and item selection. Medical Care. 30: 473-483

Wrase, J., Klein, S., Gruesser, S.M., Hermann, D., Flor, H., Mann, K., Braus, D.F. and Heinz, A. (2003). Gender differences in the processing of standardized emotional visual stimuli in humans: a functional magnetic resonance imaging study. Neuroscience Letters. 348-41-45.

Yue, C. and Behe, B.K. (2009). Factors affecting U.S. consumer patronage of garden centers and mass-merchandisers. Proc. XVIth IS on Hort. Econ. & Manag. Ed.: P.P. Oppenheim. Acta Horticulturae. 831: 301-308.
